# Loss of Erbin in tubular epithelial cells sensitizes mice to acute kidney injury and aggravates renal fibrosis

**DOI:** 10.1038/s44318-026-00885-y

**Published:** 2026-08-24

**Authors:** Fang Shao, Zhengrong Ren, Lei Shi, Yanyan Sun, Wen Sun, Jing Tang, Yingsailamu, Zhi Ding, Junfeng Zhang, Yuhui Zang

**Affiliations:** State Key Laboratory of Pharmaceutical Biotechnology, School of Life Sciences, Nanjing University, Nanjing, 210093 PR China

**Keywords:** Cell Adhesion, Polarity & Cytoskeleton, Cell Cycle, Molecular Biology of Disease

## Abstract

Aging is a major contributor to the increasing incidence of acute kidney injury (AKI) and chronic kidney disease (CKD), however the molecular events explaining aging-related nephropathies remains elusive. Here, we found that the RAS signaling adapter protein Erbin (ErbB2-interacting protein) is abundantly expressed in renal tubular cells, but its levels are decreased in murine AKI models and human CKD patient samples. Tubule-specific deletion of Erbin rendered mice highly susceptible to acute kidney injury, and exacerbated renal fibrosis. In contrast, restoring Erbin protected against AKI and CKD. Moreover, tubular Erbin loss in the naturally-aged kidney aggravates aging-associated nephropathies. Transcriptome sequencing revealed a causal association between Erbin loss and cell-cycle alterations in tubular epithelial cells. Depleting Erbin increased the proportion of tubular cells in G2/M phase and accelerated cellular senescence in vitro and in vivo. Mechanistically, Erbin protects tubular cells from senescence by stabilizing YB-1, which maintains CDK1 and cyclin B1 expression, and facilitates cell-cycle progression. Thus, restoring Erbin represents a potential strategy for chronic kidney disease and aging nephropathies.

## Synopsis

The molecular events initiating aging-related nephropathies such as acute kidney injury (AKI) and chronic kidney disease (CKD) remain elusive. This study shows that loss of ErbB2-interacting protein (Erbin) triggers senescence in renal tubule cells by destabilizing YB-1 and impairing cell-cycle progression.

- Erbin is predominately expressed in the tubular epithelial cells and is decreased during the progression of AKI, CKD, and kidney aging.
- Tubule-specific deletion of Erbin sensitizes mice to AKI, exacerbates renal fibrosis, and accelerates kidney aging.
- Loss of Erbin increases the proportion of G2/M-phase cells and leads to cellular senescence in tubular epithelial cells.
- Erbin protects tubular cells from senescence by stabilising cell-cycle regulator YB-1.

## Abstract (web summary)

ErbB2-interacting protein Erbin protects from senescence and fibrosis during renal aging by stabilizing YB-1 and ensuring cell cycle progression.

## Graphical abstract

**Figure:**
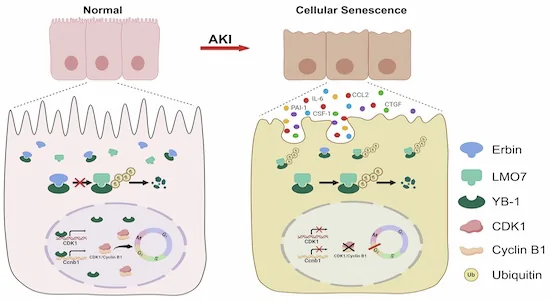

## Introduction

Along with life expectancy, the incidence rates of acute kidney injury (AKI) and chronic kidney disease (CKD) have continually grown worldwide. Aging kidney undergoes structural changes resembling CKD. These changes render the aged kidney highly susceptible to various AKI, and greatly increase the propensity to develop CKD (Ferenbach and Bonventre, 2015; O’Sullivan et al, 2016; Zuk and Bonventre, 2016). Nevertheless, the key initiating events and the common downstream pathways that might be shared by aging nephropathy and AKI/CKD under physiological or pathophysiological conditions remain elusive.

Regardless of the etiology, renal tubules are the epicenter of early kidney disease. The tubular epithelial cells (TECs), in particular the proximal tubular epithelial cells, are exquisitely sensitive to ischemic, septic, or toxic insults, and are largely responsible for repairing the injured tissues (Bonventre and Yang, 2011). In response to damage, the surviving TECs reversibly revert to a dedifferentiation state, and repopulate the lost tubule cells (Chang-Panesso and Humphreys, 2016; Kusaba et al, 2014). Nevertheless, severe and repeated episodes of AKI often cause impaired or maladaptive repair, and consequently, lead to senescence of tubular cells characterized by cell cycle arrest (Huang et al, 2022; Yang et al, 2010). The resulting tubular cell dysfunction is the hallmark of AKI and CKD (Linkermann et al, 2014).

Senescence describes a status of permanent cell cycle arrest triggered by a variety of stresses (Di Micco et al, 2021; Gorgoulis et al, 2019). The accumulation of senescent tubular cells has been extensively identified in various types of CKD (Li et al, 2023; Liu et al, 2012a; Kakoki et al, 2006; Sis et al, 2007). The senescent tubular cells acquire a senescence-associated secretory phenotype (SASP) characterized by the secretion of profibrotic and pro-inflammatory factors, which initiate renal fibrogenesis and promote the progression of CKD in a paracrine manner (Luo et al, 2018; Yang et al, 2010; Zhang et al, 2023). Despite these findings, the underlying mechanisms controlling tubular cell senescence in normal and pathological conditions remain largely unknown.

YB-1 is an evolutionarily conserved cold-shock protein that exerts pleiotropic cellular functions by regulating target gene expression at transcriptional or translational levels (Alkrekshi et al, 2021). Notably, YB-1 is essential for cell cycle modulation due to the ability to promote cyclins (Ccns) and cyclin-dependent kinases (CDKs) expression, or inhibit the checkpoint proteins p21 and p16 (Basaki et al, 2010; Jurchott et al, 2003). Inhibiting YB-1 causes cell cycle arrest, whereas maintaining YB-1 expression protects cells from senescence (Kotake et al, 2017; Wang et al, 2021). For instance, YB-1 inhibits the translation of a subset of senescence-associated mRNAs via binding to their 3’ untranslated regions, and, therefore, protects the epidermal progenitor from senescence (Kwon et al, 2018). The RNA-binding protein UNR/CSDE1 enables the oncogene-induced senescence in primary mouse keratinocytes by reducing YB-1 (Avolio et al, 2022).

ErbB2-interacting protein, best known as Erbin, is a member of the leucine-rich repeat (LRR) and PSD95/discs large/ZO-1 (PDZ) domain (LAP) protein superfamily. In addition to ERBB2, more than 20 proteins have been found to interact with Erbin via the LRR or PDZ domain (Borg et al, 2000; Jang, et al, 2021). Mechanistically, Erbin serves as a molecular platform to organize or modulate particular signaling activity at a desired site by mediating the protein-protein interaction (Good et al, 2011). Depending on specific cell types and contexts, Erbin functions as a tumor suppressor or promoter in colon, breast, liver, and lung cancer (Dardousis et al, 2007; Shen et al, 2021; Tao et al, 2014; Wu et al, 2017). To date, the role of Erbin in kidney disease remains unknown. Given the role of LAP family members in determining epithelial integrity (Bilder et al, 2000; Bryant and Huwe, 2000), it would be of great interest to investigate whether Erbin is involved in regulating the fate of TECs under physiological and pathophysiological conditions.

In the present study, we found that the abundant Erbin protects tubular cells from senescence by directly interacting with YB-1 and maintaining its stability. The loss of Erbin in tubular epithelial cells sensitizes mice to AKI and aggravates renal fibrosis.

## Results

### Erbin is predominantly expressed in the tubular epithelial cells and is decreased in the progression of AKI and CKD

**Figure 1:**
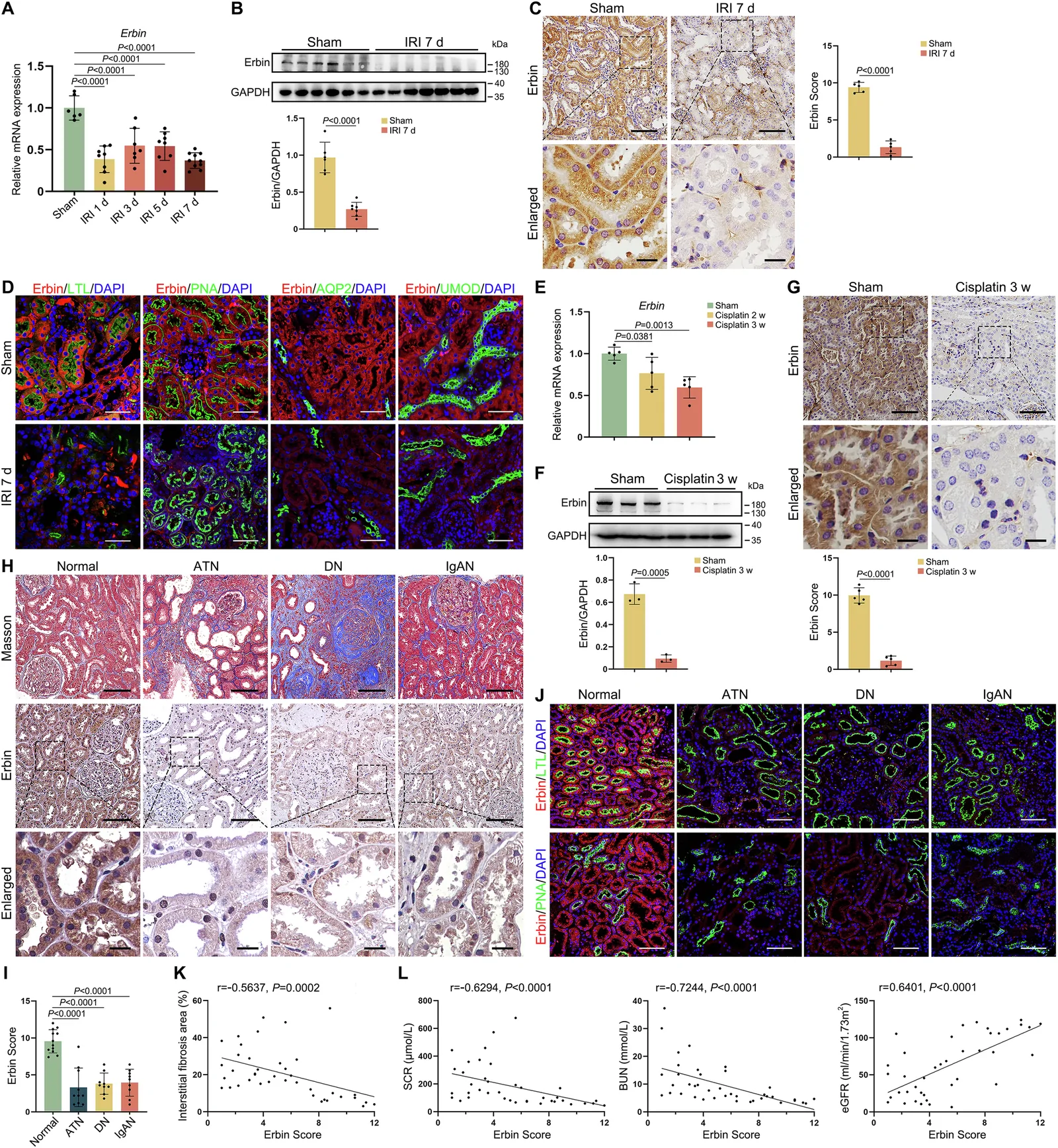
Erbin is predominantly localized in the tubular cells and is decreased after kidney injury. (**A**, **B**) Erbin mRNA and protein expression is decreased after I/R in mice. The relative mRNA expression is normalized against *Actb* unless noted otherwise in this study. The results are shown as fold change compared with the sham group. Relative protein level was calculated by band intensity against GAPDH, unless noted otherwise in this study. *n* = 6 for Sham, *n* = 8 for IRI 1 day, *n* = 7 for IRI 3 day, *n* = 8 for IRI 5 day, and *n* = 10 for IRI 7 day. (**C**) Representative images of Erbin IHC staining in the IRI-induced renal cortex (scale bar, 100 μm). The bottom are enlarged from the boxed areas in the top images (scale bar, 25 μm). The dot plot shows the score of Erbin staining (right). (**D**) Representative images of IRI-induced cortical kidney sections stained for Erbin with LTL, PNA, AQP2, or UMOD (Scale bar, 100 μm). Nuclei were visualized using DAPI staining. (**E**, **F**) Erbin is downregulated in the kidney after administration of cisplatin (*n* = 5 per group). (**G**) Representative images of Erbin IHC staining in cisplatin-treated renal cortex (scale bar, 100 μm). *n* = 5 per group. The bottom are enlarged from the boxed areas in the top images (scale bar, 25 μm). The dot plot shows the score of Erbin staining (bottom). (**H**) Representative images of Masson staining and Erbin staining of kidney biopsies from ATN (*n* = 10), DN (*n* = 9), and IgAN (*n* = 9) patients (scale bar, 100 μm). Nontumor kidney tissues from the patients who had renal cell carcinoma and underwent nephrectomy were used as normal controls (*n* = 12). The bottom areas are enlarged from the boxed areas in the middle images (scale bar, 25 μm). (**I**) Score for Erbin staining in human kidney biopsies. *n* = 12 for normal controls, *n* = 10 for ATN, *n* = 9 for DN, and *n* = 9 for IgAN. (**J**) Representative images of human kidney sections stained for Erbin and LTL, or Erbin and PNA (Scale bar, 100 μm). Nuclei were visualized using DAPI staining. (**K**) Scatter plots show the negative correlation of Erbin score with Masson-stained interstitial fibrosis area in the specimens from CKD patients. Data points represent measurements of individual patients (*n* = 40). The Spearman correlation coefficient (r) is shown. (**L**) Erbin expression is negatively correlated with SCR and BUN, and is positively correlated with eGFR in the specimens from CKD patients (*n* = 40). The Spearman correlation coefficient (r) is shown. Data were presented as the mean ± s.e.m. *P* values were determined using one-way ANOVA with Dunnett’s multiple comparison test in (**A**, **E**, **I**), and two-tailed unpaired Student’s *t*-test in others. Source data are available online for this figure.

We first examined Erbin in the murine model of ischemia/reperfusion (I/R)-induced kidney injury (IRI), and found a significant decrease of Erbin after I/R (Fig. 1A,B). Notably, the Erbin product is predominantly located in the tubular cells (Fig. 1C). We then isolated the primary tubular cells from the IRI kidney and confirmed a significant decrease of Erbin in the tubular cells (Appendix Fig. S1). Furthermore, dual staining with segment-specific markers revealed abundant expression of Erbin in proximal and distal tubules, but no detectable overlaps with collecting duct, ascending loop of Henle, or distal convoluted tubule (Fig. 1D). The loss of Erbin in TECs was also observed after administration of cisplatin (Fig. 1E–G).

Next, we performed an in vitro model to mimic renal ischemia using the rat renal proximal tubular epithelial cell NRK-52E, and found a gradual decrease of Erbin after hypoxia/re-oxygenation treatment. When NRK-52E cells were incubated with H_2_O_2_ or cisplatin to induce oxidative stress or cellular injury, we observed similar phenomena (Appendix Fig. S2).

Similar to animal models, Erbin is abundantly expressed and predominantly localized in the healthy tubular epithelium, but greatly decreases in the specimens from CKD patients with different nephropathies, including acute tubular necrosis (ATN), IgA nephropathy (IgAN), and diabetic nephropathy (DN) (Fig. 1H–J; Appendix Fig. S3; Appendix Table S1). In addition, tubular Erbin level was inversely correlated with the extent of renal fibrosis and the decline of renal function (Fig. 1K,L).

### Tubule-specific deletion of Erbin sensitizes the mice to AKI and deteriorates interstitial fibrosis

We crossbred the Ksp-Cre and Erbin^flox/flox^ mice, and confirmed the genotypes of tubule-specific Erbin null (KspCre^+/-^Erbin^fl/fl^, referred to as Erbin-KO) and the littermate wild-type (WT) (KspCre^+/-^Erbin^wt/wt^) mice (Appendix Fig. S4A). To affirm the specificity of Cre-recombinase expression, we crossed Ksp-Cre mice with the mG/mT reporter mice, showing the tdTomato expression representative of Cre activity in the tubular epithelia (Appendix Fig. S4B). Co-immunostaining with the segment-specific markers further confirmed the Cre activity in both proximal and distal tubules (Appendix Fig. S4C). Afterward, we validated the deletion of Erbin in the tubular cells of Erbin-KO mice (Appendix Fig. S4D–F).

**Figure 2:**
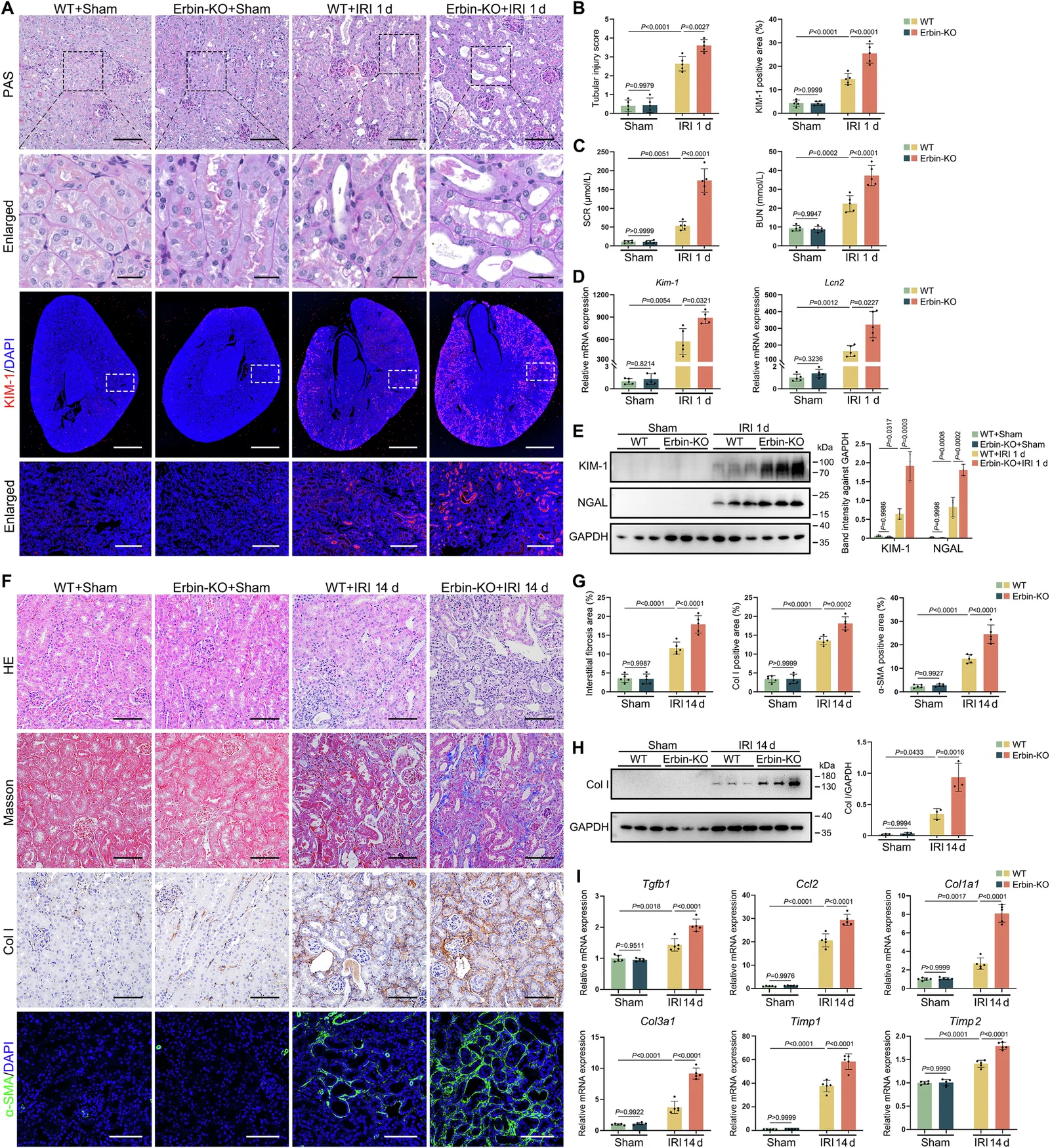
Tubule-specific Erbin deletion sensitizes mice to IRI-induced tubular damage and interstitial fibrosis. The tubule-specific Erbin knockout (Erbin-KO) and WT mice were sacrificed at 1 and 14 days after I/R, respectively (*n* = 5 per group). (**A**) PAS staining and KIM-1 immunostaining showing exacerbated epithelial damage in Erbin-deficient mice at one day after I/R. Scale bar for PAS staining, 100 μm, and the boxed areas are enlarged (Scale bar, 25 μm). KIM-1 immunostaining images of the whole kidney cross-sectional area are stitched at ×200 magnification (Scale bar, 1000 μm), and the bottom panels are enlarged from the boxed areas in the stitched images (scale bar, 100 μm). (**B**) Tubular injury score and quantification of KIM-1 staining in cortex and outer medulla from whole kidney section images. (**C**) SCR and BUN levels at 1 day after I/R. (**D**, **E**) KIM-1 and NGAL expression are significantly augmented in Erbin-deficient mice at one day after I/R, as indicated by qRT-PCR and western blot. (**F**) Representative images of HE, Masson staining, immunohistochemistry for collagen I and immunostaining for α-SMA in kidneys. Scale bars, 100 μm. (**G**) Quantitative analysis of tubular interstitial fibrosis from Masson staining, and quantification of Col I and α-SMA staining area. (**H**) Kidney collagen I was detected by Western blot. (**I**) The mRNA expression of kidney Tgfb1, Ccl2, Col1a1, Col3a1, Timp1, and Timp2 were examined by qRT-PCR. The results are shown as fold change compared with WT mice without operation. Results are presented as the mean ± s.e.m. *P* values were determined using Brown–Forsythe and Welch ANOVA with Tamhane’s multiple comparisons test in (**D**), and one-way ANOVA with Tukey’s multiple comparison test in others. Source data are available online for this figure.

The Erbin-KO mice did not show developmental abnormalities and noticeable changes in tubular structure and renal function at the age of 3 months (Fig. 2A–C). Nevertheless, tubular-specific Erbin deficiency sensitized the mice to IRI, which was characterized by the exacerbated tubular damage and the increased necrotic area at one day after I/R (Fig. 2A,B; Appendix Fig. S5). Consistently, serum creatinine (SCR) and blood urea nitrogen (BUN) levels, as well as KIM-1 and NGAL, the well-known markers of tubular damage, were significantly augmented in Erbin-KO mice as compared to the WT control (Fig. 2A–E). Notably, Erbin-KO mice exhibited the exacerbated tubular damage and necrotic cell death as early as 12 h after I/R (Appendix Fig. S6). When subjected to cisplatin-induced AKI, Erbin-KO mice also exhibited exacerbated tubular damage, deteriorated renal function, and augmented KIM-1 and NGAL expression as compared to WT mice (Appendix Fig. S7).

Finally, tubule-specific Erbin deficiency markedly aggravated the extent of IRI-induced interstitial fibrosis at 14 days after I/R (Fig. 2F–I). The increase of profibrotic factors, as well as the staining area of α-SMA, was significantly augmented in the IRI kidney of Erbin-KO mice, reflecting a substantial effect of tubular Erbin deficiency on augmenting the activation of interstitial fibroblast (Fig. 2F,G,I).

### Ectopic expression of Erbin alleviates AKI and reduces interstitial fibrosis in mice

**Figure 3:**
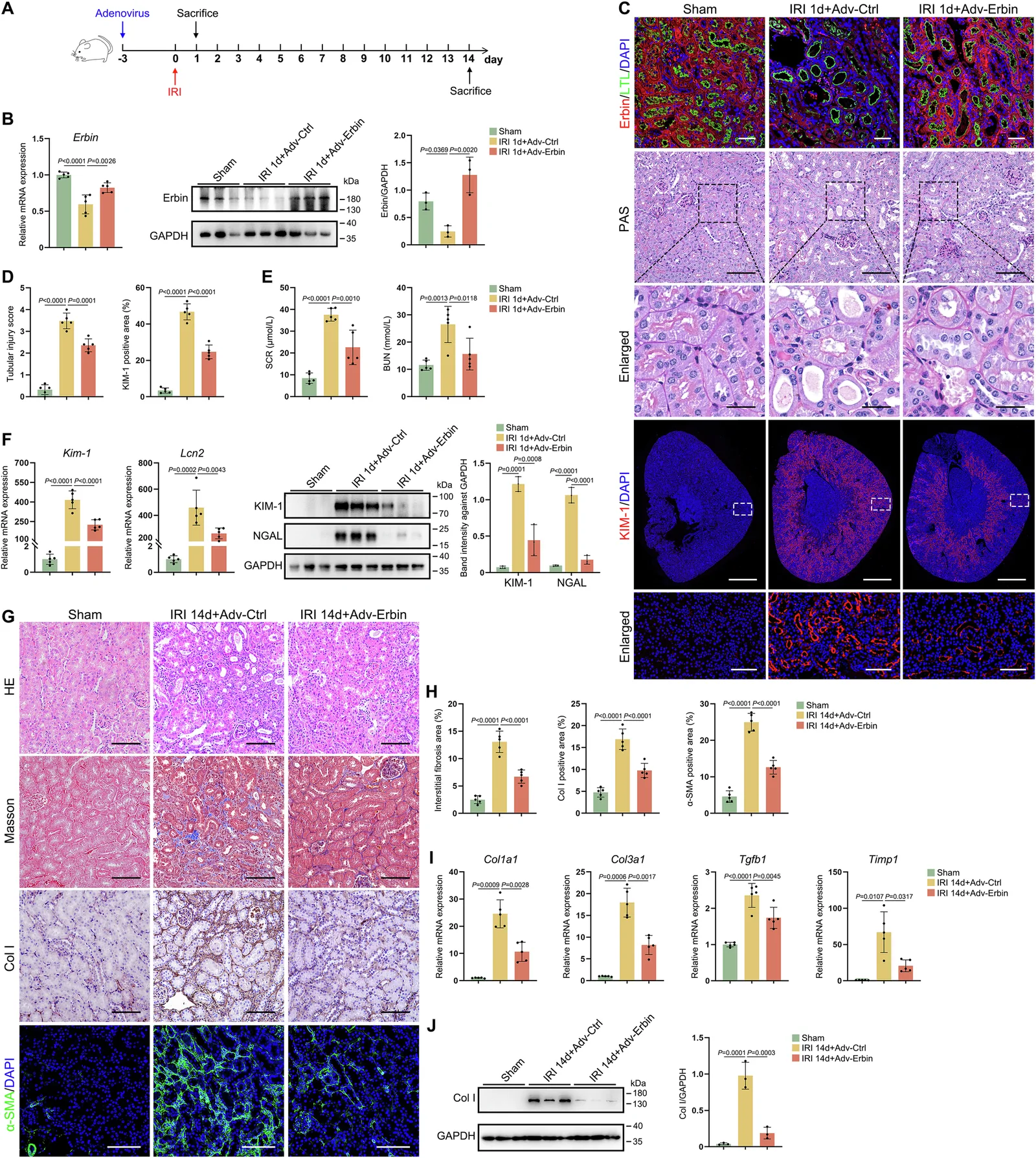
Ectopic expression of Erbin protects mice from IRI-induced tubular injury and subsequent renal fibrosis. (**A**) Schematic diagram of the IRI model with ectopic expression of Erbin. The adenovirus was administered in situ in the cortex of the kidney one week before I/R. Kidneys were harvested on day 1 or 14 after I/R (*n* = 5 per group). (**B**) Overexpression of Erbin in the cortex was examined by qRT-PCR (left) and western blot (right). (**C**) Overexpression of Erbin reduced the severity of tubular injury at 1 day after I/R. Kidney sections were co-stained for Erbin and LTL (Scale bar, 100 μm). Representative images of PAS staining showing that Erbin overexpression reduces epithelial damage (Scale bar, 100 μm). The boxed areas are enlarged (Scale bar, 25 μm). KIM-1 immunostaining images of the whole kidney cross-sectional area are stitched at ×200 magnification (Scale bar, 1000 μm). The boxed areas are enlarged (scale bar, 100 μm). (**D**) Tubular injury score and quantification of KIM-1 staining in cortex and outer medulla from whole kidney section images. (**E**) SCR and BUN levels at 1 day after I/R. (**F**) KIM-1 and NGAL expression were examined by qRT-PCR (left) and western blot (right), respectively. (**G**) Representative images of HE, Masson staining, immunohistochemistry for collagen I, and immunostaining for α-SMA in mouse kidneys. Scale bars, 100 μm. (**H**) Quantitative analysis of tubular interstitial fibrosis from Masson staining, and quantification of Col I and α-SMA staining area. (**I**) The mRNA expression of kidney Col1a1, Col3a1, Tgfb1, and Timp1 were examined by qRT-PCR. The results are shown as a fold change compared with mice without the operation. (**J**) Kidney collagen I was detected by Western blot. Results are presented as the mean ± s.e.m. *P* values were determined using one-way ANOVA with Dunnett’s comparison test for (**B**, **D**, **E**, **F**, **H**), and Brown–Forsythe and Welch ANOVA with Dunnett’s T3 multiple comparisons test in (**I**, **J**). Source data are available online for this figure.

We performed a gain-of-function experiment by in situ injecting the adenovirus capable of overexpressing Erbin (referred to as Adv-Erbin) into the cortex 3 days before I/R (Fig. 3A). The efficiency of virus delivery was assessed by a strong fluorescence of co-expressing enhanced green fluorescent protein (EGFP) in the tubule (Appendix Fig. S8). Ectopic expression of Erbin in the tubular epithelium markedly alleviated the IRI-induced tubular injury, reduced necrotic area, restored renal function, and inhibited the upregulation of KIM-1 and NGAL as compared to the control virus-injected mice (Fig. 3B–F; Appendix Fig. S9). Moreover, injection of Adv-Erbin significantly prevented the activation of interstitial fibroblast, and reduced collagen deposition in renal interstitium (Fig. 3G,H). In line with the morphologic alterations, profibrotic factors and collagen were significantly inhibited in the Adv-Erbin-injected IRI kidney (Fig. 3I,J).

### Deletion of Erbin increases the proportion of G2/M phase and leads to cellular senescence in TECs

**Figure 4:**
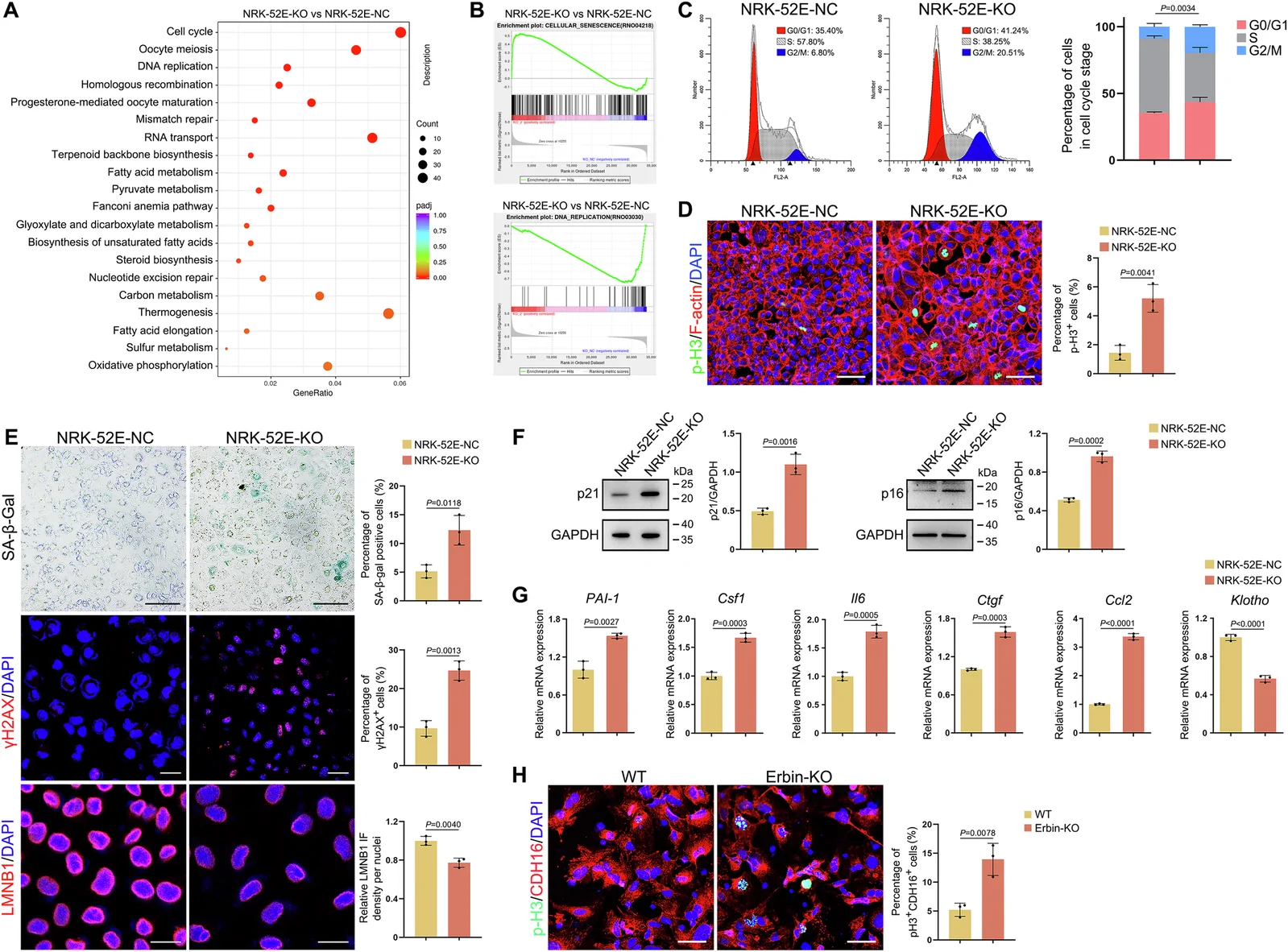
Erbin knockout increases the proportion of G2/M phase and leads to cellular senescence in TECs. (**A**) KEGG analysis showing the top 20 most enriched pathways in the classification of downregulated DEGs in Erbin knockout NRK-52E cells in comparison with control. (**B**) Gene set enrichment analysis of the differentially expressed genes highlighting significant enrichment for the cellular senescence and DNA replication processes. (**C**) Flow cytometry assay showing that knockout of Erbin increased the proportion of G2/M phase in NRK-52E cells (*n* = 3). (**D**) Immunostaining for p-H3 (Green). F-actin was co-stained (Red). Nuclei were visualized using DAPI staining (blue). Scale bar, 50 μm. The dot plot shows the percentage of p-H3^+^ cells (*n* = 3). (**E**) SA-β-gal staining (scale bar, 100 μm), γH2AX immunostaining (scale bar, 50 μm), and LMNB1 immunostaining (scale bar, 20 μm) of Erbin knockout NRK-52E cells. Nuclei were visualized using DAPI staining. Dot plots show the corresponding quantification of SA-β-gal, γH2AX and LMNB1 staining (*n* = 3). LMNB1 intensity was measured across each identified nuclear area and shown as a fold change relative to the control cells. (**F**) Western blot analysis of p21 and p16. (**G**) The mRNA expression of *PAI-1*, *Csf1*, *Ctgf*, *Il6*, *Ccl2,* and *Kloth*o in Erbin knockout NRK-52E cells was examined by qRT-PCR. The results are shown as fold change relative to NRK-52E control cells (*n* = 3). (**H**) Dual immunostaining of p-H3 and CDH16 for the primary TECs isolated from Erbin knockout mice. scale bar, 50 μm. The dot plot shows the percentage of pH3^+^CDH16^+^ cells (*n* = 3). Results are presented as the mean ± s.e.m. *P* values were determined using a two-tailed unpaired Student’s *t*-test. Source data are available online for this figure.

To investigate the role of Erbin in TECs, we generated the Erbin-deficient NRK-52E sublines (referred to as NRK-52E-KO and NRK-52E-NC for control) using a CRISPR/Cas9 approach (Appendix Fig. S10A–C), and performed transcriptome sequencing. Functional annotation by Gene Ontology (GO) on biological process (BP) identified “DNA replication” and “chromosome organization” as the top terms in the downregulated differentially expressed genes (DEGs), whereas “regulation of cell death” was one of the most enriched terms in the upregulated DEGs (Appendix Fig. S10D). Of note, KEGG (Kyoto Encyclopedia of Genes and Genomes) analysis demonstrated that “cell cycle” was the most significantly enriched pathway in the downregulated DEGs (Fig. 4A). Gene set enrichment analysis (GSEA) further showed that the pathways associated with cellular senescence remained elevated, whereas the DNA replication pathway was inhibited after Erbin deletion (Fig. 4B).

Hence, we examined cell cycle in NRK-52E-KO cells by flow cytometry, and found a significant increase of cells in the G2/M phase as compared to the control (Fig. 4C). This observation was confirmed by the increase of phospho-histone H3 at serine10 (p-H3) (Fig. 4D; Appendix Fig. S10E). Accordingly, the proliferative potential of NRK-52E-KO cells was significantly inhibited (Appendix Fig. S10F,G). When examining cellular senescence, we found a significant increase of senescence-associated β-galactosidase (SA-β-gal) activity, along with much higher p21, p16 and phosphorylated histone H2AX (γH2AX), but lower LMNB1in NRK-52E-KO cells (Fig. 4E,F). The representative SASP markers were increased after Erbin deletion, whereas the antiaging Klotho was inhibited (Fig. 4G). In addition, deletion of Erbin sensitized the tubular cells to insult-induced cellular senescence (Appendix Fig. S10H,I). In comparison with the control cells, the cell viability of NRK-52E-KO cells was further inhibited when they were challenged to various insults (Appendix Fig. S10J).

**Figure EV1:**
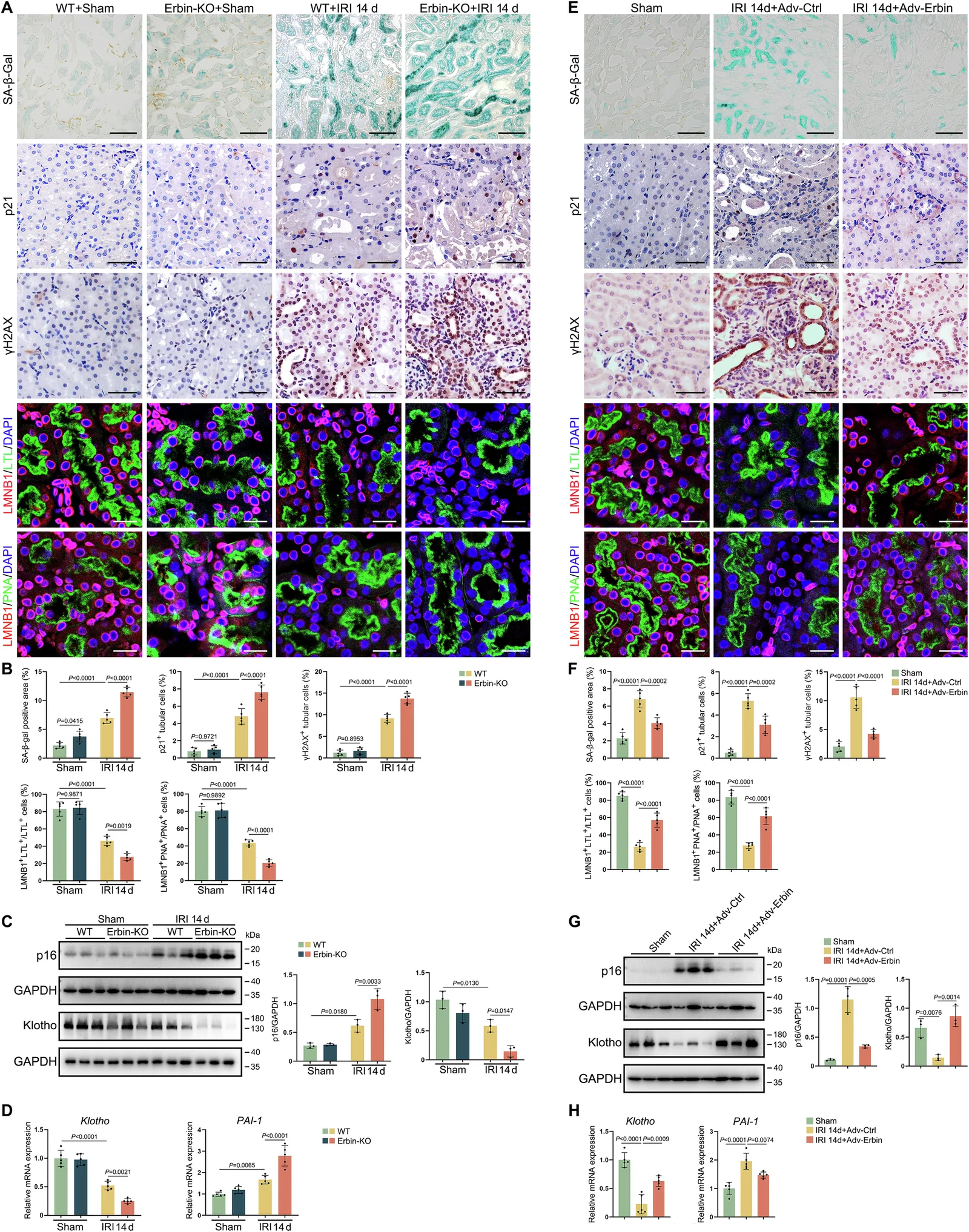
Erbin is implicated in tubular cell senescence in the IRI kidney. (**A**) Representative images of SA-β-gal, p21, γH2AX, and LMNB1 staining from kidneys of WT and Erbin knockout mice on day 14 after I/R. LMNB1 was co-stained with LTL and PNA, respectively. Nuclei were visualized using DAPI staining. Scale bar for SA-β-gal staining, 100 μm; Scale bar for p21 and γH2AX staining, 50 μm; Scale bar for LMNB1 staining, 20 μm. *n* = 5 per group. (**B**) The quantification of SA-β-gal, p21, γH2AX, and LMNB1 staining for (a). (**C**) Kidney p16 and Klotho expression were examined by Western blot. (**D**) Kidney Klotho and PAI-1 expression were examined by qRT-PCR. The results are shown as fold change compared with WT mice without IRI. (**E**) Representative images of SA-β-gal, p21, γH2AX and LMNB1 staining from Adv-Erbin-injected kidneys on day 14 after I/R. LMNB1 was co-stained with LTL and PNA, respectively. Nuclei were visualized using DAPI staining. Scale bar for SA-β-gal staining, 100 μm; Scale bar for p21 and γH2AX staining, 50 μm; Scale bar for LMNB1 staining, 20 μm. *n* = 5 per group. (**F**) The quantification of SA-β-gal, p21, γH2AX, and LMNB1 staining for (**E**). (**G**) Kidney p16 and Klotho expression were examined by Western blot. (**H**) Kidney Klotho and PAI-1 expression were examined by qRT-PCR. Results are presented as the mean ± s.e.m. *P* values were determined using one-way ANOVA with Tukey’s multiple comparison test in (**B**–**D**), and one-way ANOVA with Dunnett’s multiple comparisons test in (**F**–**H**).

Next, we isolated the primary TECs and found a higher proportion of Erbin-deficient tubular cells in G2/M phase than in the control (Appendix Fig. S11; Fig. 4H). This observation was confirmed by the increase of pH3^+^Ki67^+^ tubular cells in the Erbin-deficient kidneys (Appendix Fig. S12). When challenging to IRI, the number of tubular cells in G2/M phase was significantly augmented in the Erbin deficiency kidney in comparison with the littermate control (Appendix Fig. S12). Accordingly, the IRI-induced SA-β-gal activities and senescence markers were greatly augmented in Erbin-deficient mice, whereas the antiaging Klotho was further inhibited (Fig. EV1A–D). In contrast, ectopic expression of Erbin significantly reduced pH3^+^Ki67^+^ tubular cells and tubular SA-β-gal activity, inhibited the increase of senescence markers, and restored Klotho after IRI (Appendix Fig. S12; Fig. EV1F–H).

### The detrimental role of Erbin loss in renal fibrosis is associated with tubular senescence

**Figure EV2:**
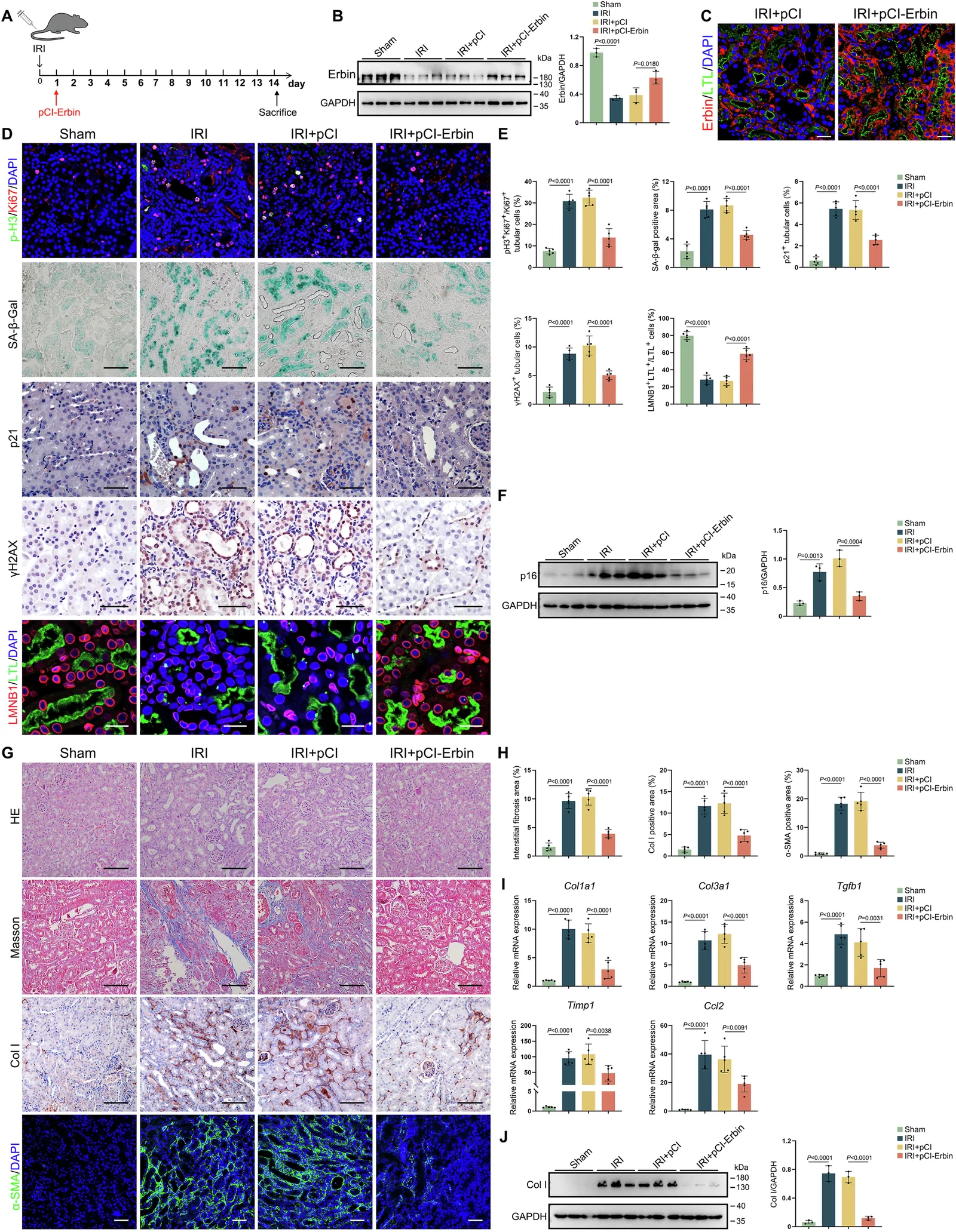
Overexpression of Erbin in the established IRI kidney mitigates tubular senescence and prevents renal fibrosis. (**A**) Schematic diagram of Erbin overexpression after IRI surgery. The pCI-Erbin plasmid was delivered into the kidney 1 day after IRI surgery using the ultrasound microbubble-mediated gene transfer technique. Mice were divided into four groups: Sham, IRI, IRI with delivery of control plasmid (IRI + pCI), and IRI with delivery of pCI-Erbin. Kidneys were harvested at 14 days after I/R (*n* = 5 per group). (**B**) Ebin was examined by Western blot. (**C**) Representative images of cortical kidney sections stained for Erbin and LTL. Nuclei were visualized using DAPI staining. Scale bar, 50 μm. (**D**) Representative images of pH3/Ki67, SA-β-gal, p21, γH2AX, and LMNB1 staining. LMNB1 was co-stained with LTL. Scale bar for SA-β-gal staining, 100 μm; Scale bar for pH3/Ki67, p21, and γH2AX staining, 50 μm; Scale bar for LMNB1 staining, 20 μm. (**E**) Dot plots showing the quantification of pH3/Ki67, SA-β-gal, p21, γH2AX, and LMNB1 staining. (**F**) p16 was examined by Western blot. (**G**) Representative images of HE and Masson staining, and immunohistochemistry for collagen I and immunostaining for α-SMA in mouse kidneys. Scale bars for HE, Masson and Col I IHC, 100 μm; Scale bars for α-SMA immunostaining, 50 μm. (**H**) Quantitative analysis of tubular interstitial fibrosis from Masson staining, and quantification of Col I and α-SMA staining area. (**I**) The mRNA expression of *Col1a1, Col3a1, Tgfb1, Ccl2*, and *Timp 1* were examined by qRT-PCR. The results are shown as fold change compared to WT mice subjected to IRI. (**J**) Kidney collagen I was detected by Western blot. Results are presented as the mean ±  s.e.m. *P* values were determined using one-way ANOVA with Tukey’s multiple comparison test.

The accumulation of senescent TECs promotes progressive fibrosis and impedes adaptive repair after kidney injury (Li et al, 2023; O’Sullivan et al, 2022). To investigate whether the loss of Erbin is involved in renal fibrosis by affecting tubular senescence, we delivered the pCI-Erbin plasmid into the kidney on day 1 after IRI using the noninvasive ultrasound microbubble-mediated plasmid transfer technique (Lan et al, 2003; Li et al, 2019). Ectopic expression of Erbin significantly reduced the number of pH3^+^Ki67^+^ tubular cells in the IRI kidney, and ameliorated tubular senescence (Fig. EV2A–F). Accordingly, the severity of renal fibrosis was markedly alleviated in the pCI-Erbin-delivered kidney (Fig. EV2G–J).

**Figure EV3:**
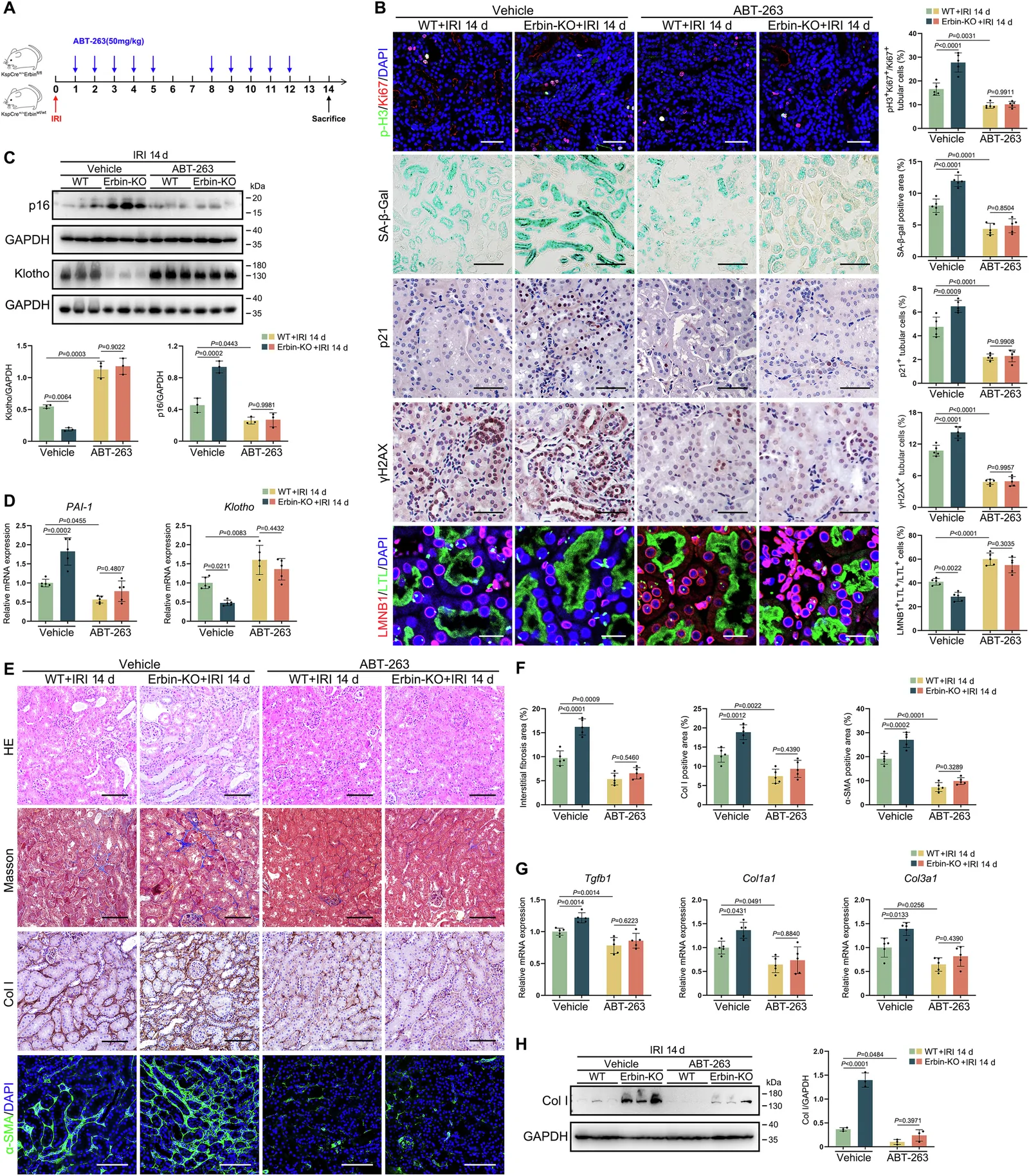
The detrimental effect of Erbin loss on renal fibrosis is associated with tubular senescence. (**A**) Schematic diagram of the IRI model with ABT-263 treatment. ABT-263 was administered by gavage daily for two cycles, each of 4 days, from 1 day after I/R. Kidneys were harvested at 14 days after I/R (*n* = 5 per group). (**B**) Representative images of pH3/Ki67, SA-β-gal, p21, γH2AX, and LMNB1 staining. LMNB1 was co-stained with LTL. Nuclei were visualized using DAPI staining. Scale bar for SA-β-gal staining, 100 μm; Scale bar for pH3/Ki67, p21, and γH2AX staining, 50 μm; Scale bar for LMNB1 staining, 20 μm. *n* = 5 per group. Dot plots showing the quantification of pH3/Ki67, SA-β-gal, p21 and γH2AX staining. (**C**) p16 and Klotho were examined by Western blot. (**D**) PAI-1 and Klotho expression were examined by qRT-PCR. (**E**) Representative images of HE, Masson staining, immunohistochemistry for collagen I, and immunostaining for α-SMA in kidneys. Scale bars, 100 μm. (**F**) Quantitative analysis of tubular interstitial fibrosis from Masson staining, and quantification of Col I and α-SMA staining area. (**G**) The mRNA expression of kidney *Tgfb1*, *Col1a1*, and *Col3a1* were examined by qRT-PCR. The results are shown as fold change compared to the vehicle-treated WT mice. (**H**) Kidney collagen I was detected by Western blot. Results are presented as the mean ± s.e.m. *P* values were determined using one-way ANOVA with Tukey’s multiple comparison test.

ABT-263 is a potent Bcl-2 inhibitor that can specifically and effectively deplete senescent TECs in vivo (Mylonas et al, 2021). Administration of ABT-263 from day 1 after I/R abolished the detrimental effect of Erbin deficiency on tubular senescence, and abrogated the exacerbation of IRI-induced interstitial fibrosis in the Erbin-deficient kidney (Fig. EV3). Meanwhile, ABT-263 treatment inhibited the increase of KIM-1 and NGAL, and improved the deteriorated tubular architecture in the IRI kidney (Appendix Fig. S13).

### Erbin interacts with YB-1 and maintains its stability in tubular cells

**Figure 5:**
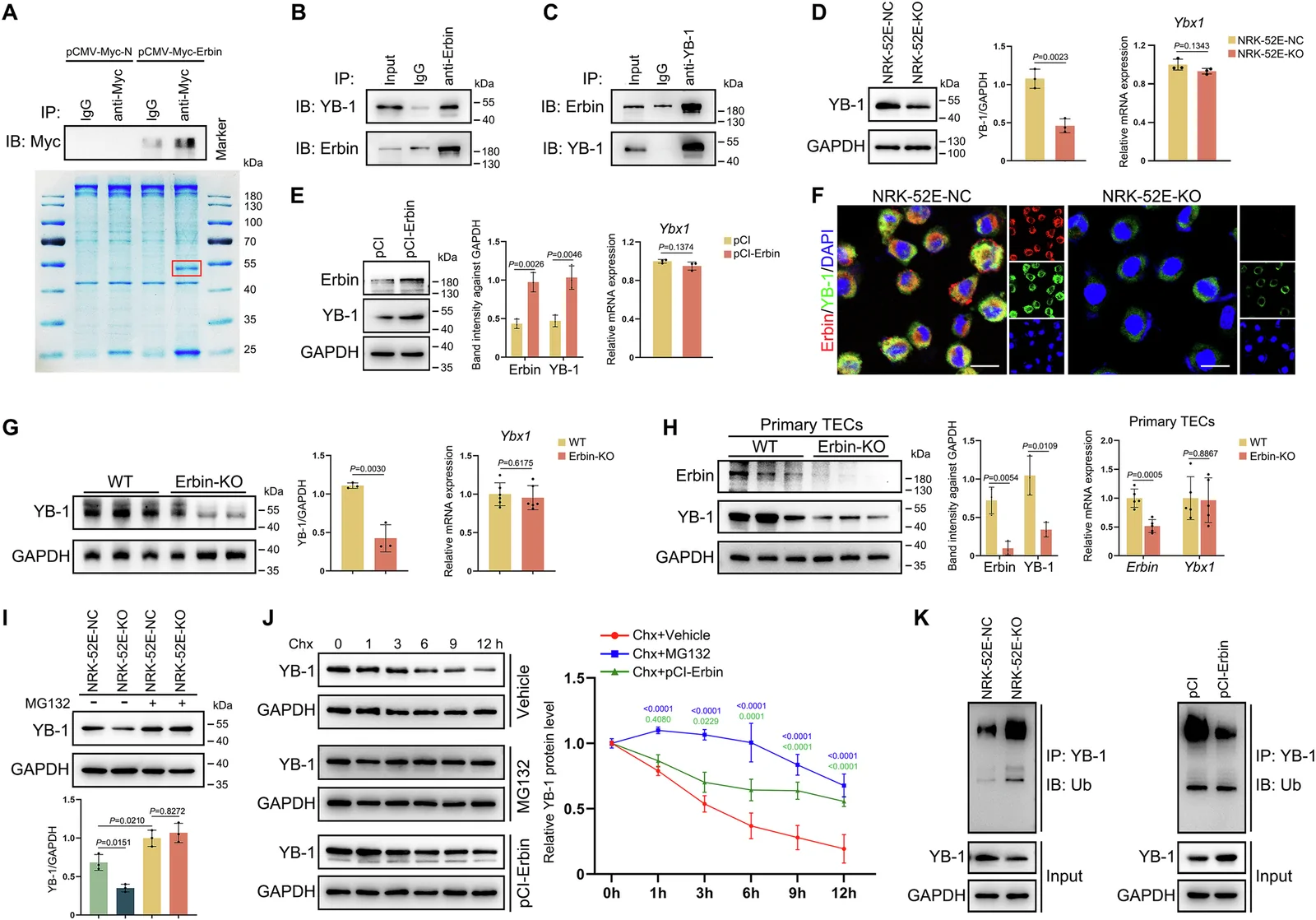
Erbin interacts with YB-1 and maintains its stability in tubular cells. (**A**) NRK-52E cells were transfected with pCMV-Myc-Erbin for 24 h. The cell lysates were subjected to immunoprecipitation using an anti-Myc antibody. The precipitates were immunoblotted with Myc antibody (top), and separated by SDS-PAGE and Coomassie staining (bottom). The red box indicated the enriched band for Mass-spectrometry analysis. (**B**, **C**) NRK-52E cells were subjected to IP assay using Erbin (**B**) or YB-1 (**C**) antibody. The retrieved protein was detected by Western blot. (**D**) Erbin knockout reduced YB-1 protein level in NRK-52E cells (left), but not the mRNA expression (right). (**E**) Overexpression of Erbin increased the amount of YB-1 protein (left), but not the mRNA (right), in NRK-52E cells. (**F**) Representative images of Erbin and YB-1 co-staining of Erbin knockout NRK-52E cells. Nuclei were visualized using DAPI staining. Scale bar, 20 μm. (**G**) Knockout of Erbin greatly decreased YB-1 protein (left), but did not alter the mRNA expression (right) in the kidney (*n* = 5 per group). (**H**) YB-1 was examined by western blot (left) and qRT-PCR (right) in the primary TECs isolated from Erbin knockout mice. (**I**) MG132 restores the YB-1 product in the Erbin knockout NRK-52E cells. (**J**) Erbin stabilizes the YB-1 protein. NRK-52E cells were treated by Chx at 100 μg/ml (top), Chx and MG132 at 20 μM (middle), or transfection with pCI-Erbin plasmid for 24 h and then with Chx (bottom), for the indicated time intervals. The expression of YB-1 at each time-point was detected by western blot and quantified by intensity against GAPDH. The remaining YB-1 protein was expressed as a percentage relative to that at the beginning time. The blue and green asterisks indicate statistically significant differences between MG132 versus Vehicle, and between pCI-Erbin versus Vehicle, respectively. (**K**) Analysis of YB-1-associated ubiquitination in Erbin knockout (left) or Erbin overexpressing (right) NRK-52E cells. The cells were treated with MG132 (20 μM) for 2 h before harvesting and subjected to IP with YB-1 antibody. The precipitates were examined using the antibody against Ubiquitin (Ub) by Western blot. The above experiments were repeated three times. Results are presented as the mean ±  s.e.m. *P* values were determined using two-tailed unpaired Student’s *t*-test in (**D**, **E**, **G**, **H**), one-way ANOVA with Tukey’s multiple comparison test in (**I**), and one-way ANOVA with Dunnett’s multiple comparison test in (**J**). Source data are available online for this figure.

To identify the interactive protein of Erbin in tubular cells, we precipitated the cell lysates from NRK-52E cells overexpressing Myc-tagged Erbin using Myc antibody (Appendix Fig. S14A), and found a specifically enriched band at approximately 50 kDa by SDS-PAGE (Fig. 5A). Mass-spectrometry analysis of the band identified YB-1 as a potential candidate (Dataset EV1). We then validated the special binding of Erbin to YB-1 by reciprocal IP assays using Erbin and YB-1 antibodies, respectively (Fig. 5B,C).

Interestingly, YB-1 protein was significantly decreased in the Erbin knockout NRK-52E cells, but YB-1 mRNA did not alter (Fig. 5D). Reciprocally, overexpressing Erbin increased YB-1 protein, but did not affect the mRNA level (Fig. 5E). Dual immunostaining confirmed the colocalization of Erbin and YB-1, and the effect of Erbin on YB-1 product in NRK-52E cells, and in the tubular epithelium of Erbin-deficient kidney as well (Fig. 5F,G; Appendix Fig. S14B). This phenomenon was then confirmed in the isolated primary TECs (Fig. 5H). In addition, YB-1 protein was further reduced in both IRI- and cisplatin-treated Erbin-deficient kidneys, whereas Erbin overexpression restored YB-1 in the IRI kidneys (Appendix Fig. S14C,D).

There are four ubiquitination sites on YB-1 (Kim et al, 2011). To investigate whether Erbin affects YB-1 stability through proteasome-mediated degradation, we treated the Erbin- deficient NRK-52E cells with proteasome inhibitor MG132, and found a restoration of YB-1 (Fig. 5I). Next, we treated NRK-52E cells with protein synthesis inhibitor cycloheximide (CHX), showing that MG132 significantly increased the half-life of YB-1 protein. In parallel, overexpressing Erbin also markedly halted the turnover of YB-1 (Fig. 5J). Finally, higher levels of YB-1 ubiquitination were observed in the Erbin knockout cells, whereas overexpressing Erbin inhibited YB-1 ubiquitination (Fig. 5K).

### Erbin deletion enhances the LMO7-mediated ubiquitination and degradation of YB-1

**Figure EV4:**
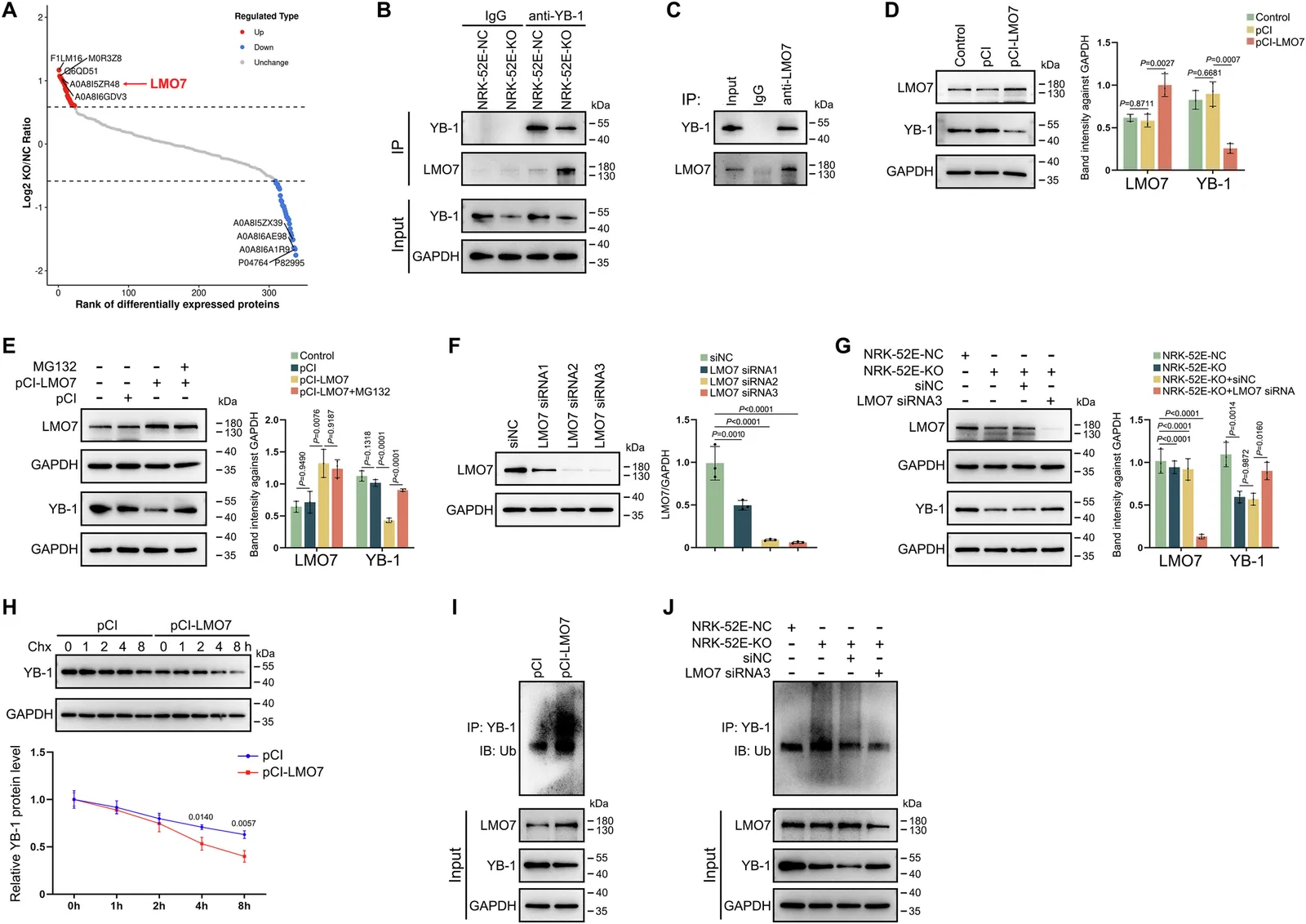
Erbin protects YB-1 from degradation through interfering the LMO7-mediated ubiquitination. (**A**) The change of the YB-1-interacting proteins by Erbin deletion. NRK-52E-KO and the control cells were subjected to IP using an anti-YB-1 antibody, and an MS assay of the precipitates. Red, the significantly enriched YB-1-interacting proteins by Erbin deletion; Blue, significantly decreased proteins by Erbin deletion. The arrow points to LMO7. (**B**) The interaction between YB-1 and LMO7 was tested by IP assay of NRK-52E-KO cells using an anti-YB-1 antibody. The retrieved protein was detected by Western blot. (**C**) NRK-52E cells were subjected to an IP assay using the LMO7 antibody. The retrieved protein was detected by Western blot. (**D**) LMO7 overexpression reduced YB-1 production in NRK-52E cells. NRK-52E cells were transfected with the pCI-LMO7 plasmid for 48 h. (**E**) MG132 abolished the inhibitory effect of LMO7 overexpression on YB-1 in NRK-52E cells. (**F**, **G**) Silencing LMO7 abrogated the inhibitory effect of Erbin deficiency on YB-1 production in NRK-52E cells. (**H**) LMO7 overexpression reduced the half-time of the YB-1 protein. NRK-52E cells were transfected with the pCI-LMO7 plasmid for 24 h, and then treated with 100 μg/mL cycloheximide and harvested at various time points as indicated for western blot. The remaining YB-1 protein was expressed as a percentage relative to that at the beginning time. (**I**) LMO7 overexpression promoted the ubiquitination of YB-1 in NRK-52E cells. (**J**) Silencing LMO7 abrogated the increase of YB-1 ubiquitination in the Erbin-deficient NRK-52E cells. The above experiments were repeated three times. Results are presented as the mean ± s.e.m. *P* values were determined using one-way ANOVA with Dunnett’s multiple comparison test in (**D**, **F**), one-way ANOVA with Tukey’s multiple comparison test in (**E**, **G**), and two-tailed unpaired Student’s *t*-test in (**H**).

To investigate the underlying mechanism by which Erbin affects YB-1 ubiquitination, we performed IP on Erbin-deficient NRK-52E cells using an antibody against YB-1. Mass-spectrometry analysis revealed that 23 proteins were significantly enriched, and 30 proteins were decreased in the immunoprecipitates of Erbin-deficient NRK-52E cells as compared to the control. Notably, LIM domain only 7 (LMO7), an E3 ubiquitin ligase (Sun et al, 2025; Wu et al, 2023), was one of the top five enriched proteins (Fig. EV4A; Dataset EV2). Reciprocal IP using the antibodies against YB-1 and LMO7 revealed an inhibitory role of Erbin in the binding of LMO7 to YB-1 (Fig. EV4B,C).

LMO7 overexpression significantly reduced YB-1 protein level in NRK-52E cells (Fig. EV4D). However, treatment with a proteasome inhibitor (MG132) abrogated the inhibitory effects of LMO7 on YB-1 (Fig. EV4E). In contrast, knocking down LMO7 restored YB-1 product in the Erbin-deficient NRK-52E cells (Fig. EV4F,G). Furthermore, LMO7 overexpression shortened the half-life of YB-1, suggesting that LMO7 accelerated the proteasomal degradation of YB-1. (Fig. EV4H). Consistently, LMO7 overexpression significantly promoted the ubiquitination of YB-1 in NRK-52E cells, whereas knocking down LMO7 suppressed the increase of YB-1 ubiquitination in Erbin-deficient NRK-52E cells (Fig. EV4H,I).

### Erbin protects the tubular cells from senescence by interacting with YB-1

**Figure EV5:**
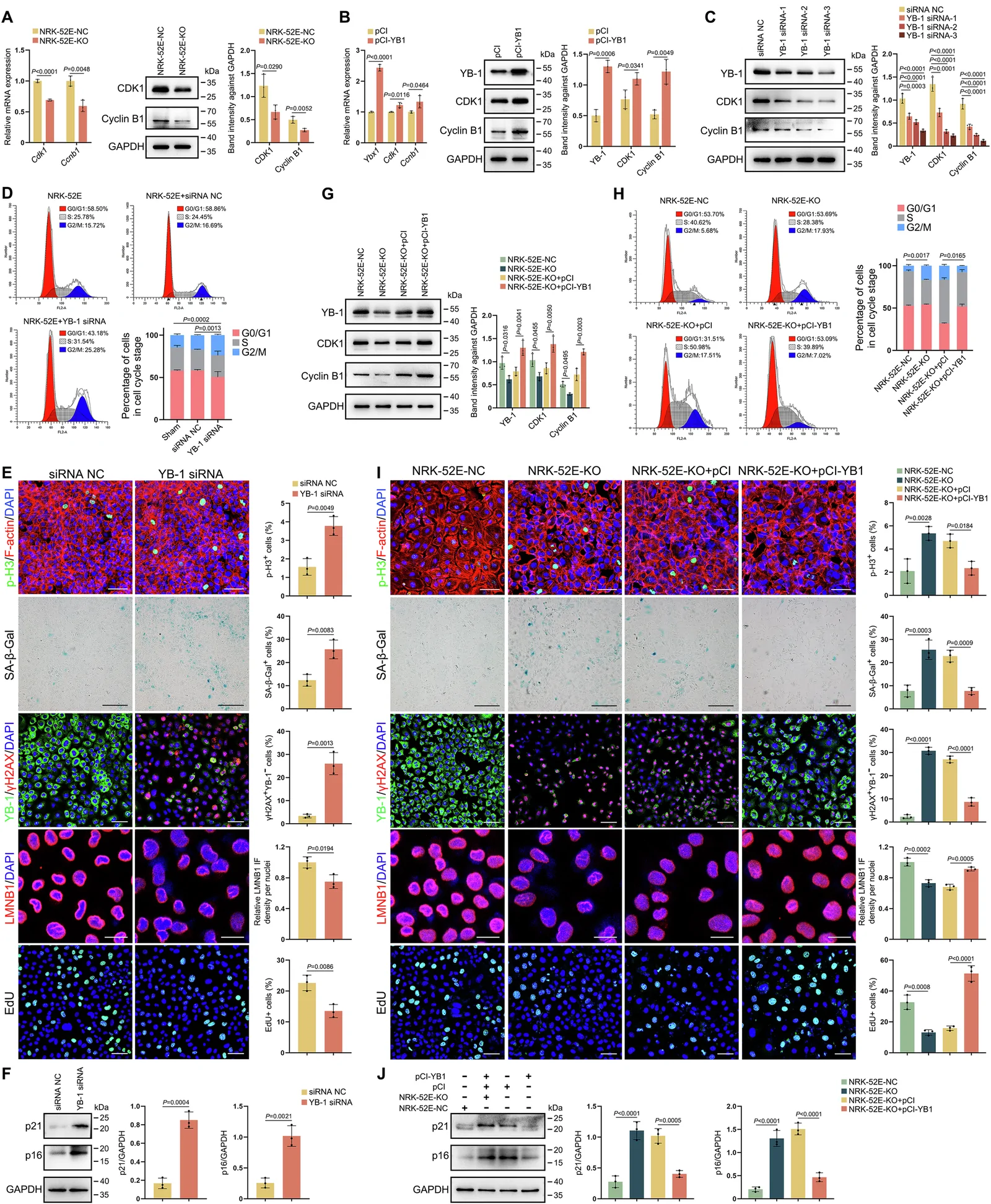
Erbin protects the tubular cells from senescence by interacting with YB-1. (**A**) Deletion of Erbin significantly reduces the expression of CDK1 and Cyclin B1 in NRK-52E cells, as indicated by qRT-PCR (left) and western blot (right). (**B**) Overexpression of YB-1 leads to a significant increase in CDK1 and Cyclin B1 in NRK-52E cells. NRK-52E cells were transfected with the pCI-YB-1 plasmid for 48 h. (**C**) Knockdown of YB-1 greatly reduces CDK1 and Cyclin B1 in NRK-52E cells. NRK-52E cells were transfected with a set of YB-1 siRNA or the control for 48 h. (**D**) Cell cycle analysis by flow cytometry shows that knocking down YB-1 increased the proportion of cells in G2/M phase. (**E**) Representative images of p-H3 immunostaining, SA-β-gal staining, γH2AX /YB-1 dual immunostaining, LMNB1 immunostaining, and EdU staining after knocking down YB-1 in NRK-52E cells. Scale bar for LMNB1 immunostaining, 20 μm. Scale bar for others, 50 μm. The dot plot shows the corresponding quantification of the images, respectively. (**F**) Knockdown of YB-1 increases p21 and p16 in NRK-52E cells. (**G**) Ectopic expression of YB-1 restores CDK1 and Cyclin B1 in Erbin knockout NRK-52E cells. (**H**) Flow cytometry showing that restoring YB-1 reduced the proportion of G2/M phase in Erbin knockout NRK-52E cells. (**I**) Representative images of p-H3 immunostaining, SA-β-gal staining, γH2AX/YB-1 dual immunostaining, LMNB1 immunostaining, and EdU staining for Erbin knockout NRK-52E cells overexpressing YB-1. Scale bar for LMNB1 immunostaining, 20 μm. Scale bar for others, 50 μm. The dot plot shows the corresponding quantification of the images, respectively. The above experiments were repeated three times. (**J**) Ectopic expression of YB-1 inhibits p21 and p16 in Erbin knockout NRK-52E cells. Results are presented as the mean ± s.e.m. *P* values were determined using two-tailed unpaired Student’s *t*-test in (**A**, **B**, **E**, **F**), one-way ANOVA with Dunnett’s multiple comparison test in (**C**), and one-way ANOVA with Tukey’s multiple comparison test in (**D**, **G**, **H**, **I**, **J**).

RNA-seq analysis of the Erbin-deficient NRK-52E cells showed that CDK1 and cyclin B1 were preferentially downregulated among the dysregulated transcripts related to cell cycle regulation (Appendix Fig. S5; Fig. EV5A). Given the critical role of CDK1/cyclin B1 complex in mitosis initiation, as well as the potential of YB-1 in regulating gene expression, we hypothesize that Erbin regulates the cell cycle of TECs by interacting with YB-1, which, in turn, maintains CDK1/cyclin B1 expression (Santamaría et al, 2007; Satyanarayana and Kaldis, 2009; Vigneron et al, 2018; Wang et al, 2022). To this end, we overexpressed YB-1 in NRK-52E cells, and showed a significant upregulation of cyclin B1 and CDK1 (Fig. EV5B). In contrast, knocking down YB-1 greatly reduced CDK1 and cyclin B1, increased the proportion of cells in G2/M phase, and promoted p21 and p16 expression, and the senescence of NRK-52E cells (Fig. EV5C–F). Moreover, ectopic expression of YB-1 restored CDK1 and cyclin B1 in Erbin-deficient NRK-52E cells, and, as a consequence, reduced the cells in G2/M phase, and inhibited the increase of p21 and p16, and the induction of cellular senescence (Fig. EV5G–J). Finally, overexpressing YB-1, or Erbin, in the H_2_O_2_-treated NRK-52E cells restored CDK1 and cyclin B1, and protected against the H_2_O_2_-induced tubular cell senescence (Appendix Figs. S16 and S17).

### The role of tubular Erbin in AKI and CKD depends on YB-1

**Figure 6:**
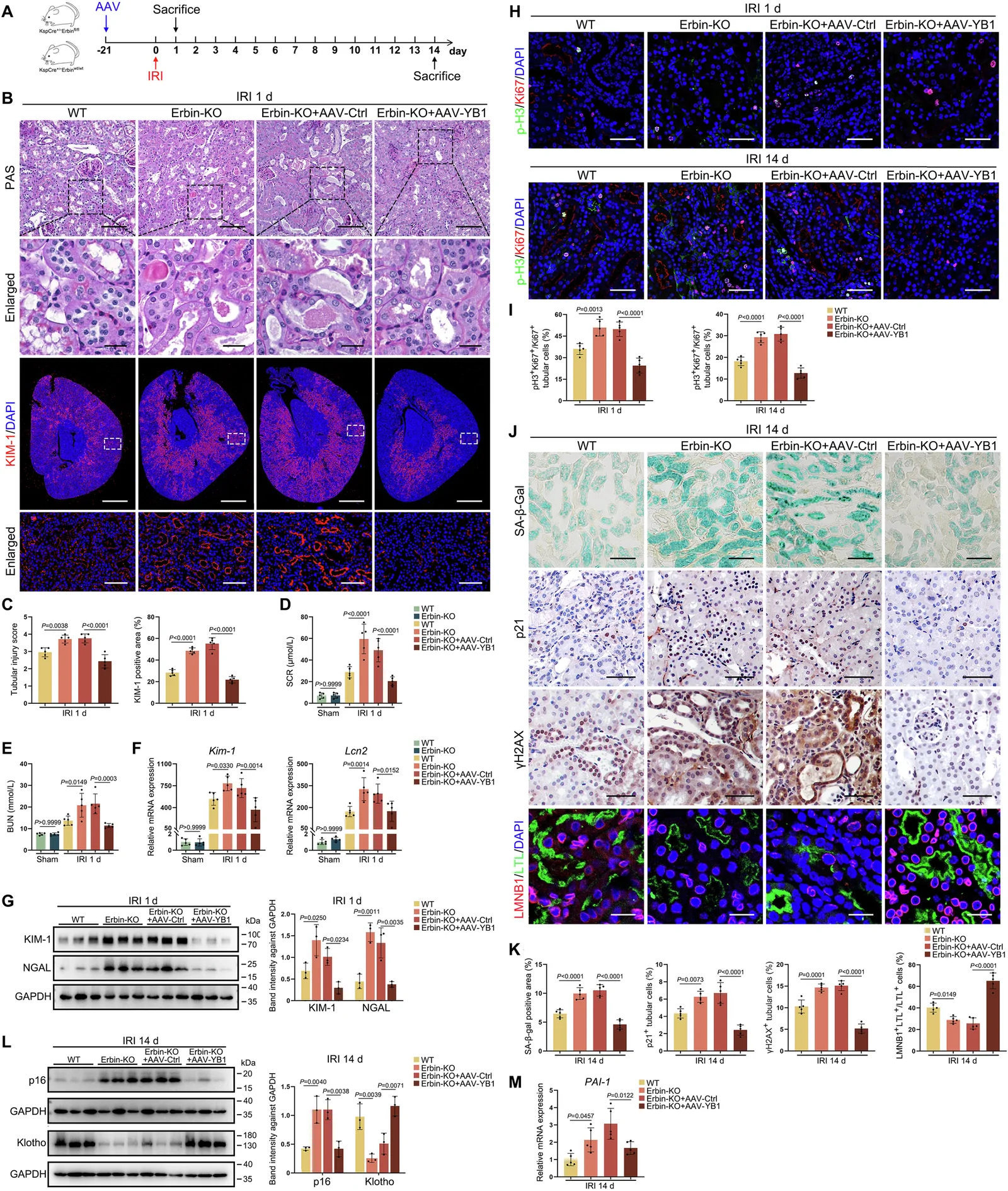
The role of tubular Erbin in AKI and CKD depends on YB-1. (**A**) Schematic diagram of the IRI model using Erbin-deficient mice with ectopic expression of YB-1. The adeno-associated virus (AAV) was administered in situ in the cortex of the kidney 21 days before I/R. Kidneys were harvested at day 1 or 14 after I/R (*n* = 5 per group). (**B**) Representative images of PAS staining and KIM-1 immunostaining for kidneys at one day after I/R. Scale bar for PAS staining, 100 μm, and the boxed areas are enlarged (Scale bar, 25 μm). For KIM-1 immunostaining, the images of the whole kidney cross-sectional area are stitched at ×200 magnification (Scale bar, 1000 μm), and the bottom panels are enlarged from the boxed areas (Scale bar, 100 μm). (**C**) Tubular injury score and quantification of KIM-1 staining in cortex and outer medulla from whole kidney section images. (**D**, **E**) SCR and BUN levels at 1 day after I/R. (**F**, **G**) KIM-1 and NGAL expression were examined by qRT-PCR (**F**) and western blot (**G**) at 1 day after I/R. (**H**) Representative images of pH3/Ki67 dual immunostaining from kidneys at 1 day, and 14 days after I/R. Scale bar for pH3/Ki67 staining, 50 μm. (**I**) The percentage of pH3^+^Ki67^+^ tubular cells relative to the proliferating Ki67^+^ tubular cells in different groups. (**J**) Representative images of SA-β-gal, p21, γH2AX, and LMNB1 staining from kidneys at 14 days after I/R. LMNB1 was co-stained with LTL. Nuclei were visualized using DAPI staining. Scale bar for SA-β-gal staining, 100 μm; Scale bar for p21 and γH2AX staining, 50 μm; Scale bar for LMNB1 staining, 20 μm. (**K**) The quantification of SA-β-gal, p21, γH2AX, and LMNB1 staining for (**J**). (**L**) p16 and Klotho expression were examined by Western blot. (**M**) The PAI-1mRNA was examined by qRT-PCR. The results are shown as fold change compared to WT mice subjected to IRI. Results are presented as the mean ± s.e.m. *P* values were determined using one-way ANOVA with Tukey’s multiple comparison test. Source data are available online for this figure.

To confirm the involvement of YB-1 in the pathogenesis of IRI in Erbin-deficient mice, we restored YB-1 expression in the kidney by in situ injecting the adeno-associated virus serotype 9 carrying YB-1 (Referred to as AAV-YB-1) three weeks before I/R (Fig. 6A). We evaluated the efficiency of AAV infection and confirmed the YB-1 overexpression in tubular cells (Appendix Fig. S18). Ectopic expression of YB-1 abrogated the detrimental effect of Erbin deficiency on AKI, restored renal function, and prevented the increase of KIM-1 and NGAL in the IRI kidneys of Erbin-KO mice (Fig. 6B–G; Appendix Fig. S19). The proportion of pH3^+^Ki67^+^ tubular cells, tubular SA-β-gal activity, and senescence markers were significantly inhibited in the AAV-YB-1-treated kidney on day 14 after I/R, whereas Klotho was restored (Fig. 6H–M). Meanwhile, overexpression of YB-1 abolished the detrimental effect of Erbin deficiency on IRI-induced interstitial fibrosis (Appendix Fig. S20).

### The loss of Erbin in TECs accelerates kidney aging

**Figure 7:**
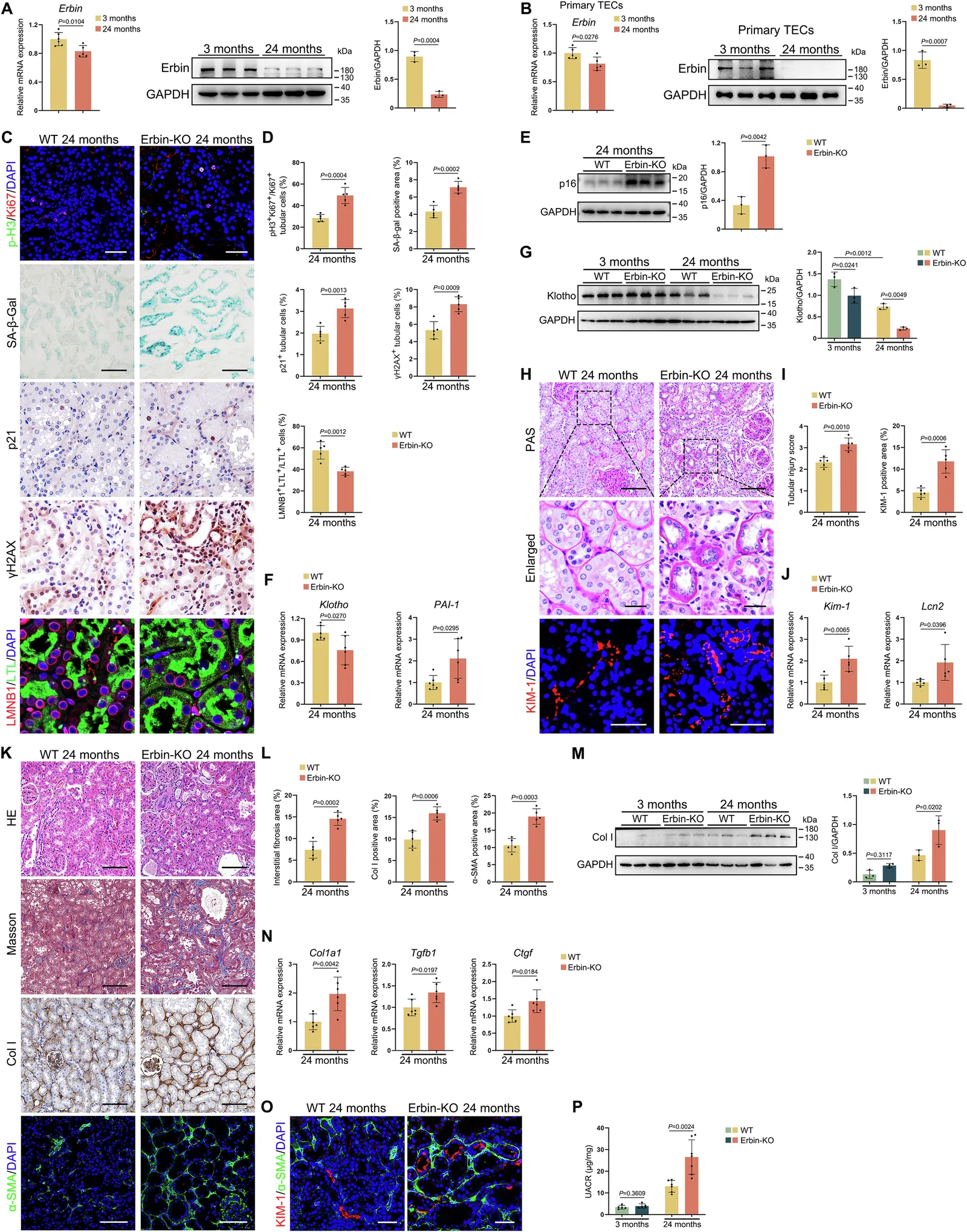
The loss of Erbin in tubular cells contributes to kidney aging in mice. (**A**) Erbin is significantly decreased in the aged kidney as indicated by qRT-PCR and western blot (*n* = 5 per group). (**B**) Erbin is significantly decreased in the primary TECs isolated from the aged kidney. (**C**) Representative images of pH3/Ki67 dual immunostaining and SA-β-gal, p21, γH2AX, and LMNB1 staining from kidneys of WT and Erbin-KO mice at the age of 24 months (*n* = 5 per group). LMNB1 was co-stained with LTL. Scale bar for pH3/Ki67, p21, and γH2AX staining, 50 μm; Scale bar for SA-β-gal staining, 100 μm; Scale bar for LMNB1 staining, 20 μm. (**D**) The quantification assay for (**C**). (**E**) p16 was examined by Western blot. (**F**) PAI-1 and Klotho mRNA expression in the aged kidney was examined by qRT-PCR. The results are shown as fold change relative to the 24-month-old WT mice. (**G**) Klotho expression was examined by Western blot. (**H**) Representative images of PAS staining and KIM-1 immunostaining showing exacerbated epithelial damage in Erbin-deficient mice at 24 months old (*n* = 5 per group). Scale bar for PAS staining, 100 μm. Boxed areas are enlarged (Scale bar, 25 μm). Scale bar for KIM-1 staining, 50 μm. (**I**) Tubular injury score and quantification of KIM-1 staining. (**J**) KIM-1 and NGAL expression were examined by qRT-PCR. (**K**) Representative images of HE, Masson staining, immunohistochemistry for collagen I, and immunostaining for α-SMA in the aged kidneys (*n* = 5 per group). Scale bars, 100 μm. (**L**) Quantitative analysis of tubular interstitial fibrosis from Masson staining, and quantification of Col I and α-SMA staining area. (**M**) Kidney collagen I was detected by Western blot. (**N**) The mRNA expression of kidney *Col1a1, Tgfb1*, and *Ctgf* were examined by qRT-PCR. The results are shown as fold change relative to the 24-month-old WT mice. (**O**) Representative images of KIM-1 and α-SMA co-staining from kidneys of WT and Erbin-KO mice at the age of 24 months. Scale bars, 50 μm. (**P**) Urinary albumin to urinary creatinine ratios (UACR) in Erbin-deficient and WT mice at the age of 3 and 24 months (*n* = 5 for 3-month-old WT and Erbin-KO mice, *n* = 6 for 24-month-old WT mice, and *n* = 7 for 24-month-old Erbin-KO mice). Results are presented as the mean ± s.e.m. *P* values were determined using two-tailed unpaired Student’s *t*-test in (**A**, **B**, **D**, **E**, **I**, **J**, **L**, **N**, **P**), two-tailed Student’s with Welch’s correction in (**F**), one-way ANOVA with Tukey’s multiple comparison test in (**G**, **M**). Source data are available online for this figure.

We found a significant decrease of Erbin in the aged kidneys, and confirmed this observation in the primary TECs (Fig. 7A,B; Appendix Fig. S21A–D). When the primary TECs from young kidneys were treated with D-galactose, which has been widely used to induce renal aging (Wang et al, 2024), Erbin was also significantly decreased (Appendix Fig. S21E). Hence, we compared the naturally aged kidneys at the same age, and found a dramatic increase of pH3^+^Ki67^+^ tubular cells, tubular SA-β-gal activity and senescence markers in the Erbin-deficient mice (Fig. 7C–G). Moreover, the primary TECs from the aged kidneys of Erbin knockout mice showed a higher proportion of CDH16^+^ tubular cells in G2/M phase in comparison with WT littermates, suggesting a causative role of Erbin loss in promoting tubular cell senescence (Appendix Fig. S21F,G).

Although tubule-specific deletion of Erbin did not affect kidney structure at the age of 3 months (Fig. 2A), tubular damage, as well as KIM-1 and NGAL expression, was significantly augmented at 24-month-old as compared to the WT mice (Fig. 7H–J). Moreover, deteriorated interstitial fibrosis and higher expression of profibrotic factors were observed in the aged kidneys of Erbin-deficient mice (Fig. 7K–N). Of note, greater amounts of α-SMA-positive myofibroblasts appeared around the KIM-1-expressing tubules in the aged kidneys of Erbin-deficient mice, suggesting a detrimental cross-talk between senescent tubular cells and interstitial fibroblasts (Fig. 7O).

The SCR and BUN levels were comparable between Erbin-deficient and WT mice at the age of 24 months, but urinary albumin concentrations, urinary creatinine, and urinary albumin to urinary creatinine ratios (UACR) were much higher in the Erbin knockout mice (Fig. 7P; Appendix Fig. S21H). It is also worth noting that the lifespan of Erbin-deficient mice are affected (Appendix Fig. S21I), thereby suggesting a causative role of tubular Erbin loss for the pathological consequences of aging.

### The loss of Erbin is associated with tubular senescence in human CKD samples

**Figure 8:**
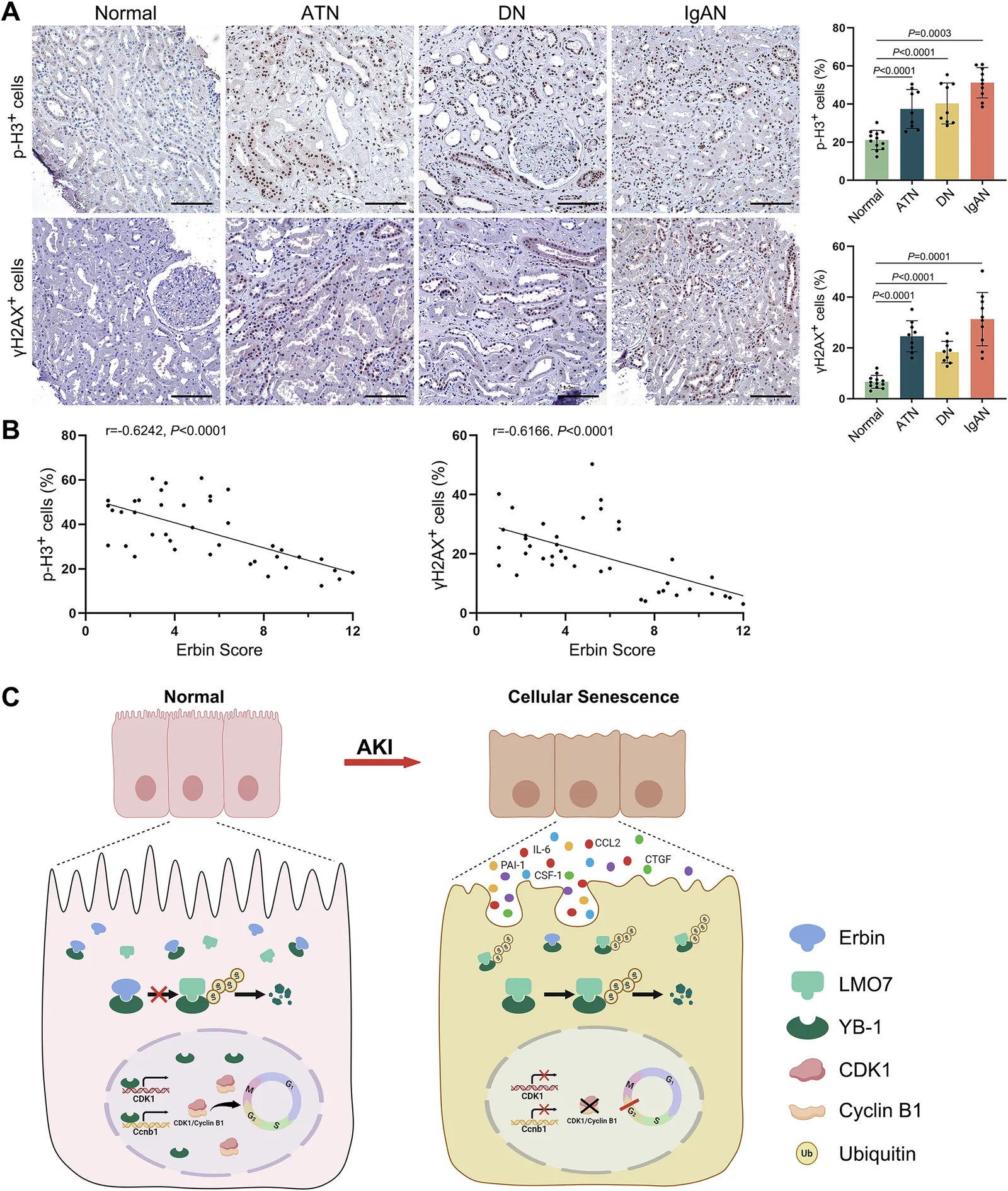
The loss of Erbin is associated with tubular senescence in human CKD samples. (**A**) Representative images of p-H3 and γH2AX immunostaining of kidney biopsies from ATN (*n* = 10), DN (*n* = 9), IgAN (*n* = 9) patients, and normal controls (*n* = 12). Scale bar, 100 μm. Dot plots show the quantitative assay for p-H3 and γH2AX immunostaining of kidney biopsies. (**B**) Erbin expression is negatively correlated with the percentage of p-H3^+^ tubular cells, and γH2AX^+^ tubular cells as well, in the specimens from CKD patients (*n* = 40). The Spearman correlation coefficient (r) is shown. (**C**) Schematic diagram showing that loss of Erbin increases the proportion of G2/M phase, and facilitates the onset of senescence in tubular cells. Results are presented as the mean ± s.e.m. *P* values were determined using Brown–Forsythe and Welch ANOVA with Tamhane’s multiple comparisons test in (**A**). Source data are available online for this figure.

We stained serial sections of human kidney biopsies with different types of etiology for p-H3 and γH2AX. The number of p-H3-positive, as well as γH2AX-positive tubular cells was greatly increased in the biopsies from CKD patients as compared to those from normal control (Fig. 8A). Moreover, there was a negative linear correlation between tubular Erbin content and the number of p-H3-positive, as well as γH2AX-positive, tubular cells in the specimens of CKD patients, thus suggesting that the loss of Erbin is clinically implicated with the regulation of tubular cell cycle and senescence in the progression of CKD (Fig. 8B).

## Discussion

Erbin is widely expressed in the epithelia of most human and mouse tissues, including the liver, kidney, spleen, and intestine (Borg et al, 2000). The loss of Erbin has been previously identified in different pathophysiological conditions (Rachmin et al, 2014; Shen et al, 2018). For instance, Erbin is highly expressed in the intestinal mucosa, but it is significantly downregulated in the murine model of inflammatory bowel disease (IBD) and patients with ulcerative colitis. Accordingly, Erbin deficiency greatly exacerbated the autophagic cell death and the severity of IBD in mice (Shen et al, 2018). Of note, Erbin is significantly decreased in the HER2-overexpressing breast cancer tissues (Liu et al, 2013). Interestingly, we have previously reported that HER2 is increased in the proximal tubular epithelial cells in the murine CKD models (Li et al, 2019). Thus, whether and how HER2 is implicated in the regulation of Erbin might provide us a clue to explore the regulatory mechanism of Erbin expression in the injured tubular cells. In addition, using the cultured cell lines, including the human kidney cell line 293T, a previous study showed that Erbin expression exhibits a feature of cell cycle periodicity and peaks in G2/M phase. Mechanistically, c-Myb promotes the cell cycle-dependent transcriptional activation of Erbin by binding to the core region of the Erbin promoter (Liu et al, 2012b). These results unravel a critical role of c-Myb in Erbin transcription in G2/M phase and also predict a possible role of Erbin in cell cycle progression.

The pathogenic role of Erbin has been attributed to the ability of modulating signaling transduction or influencing the ubiquitination and stability of the interacting protein. For instance, Erbin inhibits estrogen receptor-α (ERα) expression in hepatocellular carcinoma cells by directly binding to ERα, enhancing the interaction between ERα and E3 ubiquitin ligase Chip, and eventually facilitating ERα ubiquitination (Wu et al, 2017). Nevertheless, in breast cancer cells, the binding of Erbin to ErbB2 prevents the degradation of ErbB2 by interfering Chip-mediated ubiquitination (Tao et al, 2014). In colorectal cancer, Erbin inhibits the ubiquitination of EGFR by interacting with the E3 ubiquitin ligase c-Cbl (Yao et al, 2015). Herein, we found that Erbin interacted with YB-1 and prevented its ubiquitination and degradation by interfering with the binding of YB-1 to LMO7, an E3 ubiquitin ligase belonging to the PDZ and LIM domain-containing protein family (Sun et al, 2025; Wu et al, 2023). The presence of homological PDZ, a well-known protein interaction domain, in both Erbin and LMO7, therefore, suggests a regulatory mechanism of Erbin on YB-1 ubiquitination by competitively inhibiting the binding of LMO7 to YB-1.

In healthy tissue, YB-1 is predominantly cytoplasmic and influences the translation of target mRNAs (El-Naggar et al, 2015; Evdokimova et al, 2009). Meanwhile, YB-1 is also a well-characterized transcription factor involved in the control of cell division (Zhang et al, 2020). Due to the potential of ubiquitination, the multiple functions of YB-1 are related to its intracellular stability (Kim et al, 2011). For instance, activated protein C protects against IRI-induced tubular injury by preserving the expression of deubiquitinating enzyme otubain-1, and thus restricting YB-1 ubiquitination in tubular cells (Dong et al, 2015). In line with our observation, a previous study showed that Hnscr, a long non-coding RNA, inhibits the senescence of neural stem cells by binding to YB-1 and preventing its ubiquitination (Xiao et al, 2020). Thus, YB-1 ubiquitination and degradation exhibit potential roles in cellular senescence, and might be controlled by individual mechanisms depending on the specific cell types and contexts.

Depending on the inducer and cell type, cellular senescence is regulated by various mechanisms converging on the pathways that eventually halt cell cycle by increasing the cyclin kinase inhibitors such as p21, p16 and p53, or inhibiting the cell-cycle activities mediated by the cyclin and CDK complexes (Satyanarayana and Kaldis, 2009). The association of cyclin B1 with CDK1 is required for CDK1 activity. Thus, the dysregulation of cyclin B1 is supposed to induce cell cycle arrest and the onset of senescence. For instance, macrophage migration inhibitory factor (MIF) reduced the proportion of tubular cells in G2/M phase by maintaining cyclin B1 expression in vitro and in vivo (Djudjaj et al, 2017). The involvement of CDKs in cellular senescence has also been reported in various disease conditions. Inhibition of CDK4 and CDK6 leads to a prolongation of G1/S phase, and induces both cancer cells and non-malignant cells to enter a p53-dependent senescent state (Crozier et al, 2022; Goel et al, 2016; Wang et al, 2022). In comparison with other CDKs, CDK1 is best known for its role in driving G2 phase progression and entry of cells into mitosis (Santamaría et al, 2007; Vigneron et al, 2018). Knocking down CDK1 significantly increases the proportion of G2/M phase in tubular cells (Wang et al, 2022).

YB-1 appears to protect mice from acute and chronic kidney injury in a cell-specific manner, but whether and how YB-1 is involved in tubular cell senescence remains unknown (Dong et al, 2015; Hermert et al, 2020; Rana et al, 2024; Wang et al, 2016). A previous study showed that YB-1 promotes the transcription of p16 in neural stem cells (Xiao et al, 2020). Here, we found that YB-1 is responsible for the Erbin-mediated dysregulation of CDK1 and cyclin B1 in TECs. In line with our observations, YB-1 promotes the transcription of cyclin B1 by specifically binding to the promoter in HeLa cells (Jurchott et al, 2003). In addition, YB-1 recruits RNA helicase DDX6 and translation initiation factor EIF4E to CDK1 mRNA and promotes its translation (Wang et al, 2015). Nevertheless, the underlying mechanism, by which YB-1 maintains the expression of CDK1 and cyclin B1 in TECs, still needs to be further investigated in the future. Interestingly, a recent study showed that YB-1 suppresses the senescence of epidermal progenitor cells by translationally repressing cytokines involved in the onset of senescence (Kwon et al, 2018). Therefore, while our data indicate that YB-1 is responsible for CDK1 and cyclin B1 expression in TECs, we can’t exclude the involvement of other YB-1-regulated targets in tubular cell senescence. Moreover, alternative mechanisms might be implicated, though our results suggest that loss of Erbin prolongs the G2/M phase in tubular cells by destabilizing YB-1. For instance, a recent study showed that Erbin protected tubular cells form pyroptosis via regulating the NLRP3/caspase‑1 pathway in sepsis‑induced acute kidney injury (Liu et al, 2023). In addition, the enrichment of the GO term “regulation of cell death” among upregulated DEGs in Erbin-deficient tubular cells also suggests the possible involvement of Erbin loss in alternative forms of tubular cell death in the progression of kidney injury.

Cellular senescence has been observed in various cell types in the progression of AKI and CKD, but the senescence of TECs correlates more strongly with the severity and renal function loss than others in human biopsies (Liu et al, 2012a; Sis et al, 2007). In addition, the accumulation of senescent TECs is more severe than in other cell types in murine models of aging (Baker et al, 2016). In the present study, tubule-specific deletion of Erbin is supposed to accelerate tubular cell senescence, impair the integrity of tubular epithelium and potentially exacerbate the decline of functional nephrons number in the aging kidney, as indicated by the increase of UACR. These structural and functional changes might ultimately affect the lifespan of the aged mice.

A previous study has shown that removing senescent tubular cells results in attenuation of renal aging or alleviation of CKD (Mylonas et al, 2021). However, the contribution of cellular senescence to kidney injury may vary at different pathological stages. For instance, p21 knockout mice were more susceptible to IRI-induced acute renal failure, though increased cell cycle activity was identified in the kidney cells (Megyesi et al, 2001). Similarly, early elimination of p16-overexpressing cells failed to improve kidney function and structure in a renal artery stenosis (RAS)-induced chronic ischemia model, while later senolytic treatment after RAS induction was successful (Kim et al, 2021). These observations suggest that cellular senescence may be initially protective, but ultimately drives persistent ischemic injury. A recent study showed that inhibiting poly (ADP-ribose) polymerase 1 (PARP1), an important molecular mediator of the decision between senescence and death, promoted the conversion of cell death into senescence. Notably, pretreatment with a PARP inhibitor led to an increase in transient senescence and improved recovery from IRI-induced kidney injury, suggesting that transient senescence may therapeutically benefit tissue regeneration (Nehme et al, 2024). Thus, the choosing and timing of senoinducing or senolytic therapies should be carefully considered when manipulating senescence for the purpose of preventing the onset or progression of CKD.

In conclusion, our data support a model where the loss of Erbin in the tubular cells under physiological and pathophysiological conditions leads to inappropriate cellular senescence associated with aggravated CKD or acceleration of kidney aging (Fig. 8c). Thus, restoring the Erbin/YB-1 axis potentially offers a benefit for slowing kidney aging and reducing the incidence of CKD.

## Methods

**Table:** Reagents and tools table

Reagent/resource	Reference or source	Identifier or catalog number
**Experimental models**		
NRK-52E (*Rattus norvegicus*)	American Type Culture Collection	CRL-1571
293 T (*Homo sapiens*)	Cell Bank of the Chinese Academy of Sciences	SCSP-502
C57BL/6J (*M. musculus*)	Beijing Vital River Laboratory Animal Technology Co., Ltd. (Beijing, China)	219
Erbin floxed (*M. musculus*)	GemPharmatech Co, Ltd. (Nanjing, China)	T008906
Cdh16-Cre (*M. musculus*)	GemPharmatech Co, Ltd. (Nanjing, China)	T004820
mG/mT reporter (*M. musculus*)	GemPharmatech Co, Ltd. (Nanjing, China)	T006163
**Recombinant DNA**		
pCMV-Myc	Addgene	Cat# 631604
pCI	Promega	Cat# E1731
lentiCas9-Blast	Addgene	Cat# 52962
LentiGuide-Puro	Addgene	Cat# 52963
psPAX2	Addgene	Cat #12260
pMD2.G	Addgene	Cat #12259
**Antibodies**		
α-SMA	BOSTER Biotech	BM0002
Collagen I	Abcam	Ab270993
NGAL	R&D Systems	AF1857
KIM-1	R&D Systems	AF1817
Erbin	Bethyl	A303-762A
Erbin	Abcam	Ab247081
Erbin	Santa Cruz Biotechnology	sc-293468
CDK1	Cell signaling technology	#9116
Cyclin B1	Cell signaling technology	#12231
YB-1	Abcam	Ab76149
AQP2	Abcam	Ab199975
UMOD	Abcam	Ab207170
p-H3	Abcam	Ab14955
γH2AX	Abcam	Ab26350
p16	Santa Cruz	sc-1661
p21	Abcam	Ab188224
LMNB1	Abcam	Ab229025
LMO7	Santa Cruz	sc-376807
Ki67	Abcam	Ab15580
Klotho	R&D Systems	AF1819
EGFP	Abcam	Ab13970
Myc tag	Cell signaling technology	#2278 s
Cytokeratin18	Abcam	Ab315096
E-cadherin	Abcam	Ab231303
CDH16	Abcam	Ab270263
CDH16(Alexa Fluor® 647)	Novus Biologicals	NBP2-47745AF647
Ubiquitin	Cell signaling technology	#3936
GAPDH	Proteintech	HRP-60004
HRP-conjugated anti-mouse IgG	Jackson ImmunoResearch	115-035-003
HRP-conjugated anti-rabbit IgG	Jackson ImmunoResearch	111-035-003
HRP-conjugated anti-goat IgG	Jackson ImmunoResearch	705-035-003
Donkey anti-mouse IgG, Alexa Fluor 488	Thermo Fisher Scientific	A21202
Donkey anti-mouse IgG, Alexa Fluor 546	Thermo Fisher Scientific	A10036
Donkey anti-rabbit IgG, Alexa Fluor 488	Thermo Fisher Scientific	A21206
Donkey anti-rabbit IgG, Alexa Fluor 546	Thermo Fisher Scientific	A10040
Donkey anti-goat IgG, Alexa Fluor 546	Thermo Fisher Scientific	A11056
**Oligonucleotides and other sequence-based reagents**		
siRNA sequences	This study	Table EV1
qPCR primers	This study	Table EV1
guide RNA sequences	This study	Table EV1
PCR and genotyping primers	This study	Methods
**Chemicals, enzymes and other reagents**		
Dulbecco’s Modified Eagle Medium (DMEM)	Invitrogen	11965092
Fetal bovine serum (FBS)	Gbico	A5256701
Trypsin-EDTA	Thermo Fisher	25200072
mineral oil	Byeotime	ST275
H_2_O_2_	Sigma-Aldrich	18304
Cisplatin	MedChem Express	HY-17394
Lipofectamine 2000	Invitrogen	11668500
Blasticidin S	MedChem Express	HY-103401
Puromycin	MedChem Express	HY-B1743
T7 Endonuclease I	New England Biolabs	M0302S
Dulbecco’s Modified Eagle Medium/Nutrient Mixture F12 (DMEM/F12)	Invitrogen	11320033
Collagenase II	Sigma-Aldrich	C2-28
RNase A	Invitrogen	EN0531
Propidium iodide	Invitrogen	00-6990-50
Phosphate Buffered Saline (PBS)	Invitrogen	70011044
Bovine Serum Albumin (BSA)	Sigma-Aldrich	A2153
EDTA	MedChemExpress	HY-W018746
DAPI	Invitrogen	D1306
ABT-263	GLPbio	GC12405
SonoVue	Bracco	
Dimethyl sulfoxide (DMSO)	MedChemExpress	HY-Y0320C
polyethylene glycol 400	MedChemExpress	HY-Y0873A
Tween 80	MedChemExpress	HY-Y1891
Tween 20	MedChemExpress	HY-141415
TRIzol Reagent	Invitrogen	15596026CN
PrimeScript™ RT reagent Kit	Takara	RR047Q
TB Green ® Premix Ex Taq™ II (Tli RNase H Plus) kit	Takara	RR820L
Pierce BCA Protein Assay Kit	Thermo Fisher	23227
PMSF	Thermo Fisher	36978
One-Step PAGE Gel Fast Preparation Kit	Vazyme	E303-01
PageRuler™ Prestained Protein Ladder	Thermo Fisher	26616
PVDF membranes	Millipore	IPVH00005
SignalFire™ Elite ECL Reagent	Cell Signaling Technology	#12757
Paraformaldehyde (PFA)	Thermo Fisher	28906
DyLight 594 Phalloidin	Cell Signalling Technology	#12877
Triton X-100	Sigma-Aldrich	X100
Tris Buffered Saline	Cell Signaling Technology	#12498
Lotus Tetragonolobus Lectin (LTL)	Vector Laboratories	FL-1321-2
Peanut Agglutinin (PNA)	Vector Laboratories	FL-1071-5
MG132	MedChem Express	HY-13259
Cycloheximide (CHX)	MedChem Express	HY-12320
**Software**		
Graphpad Prism 8	https://www.graphpad.com/	
Modifit	https://www.bdbiosciences.com/	
FlowJo V10	https://www.flowjo.com/	
Image J	http://fiji.sc/	
MaxQuant (v. 1.6.7.0)	https://maxquant.org/	
Leica LAS X	https://www.leica-microsystems.com/	
**Other**		
Cell Counting kit-8	Byeotime	C0038
DAB substrate kit	Abcam	Ab64238
Pierce Classic Magnetic IP/Co-IP Kit	Thermo Fisher	88804
Hematoxylin and Eosin (HE) Staining kit	Byeotime	C0105S
Periodic acid schiff (PAS) Staining kit	Nanjing Jiancheng Bioengineering Institute	D004-1-2
Masson’s trichrome Staining kit	ScyTek	TRM-2
Senescence β-Galactosidase Staining Kit	Byeotime	C0602
EdU cell proliferation assay Kit	Byeotime	C0071S
Creatinine Assay kit	Nanjing Jiancheng Bioengineering Institute	C011-2-1
BUN Assay Kit	Nanjing Jiancheng Bioengineering Institute	C013-2-1
Urinary albumin ELISA Quantitation kit	Elabscience	E-EL-M0792
BD FACScalibur flow cytometer	BD Biosciences	
GentleMACS Dissociator	Miltenyi Biotec	
Attune NxT flow cytometer	Thermo Fisher	
double-channel Microplate Reader	Tecan Group Ltd.	
ZEISS LSM980 laser scanning confocal microscope	ZEISS	
StepOne™ Real-Time PCR System	Thermo Fisher	
Nikon DS-Ri2	Nikon	
Leica TCS SP8-MP confocal microscope	Leica	

### Animal experiments

Wild-type C57BL/6J mice were procured from Beijing Vital River Laboratory Animal Technology Co., Ltd. (Beijing, China). Male mice were exclusively used for all experiments in this study. Anesthesia was induced by intraperitoneally injecting with 1.25% tribromoethanol at a dose of 20 μl/g body weight. Ischemic acute kidney injury was induced by clamping bilateral renal arteries for 30 min. During the ischemic period, body temperature was maintained between 37 and 38 °C. After removing the clamps, reperfusion of the kidneys was visually confirmed. The sham group was handled with exposure of the kidneys, but without the operation of ischemia. Cisplatin-induced kidney injury was prepared by a one-time intraperitoneal injection of cisplatin at a dose of 10 mg/kg body weight. The normal control mice were administered the same amount of saline. Animal experiment protocols were reviewed and approved by the Animal Care and Use Committee of Nanjing University (no. IACUC-IACUC-2008007-4) and conformed to the Guidelines for the Care and Use of Laboratory Animals published by the National Institutes of Health.

### Generation of tubule-specific Erbin knockout mouse

Homozygous Erbin floxed mice (C57BL/6J background, Strain NO. T008906), the transgenic mice expressing Cre recombinase under the control of tubular epithelial-specific Cdh16 (Ksp) promoter (C57BL/6 J background, Strain NO.T004820), and the mG/mT reporter mice (B6/JGpt-H11^em1Cin(CAG-LoxP-ZsGreen-Stop-LoxP-tdTomato)^/Gpt, Strain NO. T006163) were purchased from GemPharmatech Co, Ltd (Nanjing, China). To generate TEC-specific Erbin null mice, we crossed the Erbin^flox/flox^ mice with Ksp-Cre transgenic mice. A routine PCR protocol was used for genotyping of tail DNA samples with the following primer pairs: Cre transgene, 5’-GGGCAGTCTGGTACTTCCAAGCT-3’ and 5’-ACTGAGTGCCTACTAACCAGCACC-3’, which generated a 353 bp fragment; and Erbin genotyping, 5’-CAAGTTAGACTTTGGCATGGTGG-3’ and 5’-TCCTACACACTGCACATTTACACG-3’, which yielded a 385 bp band for the floxed alleles, and a 280 bp band for wild-type alleles. To assess the specificity of Cre-recombinase expression, Ksp-Cre mice were crossed with mG/mT reporter mice that express a cell membrane-targeted, two-color fluorescent Cre-reporter allele [B6/JGpt-H11^em1Cin(CAG-LoxP-ZsGreen-Stop-LoxP-tdTomato)^/Gpt]. The reporter mice express membrane-localized EGFP (mG) fluorescence widespread in cells/tissues prior to Cre recombination. After crossing with the Cre transgenic mice, the membrane-localized tdTomato (mT) fluorescence replaces green fluorescence in the cells where Cre-mediated recombination occurs. All animals were born normally at the expected Mendelian frequency in normal size, and did not display any gross physical or behavioral abnormalities.

### Cell culture and treatment

Rat renal tubular epithelial cell NRK-52E was obtained from the American Type Culture Collection (Manassas, VA). Human embryonic kidney 293T cell was purchased from the Cell Bank of the Chinese Academy of Sciences (Shanghai, China). All cells were maintained in DMEM medium (Invitrogen, Camarillo, CA) containing 10% fetal calf serum and incubated at 37 °C in a humidified atmosphere of 5% CO_2._ For hypoxia treatment, confluent NRK-52E cells were incubated with four volumes of mineral oil on top of one volume of low-serum medium (DMEM with 1% serum) for 1 h at 37 °C, followed by extensive washing and reperfusion in full medium for the indicated times (Kormann et al, 2020). To induce oxidative stress, NRK-52E cells were incubated with H_2_O_2_ at a final concentration of 250 μM in full medium. For cisplatin treatment, NRK-52E cells were incubated with cisplatin at a final concentration of 20 μM for 6 h, changed with the medium without cisplatin, and then continued to culture the cells.

### Generation of Erbin knockout NRK-52E cells

To knock out Erbin in NRK-52E cells, three different single-guide RNA (sgRNA) constructs, which target exons 1, 5, and 6, respectively, were designed using the online CRISPR design tool (http://crispr.mit.edu/). The sgRNA expression constructs were cloned into the LentiGuide-Puro plasmid, respectively. To obtain lentivirus, 293T cells were transfected with LentiGuide-Puro, the packaging plasmid psPAX2, and the VSV-G envelope plasmid pMD2.G using the Lipofectamine 2000 reagent. After infection with the lentivirus capable of expressing Cas9, the NRK-52E cell lines overexpressing Cas9 were selected using 15 μg/ml of blasticidin. The Erbin knockout cell lines were then obtained after infecting with lentivirus expressing sgRNA and selecting with 15 μg/ml of blasticidin and 2 μg /ml of puromycin. The clones were screened at the mRNA and protein levels. To ensure the successful editing of Erbin in the genome, we performed T7 Endonuclease I (T7E1) assay according to the previously described method (Zhao et al, 2024). Briefly, the genomic DNA was extracted from NRK-52E, NRK-52E-NC, and NRK-52E-KO cells. The genomic regions covering the editing sites were amplified with specific primers, respectively. The PCR products were purified, denatured by heating, and annealed with the denatured amplicon from NRK-52E cells to form heteroduplex DNA. The samples were then incubated with T7E1 (NEB, Ipswich, MA, USA) for 30 min at 37 °C and analyzed by agarose gel electrophoresis.

### Isolation of primary tubular epithelial cells

Tubular epithelial cells were isolated from mice according to the previously described method with modification (Kormann et al, 2020; Luo et al, 2018). Briefly, the kidneys were harvested, decapsulated, and dissected visually. The renal cortical tissues were minced thoroughly into pieces of 1 mm^3^ followed by dissociation in DMEM/F-12 medium. The minced fragments were collected by centrifuge, transferred to 0.2% (wt/vol) collagenase II solution (Sigma-Aldrich), and incubated for 30 min at 37 °C. Afterward, the digested tissue was washed with DMEM/F-12 medium three times, and sieved successively through 70-μm and 40-μm nylon mesh to isolate the tubular fragments. The primary tubular cells were then collected by centrifugation for 5 min at 500 rpm, plated in 10 cm dishes, and cultured in DMEM-F12 medium supplemented with 10% FBS with both streptomycin and penicillin. Fresh media was supplemented every alternate day until they reached 60% to 80% confluency.

### Cell cycle analysis by flow cytometry

Cell cycle assay was performed according to the previously described method. Briefly, cells were trypsinized, centrifuged, and fixed with 70% ice-cold ethanol. The fixed cells were incubated with RNase A and propidium iodide (PI) staining solution in the dark at room temperature for 60 min. Cell cycle phases were analyzed and quantified using a BD FACScalibur flow cytometer (BD Biosciences) and Modifit software.

Cell cycle analysis of primary tubular cells was performed according to the previously described method (Kormann et al, 2020). Briefly, renal cortical tissues were manually minced, and dissociated in PBS, 0.5% BSA, and 2 mM EDTA in a GentleMACS Dissociator. The homogenate was filtered through a 30-μm filter and collected by centrifugation. The cells were then incubated with a CDH16 antibody conjugated with Alexa Fluor^®^ 647 (Novus Biologicals, Centennial, CO), followed by nuclear DNA staining with DAPI. An IgG-647 isotype was used as a control. The CDH16^+^ tubular cell population was selected, and the cell cycle phases were analyzed and quantified using an Attune NxT flow cytometer (Thermo Fisher) and FlowJo v10.0 software (BD Biosciences).

### Cell proliferation assay

NRK-52E cells were seeded on a 96-well plate in triplicate at an initial number of 5000 cells per well. Cell proliferation was determined at the indicated times using the Cell Counting kit-8 (Byeotime, Nantong, China) according to the manufacturer’s protocol. The OD450 absorbance values were determined on a double-channel Microplate Reader (Tecan Group Ltd., Männedorf, Switzerland). Cell proliferation was calculated on the basis of absorbency.

### EdU assay

Cells were cultured with the medium containing 50 μM EdU for 2 h at 37 °C. Afterward, cells were washed with PBS, fixed with 4% paraformaldehyde, and stained according to the manufacturer’s instructions. The nucleus was counterstained by mounting medium containing 4’, 6-diamidino-2-phenylindole (DAPI). Images were captured by a ZEISS LSM980 laser scanning confocal microscope (ZEISS, Oberkochen, Germany). The percentage of EdU-positive cells was quantified using Image-Pro Plus 6.0 (Media Cybernetics, Bethesda, MD, https://mediacy.com/products/image-pro-plus/).

### Plasmid

The murine Erbin cDNA was amplified by RT-PCR with primers as follows: Forward: 5’- ATGACTACAAAACGAAGTTTGTTTGTG-3’ and Reverse: 5’- TTATGAAGAAACTTCTCGTACAATGATG-3’. The PCR fragment was inserted into the pMD18-T vector, sequenced, and then cloned into the mammalian expression plasmid pCI to obtain pCI-Erbin. The expression plasmid for Myc-tagged Erbin was generated by inserting Erbin cDNA into pCMV-Myc. The cDNA encoding murine YB-1 was amplified by RT-PCR with primers as follows: Forward: 5’-ATGAGCAGCGAGGCCGAG-3’ and Reverse: 5’- TTACTCAGCCCCGCCCTG-3’. The LMO7 cDNA was amplified by RT-PCR with primers as follows: Forward: 5’- ATGGAAGGGATGGAGGACG-3’ and Reverse: 5’- TCACATGGCGGTTGGCCG-3’. The validated YB-1 and LMO7 cDNA were then cloned into the pCI plasmid to yield pCI-YB-1 and pCI-LMO7, respectively.

### Oligonucleotides and transfection

A set of YB-1-specific siRNA duplexes was designed and synthesized by GenePharma (Shanghai, China). siRNAs were introduced into NRK-52E cells using Lipofectamine 2000 (Invitrogen). Unless otherwise indicated, the transfection mix contained 100 nM siRNA in this study. The siRNA sequences are listed in Table EV1.

### ABT-263 senolytic treatment

ABT-263 (GLPbio, Montclair, CA) was prepared in 10% DMSO, 40% polyethylene glycol 400, and 5% Tween 80 in saline. The vehicle was the above mixture without the drug. For senolytic treatment, mice were administered with ABT-263 by gavage at a dose of 50 mg/kg daily for two cycles from 1 day after I/R. Each cycle contained 5 consecutive daily injections and a subsequent 2-day interval. The sham operation group of mice were treated with vehicle. Kidneys were harvested 14 days after I/R.

### In situ administration of the virus

The adeno-associated virus serotype 9 (AAV9) carrying the full length of murine YB-1 and the adenovirus carrying the full length of murine Erbin were packaged, purified, and titrated by OBiO Biotechnology (Shanghai, China). The in situ injection of the virus in the cortex of the mouse kidney was carried out using the previously described method. Briefly, after exposing the left kidney of mice under anesthesia, the renal vein was clamped with a microaneurysm clamp. The recombinant adenovirus (1 × 10^10^ particles) carrying Erbin or control sequence, and recombinant AAV9 (1 × 10^11^ particles) carrying YB-1 or control sequence, were diluted in 100 μl of saline, and injected into the left renal vein using a 31-gauge needle. The clamp was removed 15 min after injection, followed by suturing of the incision.

### Ultrasound-mediated gene transfer of plasmids into the kidney

Ultrasound-mediated transfer of plasmid into the kidneys was performed according to the previously published method. Briefly, the mixture of 200 μl plasmid/Sonouge (Bracco, Milan, Italy) at a 1:1 ratio (volume:volume) was injected into the tail vein. After injection, the ultrasound probe was immediately placed on the skin opposite to the kidney for 4 min of ultrasound treatment (2 W/cm^2^).

### RNA isolation and quantitative real-time PCR

Total RNA was prepared from the cells or kidney tissues by homogenization and purification using TRIzol Reagent (Invitrogen, Carlsbad, CA, USA). For mRNA detection, 500 ng of total RNA were used for complementary DNA synthesis and real-time PCR was performed in triplicate using the SYBR PrimeScript RT-PCR Kit (Takara) in StepOne™ Real-Time PCR System (Thermo Fisher) according to the manufacturer’s instructions. Individual gene expression was quantified using a standard curve and normalized to β-actin mRNA expression. The PCR conditions were as follows: 95 °C for 30 s, and then 40 cycles of amplification for 5 s at 95 °C and 30 s at 60 °C. Primers are listed in Table EV1.

### Transcriptome sequencing and bioinformatic analysis

Total RNA was extracted from Erbin knockout NRK-52E and the control cells. The cDNA library construction, high-throughput sequencing, and bioinformatic analysis were performed at Novogene Co., Ltd (Beijing, China). Differentially expressed genes (DEGs) were identified on the basis of multiple-test corrected *P* value and fold change by the following criteria: *P* < 0.05 and change (FC) ≥1.5 or ≤−1.5.

### Immunoprecipitation and mass-spectrometry assay

The NRK-52E cells were lysed in lysis buffer (50 mM Tris·HCl, pH 7.4, 150 mM NaCl, 1% Triton X-100, 1 mM PMSF, 1 mM EDTA, 2 mM Na_3_VO_4_, and protease inhibitor mixtures). The lysates were centrifuged and pre-absorbed with protein A/G magnetic beads (Invitrogen) for 2 h at 4 °C with rotation. The precleared supernatant was incubated with 1 μg indicated antibodies at 4 °C overnight with rotation. The protein A/G beads were then added and further incubated for 4 h at 4 °C. Beads were washed four times with lysis buffer. The immunoprecipitates were resolved in SDS-PAGE gels. To identify the proteins that interact with the Myc-Erbin fusion protein, the band specific to Myc antibody, as well as the corresponding gel in the control lane, was excised, respectively, and followed by mass-spectrometry (MS) identification.

### Western blot

Cell pellets or kidney tissues were lysed in lysis buffer (20 mM Tris-HCl, pH 7.5, 150 mM NaCl, 1% Triton X-100, sodium pyrophosphate, β-glycerophosphate, EDTA, Na_3_VO_4_ and leupeptin) containing 1 mM PMSF. After mixing with 2×Laemmli buffer and heat denaturation at 100 °C for 5 min, equal amounts of lysate (20 μl) were separated by SDS-PAGE and then transferred onto PVDF membranes. The membranes were probed by antibodies at appropriate dilutions for 1 h. After washing with PBST, the membrane was then probed with horseradish peroxidase-conjugated anti-mouse, anti-rabbit, or anti-goat IgG for 1 h at room temperature. The protein bands were visualized by fluorography using an enhanced chemiluminescence system (Cell Signaling Technology, Beverly, MA).

### Histological examination and evaluation

For histological examination and immunostaining, the excised kidney tissues were fixed in 4% paraformaldehyde and embedded in paraffin. After deparaffinization and rehydration, the sections were stained with hematoxylin and eosin (HE), periodic acid Schiff (PAS), and Masson’s trichrome, respectively, according to the manufacturer’s instructions. The images were captured using a forward microscope (Nikon DS-Ri2, Tokyo, Japan). Tubular injury was evaluated as simultaneous quantification of tubular dilation, tubular necrosis, and cast formation, and was scored semi-quantitatively on PAS staining, according to the following scale: 0, no tubular damage; 1, damage in 1–25% of the tubules analyzed; 2, damage in 26–50% of the tubules analyzed; 3, damage in 51–75% of the tubules analyzed; 4, damage in >76% of the tubules analyzed. The mean value was calculated for each mouse. Semiquantitative analysis of interstitial fibrosis was determined on Masson’s trichrome staining using Image-Pro Plus 6.0 in a blind manner. The percentage of blue collagen staining, avoiding large blood vessels, was calculated. Quantifications were performed by examining at least 10 microphotographs of random, non-overlapping fields at 200× magnification per mouse. All samples were evaluated in a blind manner.

### SA-β-gal staining

SA-β-gal staining of NRK-52E cells was performed using the Senescence β-Galactosidase Staining Kit (Beyotime Technology, Shanghai, China). Briefly, the cells were fixed, and then incubated with fresh β-galactosidase staining solution (pH 6.0) at 37 °C for 12 h. SA-β-gal-positive cells were counted under a microscope at 100× magnification and expressed as a percentage of total cells. For the kidney, SA-β-gal activity was assayed using frozen sections (3 μm) according to the manufacturer’s instructions. The SA-β-gal staining was quantified as a percentage of the blue-stained area using ImageJ software (NIH, https://imagej.nih.gov/ij/).

### Immunohistochemistry and immunofluorescence

Immunohistochemistry and immunofluorescence staining were carried out using the protocols previously established in our laboratory. For immunostaining of cells seeded on coverslips, the cells were washed with cold PBS, fixed in 4% formaldehyde, permeabilized with 0.5% Triton X-100, and incubated with the specific primary antibody overnight at 4 °C. Afterward, cells were incubated with Alexa 488 or 594 conjugated secondary antibodies, followed by DAPI counterstain. For p-H3 staining of NRK-52E cells, actin filaments were labeled with DyLight 594 Phalloidin (Cell Signalling Technology, #12877). For kidney tissue stain, we performed the single or dual immunostaining in 4-μm formalin-fixed, paraffin-embedded kidney sections using a microwave-based antigen retrieval technique. Briefly, the sections were rehydrated and subjected to antigen retrieval by microwaving in 0.01 M sodium citrate (pH 6.0) for 10 min. The sections were then blocked in TBS-T containing 10% FBS and 0.1% Triton X-100, and incubated with the primary antibodies against the indicated protein overnight. The signal was detected with secondary antibodies conjugated with Alexa Fluor or horseradish peroxidase. The nuclei were counterstained with DAPI. Images were captured using a ZEISS LSM980 laser scanning confocal microscope. The whole kidneys were longitudinally cut into 3-μm sections and scanned at ×20 magnification under a Leica TCS SP8-MP confocal microscope (Leica, Wetzlar, Germany). The images were then stitched to create a large-scan image using Leica LAS X software (https://www.leica-microsystems.com/products/microscope-software/details/product/leica-las-x-ls/).

For immunostaining quantifications, 10 random (x200) or 20 random (x400) visual fields were analyzed per animal (sample). The quantification of collagen I-, KIM-1- and α-SMA- positive area were expressed as a percentage of total tissue area. The nuclear stains of γH2AX, p-H3, p21, and Ki67 were identified by positive nuclear staining for fluorescence. YB-1^+^ and α-SMA ^+^ cells were defined as the cells, where 75% of the cell area, immediately surrounding the DAPI-stained nucleus, was positive for the fluorescence. Their results were expressed as the percentage of total cells. The quantification of p-H3^+^ Ki67^+^ positive tubular cells were expressed as the percentage of Ki67^+^ p-H3^+^ cells relative to total Ki67^+^ tubular cells.

Erbin immunohistochemistry staining score was quantified to evaluate the expression level according to previously described methods with some modifications. Briefly, staining intensity and the extent of staining were set as two criteria. The staining intensity of tubular cells was scored as 0 (no staining), 1 (weak staining), 2 (intermediate staining), and 3 (strong staining). The extent of staining was scored semi-quantitatively according to the following scale: 0, 1–4% positively stained cells; 1, 5–25% positively stained cells; 2, 26–50% positively stained cells; 3, 51–75% positively stained cells; and 4, ≥75% positively stained cells. For each sample, ten photographs of non-contiguous sections at 200× magnification were quantified in a blind manner. The multiple of the average intensity and extent score was used as the final score (0–12).

### Measurement of SCR, BUN, urinary albumin, and urinary creatinine levels

Serum creatinine, BUN, and urinary creatinine levels were determined using commercial kits according to the manufacturer’s instructions (Nanjing Jiancheng Bioengineering Institute, Nanjing, China). Urinary albumin concentration was measured using a mouse Albumin ELISA Quantitation kit according to the manufacturer’s protocol (Elabscience, Wuhan, China). Urinary albumin was standardized to urine creatinine, and was expressed as μg per mg urinary creatinine.

### Human kidney tissue samples

Kidney biopsy samples from patients and normal controls were obtained from the Center of Kidney Disease, 2nd Affiliated Hospital, Nanjing Medical University, Nanjing, China. We used the healthy portions of nephrectomy specimens from patients who underwent surgery due to malignancies as the normal control. Human renal biopsy samples were from CKD patients who were confirmed with DN, IgAN, or ATN. The samples were fixed in 10% (v/v) formalin and embedded in paraffin blocks. The study involving human samples was approved by the Science and Technology Ethics Committee of Nanjing University (No. AP20230309002), and performed in accordance with the Helsinki Declaration of 1975, as revised in 1983.

### Statistical analysis

The data were analyzed for normal distribution using GraphPad Prism 8 (GraphPad Software). Two-tailed unpaired Student’s *t*-test and two-tailed unpaired Student’s *t*-test with Welch’s correction were conducted for the comparison of two groups. One-way ANOVA followed by Dunnett’s or Tukey’s multiple comparison test, and Brown–Forsythe and Welch ANOVA with Tamhane’s multiple comparisons test were used for the comparison of multiple groups. Survival rates were analyzed using a survival curve with the log-rank test. Spearman’s correlation analysis was performed to determine the correlation between two variables. No animals or data points were excluded from the analysis for any particular reason. Data were expressed as the means ± s.e.m. The details of the statistical analysis were indicated in the individual figure legends. A value of *P* < 0.05 was considered statistically significant.

### Graphics

Figure 8C and the synopsis graphics were created with BioRender.com.

## Supplementary information

Appendix

Table EV1

Peer Review File

Dataset EV1

Dataset EV2

Source data Fig. 1

Source data Fig. 2

Source data Fig. 3

Source data Fig. 4

Source data Fig. 5

Source data Fig. 6

Source data Fig. 7

Source data Fig. 8

Expanded View Figures

## Data Availability

The RNA-seq data have been deposited in the Gene Expression Omnibus (GEO) under the accession code GSE274147 (https://www.ncbi.nlm.nih.gov/geo/query/acc.cgi?acc=GSE274147). The data that support the findings of this study are available from the corresponding author upon reasonable request. The source data of this paper are collected in the following database record: biostudies:S-SCDT-10_1038-S44318-026-00885-y.
