# Supplementary material for: Loss of Erbin in tubular epithelial cells sensitizes mice to acute kidney injury and aggravates renal fibrosis
Source: EMBO J. 2026 Aug 24;45(19):6782–812. doi: 10.1038/s44318-026-00885-y (PMC13631325; doi:10.1038/s44318-026-00885-y)
Supplement: Supplementary file 1 [file 44318_2026_885_MOESM14_ESM.pdf]

Expanded View Figures

Figure EV1. Erbin is implicated in tubular cell senescence in the IRI kidney.

(**A**) Representative images of SA-β-gal, p21, γH2AX, and LMNB1 staining from kidneys of WT and Erbin knockout mice on day 14 after I/R. LMNB1 was co-stained with LTL and PNA, respectively. Nuclei were visualized using DAPI staining. Scale bar for SA-β-gal staining, 100 μm; Scale bar for p21 and γH2AX staining, 50 μm; Scale bar for LMNB1 staining, 20 μm. $n = 5$ per group. (**B**) The quantification of SA-β-gal, p21, γH2AX, and LMNB1 staining for (a). (**C**) Kidney p16 and Klotho expression were examined by Western blot. (**D**) Kidney Klotho and PAI-1 expression were examined by qRT-PCR. The results are shown as fold change compared with WT mice without IRI. (**E**) Representative images of SA-β-gal, p21, γH2AX and LMNB1 staining from Adv-Erbin-injected kidneys on day 14 after I/R. LMNB1 was co-stained with LTL and PNA, respectively. Nuclei were visualized using DAPI staining. Scale bar for SA-β-gal staining, 100 μm; Scale bar for p21 and γH2AX staining, 50 μm; Scale bar for LMNB1 staining, 20 μm. $n = 5$ per group. (**F**) The quantification of SA-β-gal, p21, γH2AX, and LMNB1 staining for (**E**). (**G**) Kidney p16 and Klotho expression were examined by Western blot. (**H**) Kidney Klotho and PAI-1 expression were examined by qRT-PCR. Results are presented as the mean ± s.e.m. *P* values were determined using one-way ANOVA with Tukey's multiple comparison test in (**B–D**), and one-way ANOVA with Dunnett's multiple comparisons test in (**F–H**).

© The Author(s)

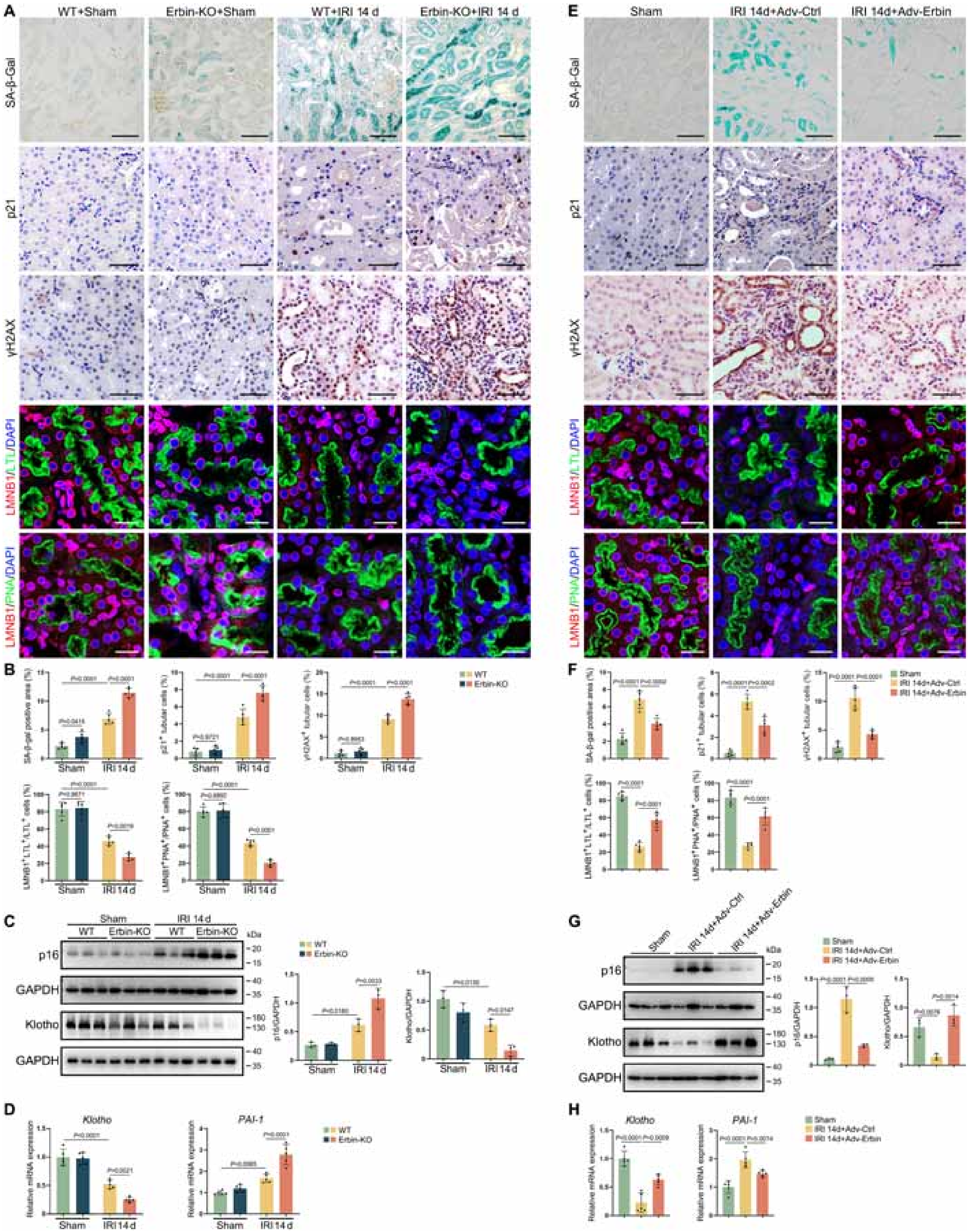
A
WT+Sham
Erbin-KO+Sham
WT+IRI 14 d
Erbin-KO+IRI 14 d
SA-β-Gal
p21
γH2AX
LMNB1/LTL/DAPI
LMNB1/PNA/DAPI
E
Sham
IRI 14d+Adv-Ctrl
IRI 14d+Adv-Erbin
B
WT
Erbin-KO
Sham
IRI 14 d
F
C
p16
GAPDH
Klotho
kDa
D
Klotho
PAI-1
Relative mRNA expression
G
H

 © The Author(s)

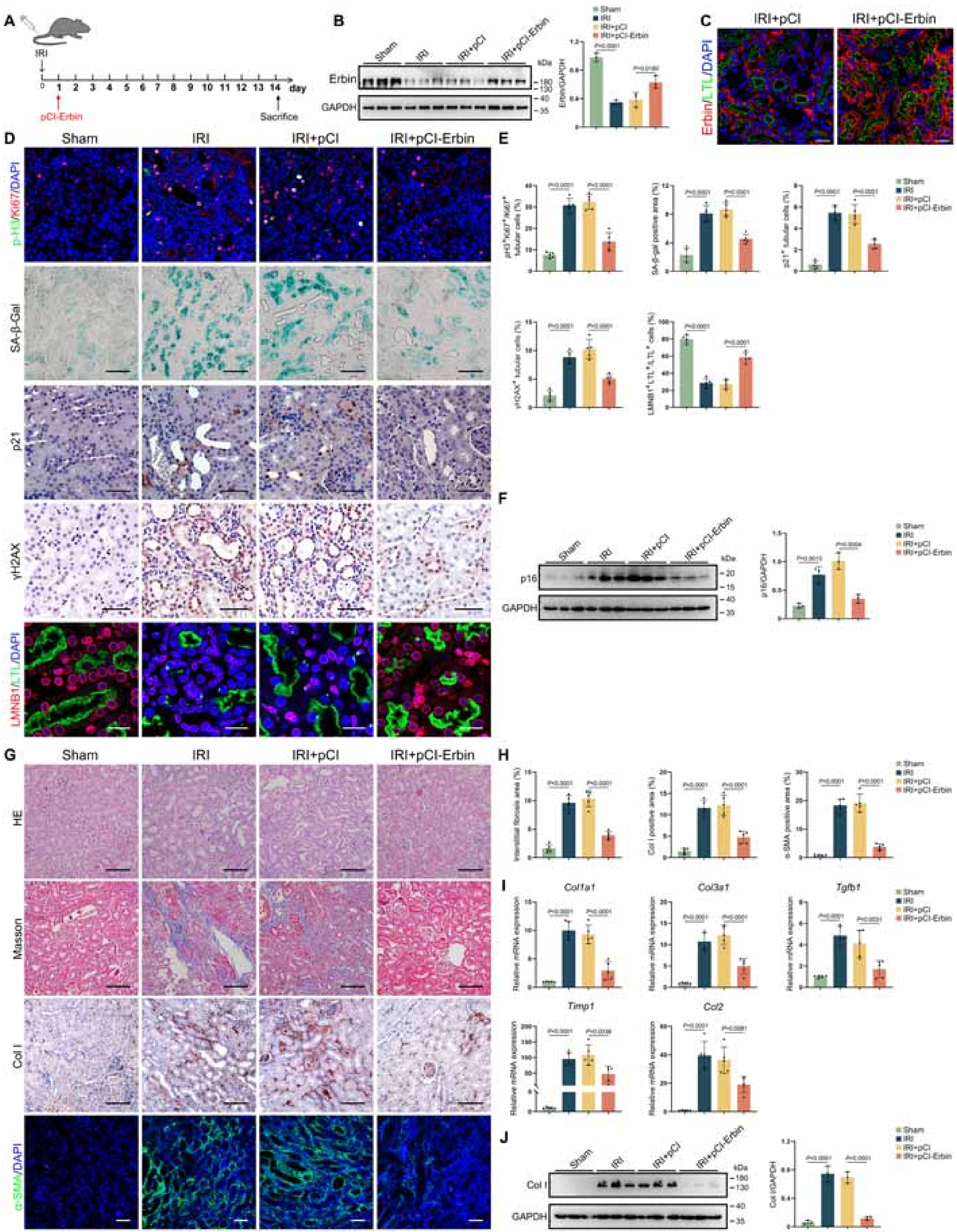
A
IRI
0 1 2 3 4 5 6 7 8 9 10 11 12 13 14 day
pCI-Erbin
Sacrifice
B
Sham IRI IRI+pCI IRI+pCI-Erbin
Erbin
GAPDH
kDa
C
IRI+pCI IRI+pCI-Erbin
Erbin/LTL/DAPI
D
Sham IRI IRI+pCI IRI+pCI-Erbin
p-H3/Ki67/DAPI
SA-β-Gal
p21
γH2AX
LMNB1/LTL/DAPI
E
F
p16
GAPDH
G
Sham IRI IRI+pCI IRI+pCI-Erbin
HE
Masson
Col I
α-SMA/DAPI
H
I
Col1a1
Col3a1
Tgfb1
Timp1
Ccl2
J
Col I
GAPDH

Figure EV2. Overexpression of Erbin in the established IRI kidney mitigates tubular senescence and prevents renal fibrosis.

(**A**) Schematic diagram of Erbin overexpression after IRI surgery. The pCI-Erbin plasmid was delivered into the kidney 1 day after IRI surgery using the ultrasound microbubble-mediated gene transfer technique. Mice were divided into four groups: Sham, IRI, IRI with delivery of control plasmid (IRI + pCI), and IRI with delivery of pCI-Erbin. Kidneys were harvested at 14 days after I/R ($n = 5$ per group). (**B**) Ebin was examined by Western blot. (**C**) Representative images of cortical kidney sections stained for Erbin and LTL. Nuclei were visualized using DAPI staining. Scale bar, 50 μm. (**D**) Representative images of pH3/Ki67, SA-β-gal, p21, γH2AX, and LMNB1 staining. LMNB1 was co-stained with LTL. Scale bar for SA-β-gal staining, 100 μm; Scale bar for pH3/Ki67, p21, and γH2AX staining, 50 μm; Scale bar for LMNB1 staining, 20 μm. (**E**) Dot plots showing the quantification of pH3/Ki67, SA-β-gal, p21, γH2AX, and LMNB1 staining. (**F**) p16 was examined by Western blot. (**G**) Representative images of HE and Masson staining, and immunohistochemistry for collagen I and immunostaining for α-SMA in mouse kidneys. Scale bars for HE, Masson and Col I IHC, 100 μm; Scale bars for α-SMA immunostaining, 50 μm. (**H**) Quantitative analysis of tubular interstitial fibrosis from Masson staining, and quantification of Col I and α-SMA staining area. (**I**) The mRNA expression of *Col1a1, Col3a1, Tgfb1, Ccl2*, and *Timp 1* were examined by qRT-PCR. The results are shown as fold change compared to WT mice subjected to IRI. (**J**) Kidney collagen I was detected by Western blot. Results are presented as the mean ± s.e.m. *P* values were determined using one-way ANOVA with Tukey's multiple comparison test.

 © The Author(s)

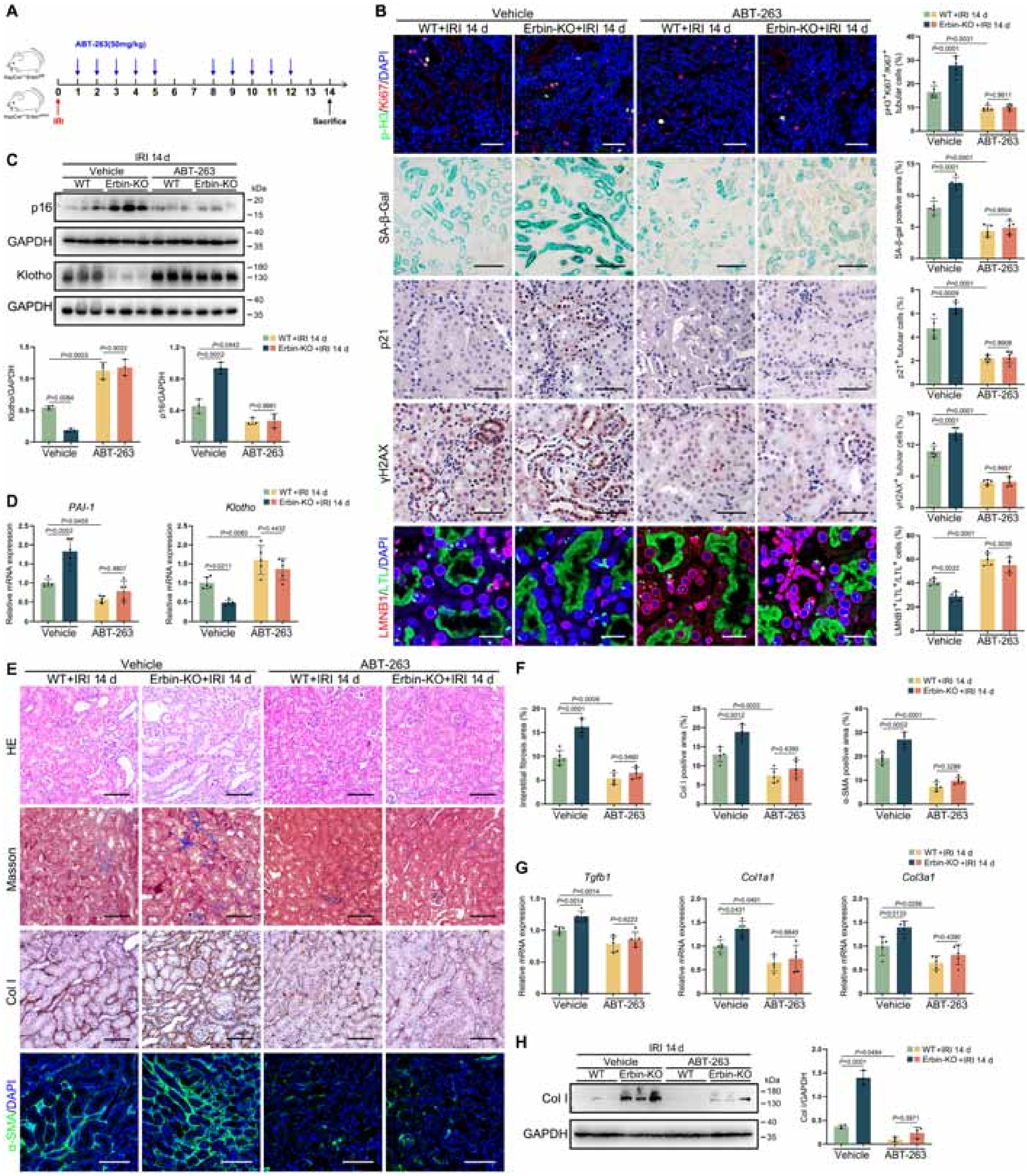
A
ABT-263(50mg/kg)
0 1 2 3 4 5 6 7 8 9 10 11 12 13 14
IRI
Sacrifice
B
Vehicle
ABT-263
WT+IRI 14 d
Erbin-KO+IRI 14 d
p-H3/Ki67/DAPI
SA-β-Gal
p21
γH2AX
LMNB1/LTL/DAPI
WT+IRI 14 d
Erbin-KO+IRI 14 d
Vehicle
ABT-263
C
IRI 14 d
Vehicle
ABT-263
WT
Erbin-KO
p16
GAPDH
Klotho
kDa
Klotho/GAPDH
p16/GAPDH
D
PAI-1
Klotho
Relative mRNA expression
E
HE
Masson
Col I
α-SMA/DAPI
F
Interstitial fibrosis area (%)
Col I positive area (%)
α-SMA positive area (%)
G
Tgfb1
Col1a1
Col3a1
H
Col I
Col I/GAPDH

Figure EV3. The detrimental effect of Erbin loss on renal fibrosis is associated with tubular senescence.

(**A**) Schematic diagram of the IRI model with ABT-263 treatment. ABT-263 was administered by gavage daily for two cycles, each of 4 days, from 1 day after I/R. Kidneys were harvested at 14 days after I/R ($n = 5$ per group). (**B**) Representative images of pH3/Ki67, SA-β-gal, p21, γH2AX, and LMNB1 staining. LMNB1 was co-stained with LTL. Nuclei were visualized using DAPI staining. Scale bar for SA-β-gal staining, 100 μm; Scale bar for pH3/Ki67, p21, and γH2AX staining, 50 μm; Scale bar for LMNB1 staining, 20 μm. $n = 5$ per group. Dot plots showing the quantification of pH3/Ki67, SA-β-gal, p21 and γH2AX staining. (**C**) p16 and Klotho were examined by Western blot. (**D**) PAI-1 and Klotho expression were examined by qRT-PCR. (**E**) Representative images of HE, Masson staining, immunohistochemistry for collagen I, and immunostaining for α-SMA in kidneys. Scale bars, 100 μm. (**F**) Quantitative analysis of tubular interstitial fibrosis from Masson staining, and quantification of Col I and α-SMA staining area. (**G**) The mRNA expression of kidney *Tgfb1*, *Col1a1*, and *Col3a1* were examined by qRT-PCR. The results are shown as fold change compared to the vehicle-treated WT mice. (**H**) Kidney collagen I was detected by Western blot. Results are presented as the mean ± s.e.m. *P* values were determined using one-way ANOVA with Tukey's multiple comparison test.

 © The Author(s)

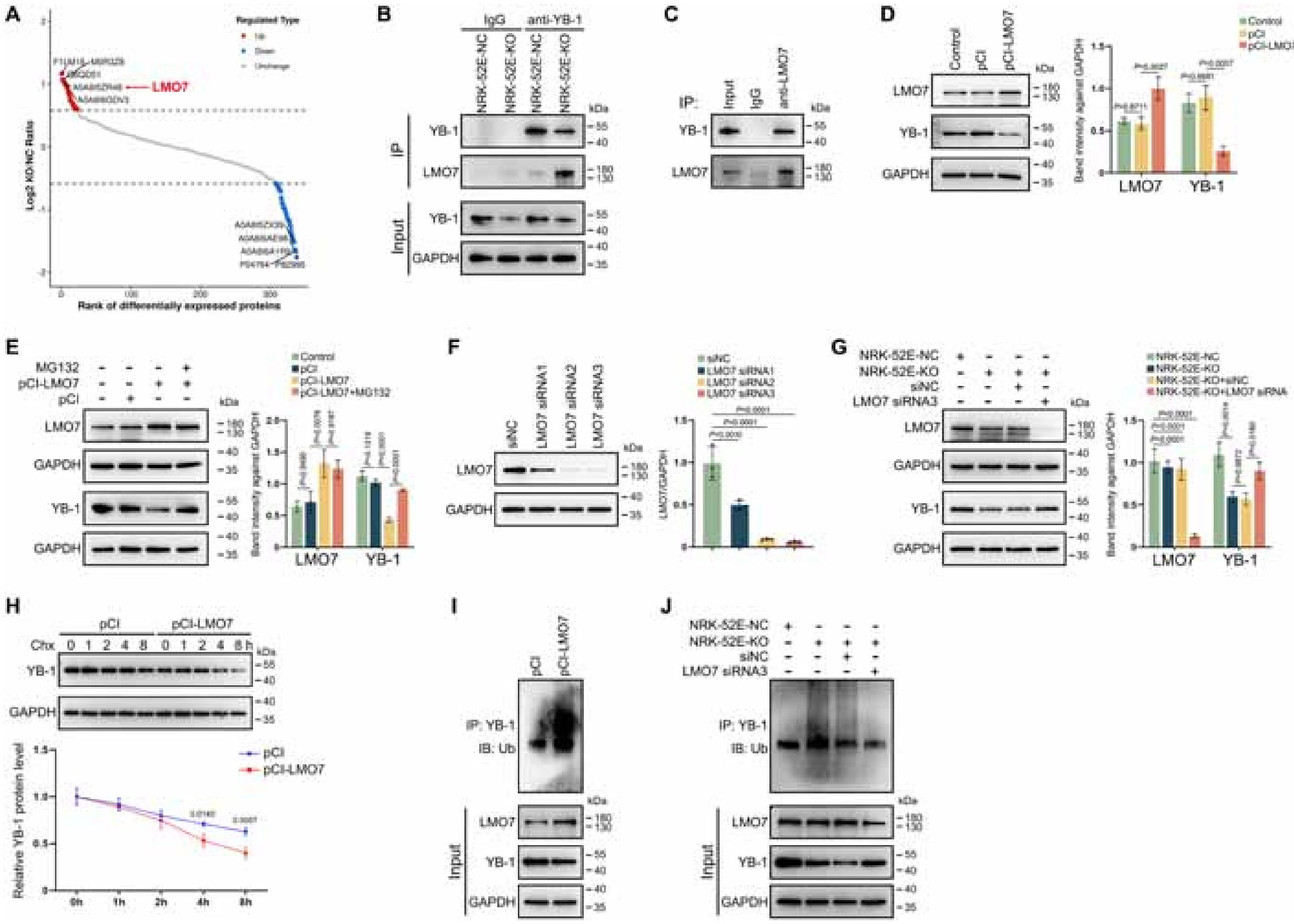

Figure EV4. Erbin protects YB-1 from degradation through interfering the LMO7-mediated ubiquitination.

(**A**) The change of the YB-1-interacting proteins by Erbin deletion. NRK-52E-KO and the control cells were subjected to IP using an anti-YB-1 antibody, and an MS assay of the precipitates. Red, the significantly enriched YB-1-interacting proteins by Erbin deletion; Blue, significantly decreased proteins by Erbin deletion. The arrow points to LMO7. (**B**) The interaction between YB-1 and LMO7 was tested by IP assay of NRK-52E-KO cells using an anti-YB-1 antibody. The retrieved protein was detected by Western blot. (**C**) NRK-52E cells were subjected to an IP assay using the LMO7 antibody. The retrieved protein was detected by Western blot. (**D**) LMO7 overexpression reduced YB-1 production in NRK-52E cells. NRK-52E cells were transfected with the pCI-LMO7 plasmid for 48 h. (**E**) MG132 abolished the inhibitory effect of LMO7 overexpression on YB-1 in NRK-52E cells. (**F**, **G**) Silencing LMO7 abrogated the inhibitory effect of Erbin deficiency on YB-1 production in NRK-52E cells. (**H**) LMO7 overexpression reduced the half-time of the YB-1 protein. NRK-52E cells were transfected with the pCI-LMO7 plasmid for 24 h, and then treated with 100 µg/mL cycloheximide and harvested at various time points as indicated for western blot. The remaining YB-1 protein was expressed as a percentage relative to that at the beginning time. (**I**) LMO7 overexpression promoted the ubiquitination of YB-1 in NRK-52E cells. (**J**) Silencing LMO7 abrogated the increase of YB-1 ubiquitination in the Erbin-deficient NRK-52E cells. The above experiments were repeated three times. Results are presented as the mean ± s.e.m. *P* values were determined using one-way ANOVA with Dunnett's multiple comparison test in (**D**, **F**), one-way ANOVA with Tukey's multiple comparison test in (**E**, **G**), and two-tailed unpaired Student's *t*-test in (**H**).

© The Author(s)

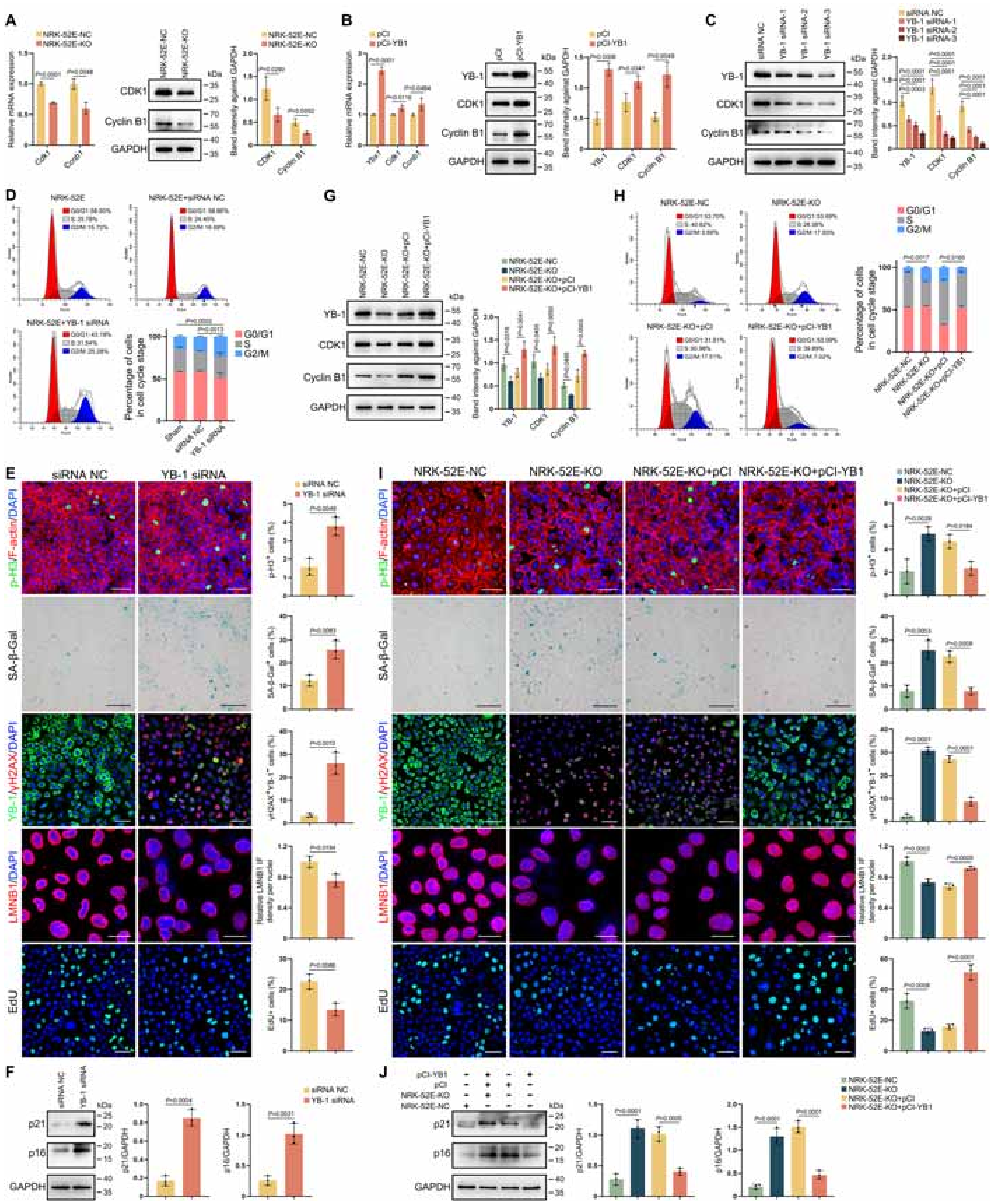
A
NRK-52E-NC
NRK-52E-KO
CDK1
Cyclin B1
GAPDH
B
pCI
pCI-YB1
YB-1
CDK1
Cyclin B1
GAPDH
C
siRNA NC
YB-1 siRNA-1
YB-1 siRNA-2
YB-1 siRNA-3
YB-1
CDK1
Cyclin B1
GAPDH
D
NRK-52E
NRK-52E+siRNA NC
NRK-52E+YB-1 siRNA
G0/G1
S
G2/M
Percentage of cells in cell cycle stage
G
NRK-52E-NC
NRK-52E-KO
NRK-52E-KO+pCI
NRK-52E-KO+pCI-YB1
YB-1
CDK1
Cyclin B1
GAPDH
H
NRK-52E-NC
NRK-52E-KO
NRK-52E-KO+pCI
NRK-52E-KO+pCI-YB1
G0/G1
S
G2/M
Percentage of cells in cell cycle stage
E
siRNA NC
YB-1 siRNA
p-H3/F-actin/DAPI
SA-β-Gal
YB-1/γH2AX/DAPI
LMNB1/DAPI
EdU
I
NRK-52E-NC
NRK-52E-KO
NRK-52E-KO+pCI
NRK-52E-KO+pCI-YB1
p-H3/F-actin/DAPI
SA-β-Gal
YB-1/γH2AX/DAPI
LMNB1/DAPI
EdU
F
p21
p16
GAPDH
siRNA NC
YB-1 siRNA
J
pCI-YB1
pCI
NRK-52E-KO
NRK-52E-NC
p21
p16
GAPDH
NRK-52E-NC
NRK-52E-KO
NRK-52E-KO+pCI
NRK-52E-KO+pCI-YB1

Figure EV5. Erbin protects the tubular cells from senescence by interacting with YB-1.

(**A**) Deletion of Erbin significantly reduces the expression of CDK1 and Cyclin B1 in NRK-52E cells, as indicated by qRT-PCR (left) and western blot (right). (**B**) Overexpression of YB-1 leads to a significant increase in CDK1 and Cyclin B1 in NRK-52E cells. NRK-52E cells were transfected with the pCI-YB-1 plasmid for 48 h. (**C**) Knockdown of YB-1 greatly reduces CDK1 and Cyclin B1 in NRK-52E cells. NRK-52E cells were transfected with a set of YB-1 siRNA or the control for 48 h. (**D**) Cell cycle analysis by flow cytometry shows that knocking down YB-1 increased the proportion of cells in G2/M phase. (**E**) Representative images of p-H3 immunostaining, SA-β-gal staining, γH2AX /YB-1 dual immunostaining, LMNB1 immunostaining, and EdU staining after knocking down YB-1 in NRK-52E cells. Scale bar for LMNB1 immunostaining, 20 μm. Scale bar for others, 50 μm. The dot plot shows the corresponding quantification of the images, respectively. (**F**) Knockdown of YB-1 increases p21 and p16 in NRK-52E cells. (**G**) Ectopic expression of YB-1 restores CDK1 and Cyclin B1 in Erbin knockout NRK-52E cells. (**H**) Flow cytometry showing that restoring YB-1 reduced the proportion of G2/M phase in Erbin knockout NRK-52E cells. (**I**) Representative images of p-H3 immunostaining, SA-β-gal staining, γH2AX/YB-1 dual immunostaining, LMNB1 immunostaining, and EdU staining for Erbin knockout NRK-52E cells overexpressing YB-1. Scale bar for LMNB1 immunostaining, 20 μm. Scale bar for others, 50 μm. The dot plot shows the corresponding quantification of the images, respectively. The above experiments were repeated three times. (**J**) Ectopic expression of YB-1 inhibits p21 and p16 in Erbin knockout NRK-52E cells. Results are presented as the mean ± s.e.m. *P* values were determined using two-tailed unpaired Student's *t*-test in (**A**, **B**, **E**, **F**), one-way ANOVA with Dunnett's multiple comparison test in (**C**), and one-way ANOVA with Tukey's multiple comparison test in (**D**, **G**, **H**, **I**, **J**).

© The Author(s)
