# Supplementary material for: Loss of Erbin in tubular epithelial cells sensitizes mice to acute kidney injury and aggravates renal fibrosis
Source: EMBO J. 2026 Aug 24;45(19):6782–812. doi: 10.1038/s44318-026-00885-y (PMC13631325; doi:10.1038/s44318-026-00885-y)
Supplement: Supplementary file 2 [file 44318_2026_885_MOESM1_ESM.pdf]

Loss of Erbin in tubular epithelial cells sensitizes mice to acute kidney injury and aggravates renal fibrosis

Fang Shao[1], Zhengrong Ren[1], Lei Shi[1], Yanyan Sun[1], Wen Sun[1], Jing Tang[1], Yingsailamu[1], Zhi Ding[1], Junfeng Zhang[1,]*, Yuhui Zang[1,]*

[1]*State Key Laboratory of Pharmaceutical Biotechnology, School of Life Sciences, Nanjing University, Nanjing 210093, PR China*

Table of contents

Appendix Table S1 3

Appendix Figure S1 4

Appendix Figure S2 5

Appendix Figure S3 6

Appendix Figure S4 7

Appendix Figure S5 8

Appendix Figure S6 9

Appendix Figure S7 10

Appendix Figure S8 11

Appendix Figure S9 12

Appendix Figure S10...13

Appendix Figure S11...15

Appendix Figure S12...16

Appendix Figure S13...17

Appendix Figure S14...18

Appendix Figure S15...19

Appendix Figure S16……………………………………………………………………………20

Appendix Figure S17……………………………………………………………………………21

Appendix Figure S18……………………………………………………………………………22

Appendix Figure S19……………………………………………………………………………23

Appendix Figure S20……………………………………………………………………………24

Appendix Figure S21……………………………………………………………………………25

Appendix Table S1 Characteristics of the CKD patients

	All	Control	DN	ATN	IgAN
N (%)	40	12 (30.00%)	9 (20.45%)	10 (25.00%)	9 (20.45%)
Age (years)	43.93±9.26	40.00±4.84	51.67±8.52	45.20±14.20	40.00±8.00
Gender (male, n, %)	22 (55.00%)	6 (50.00%)	5 (55.56%)	7 (70.00%)	4 (44.44%)
SCR (μmol/l)	180.09±115.83	69.88±14.73	165.93±55.56	391.92±116.46	15.82±30.65
BUN (mmol/l)	9.68±5.49	4.35±0.82	9.32±3.82	18.63±7.63	7.18±1.53
eGFR (ml/min/1.73 m^2)	62.78±34.92	106.7±13.04	44.78±18.09	17.70±8.61	73.15±23.12

Appendix Figure S1

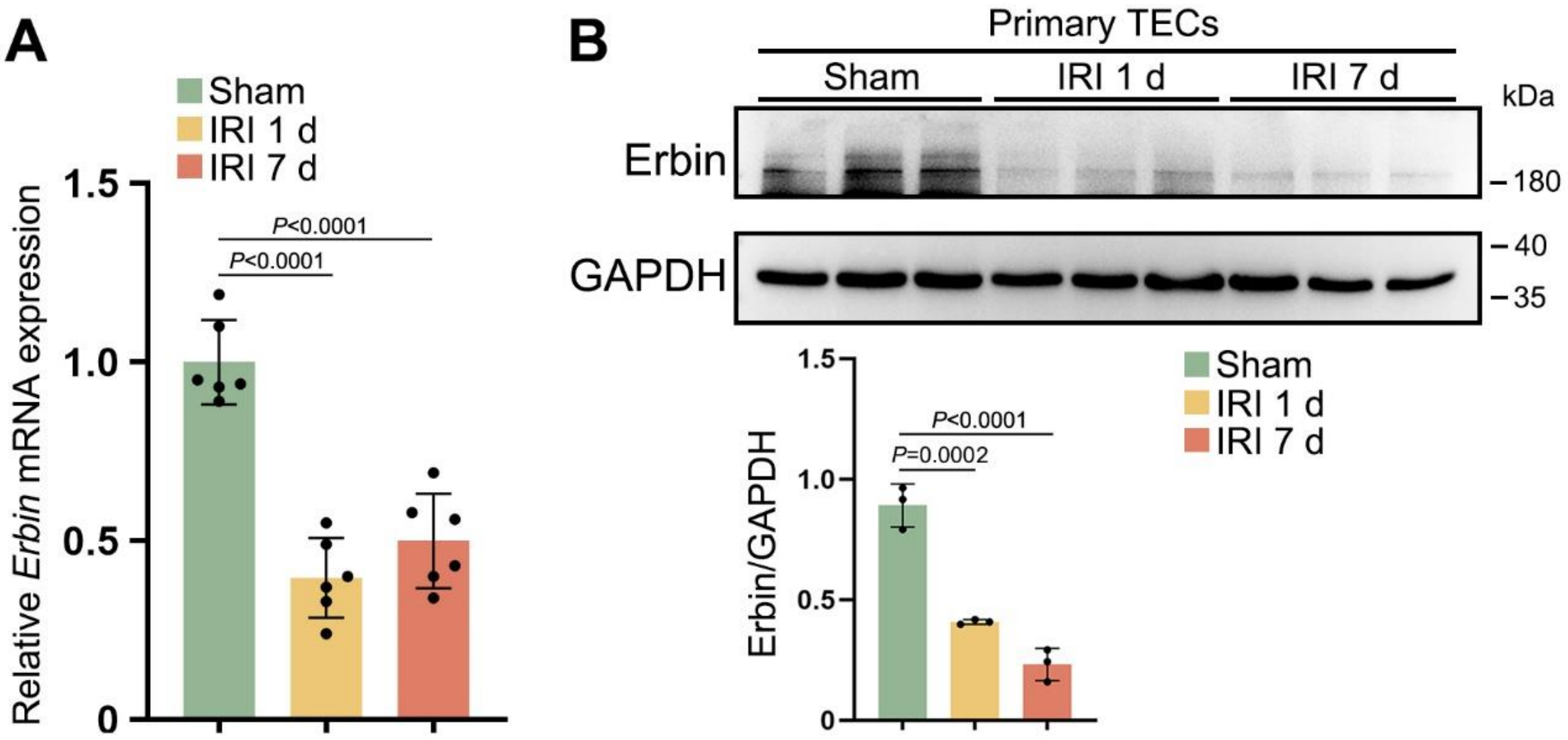

Appendix Figure S1. Erbin is decreased in the tubular cells after IRI. Primary tubular cells were isolated from mice at indicated times after IRI operation (n=6 for each group). Erbin expression in the isolated tubular cells was determined by qRT-PCR (A) and western blot (B). Data are presented as the mean ± s.e.m. Statistical analyses were conducted using one-way ANOVA with Dunnett's multiple comparison test.

Appendix Figure S2

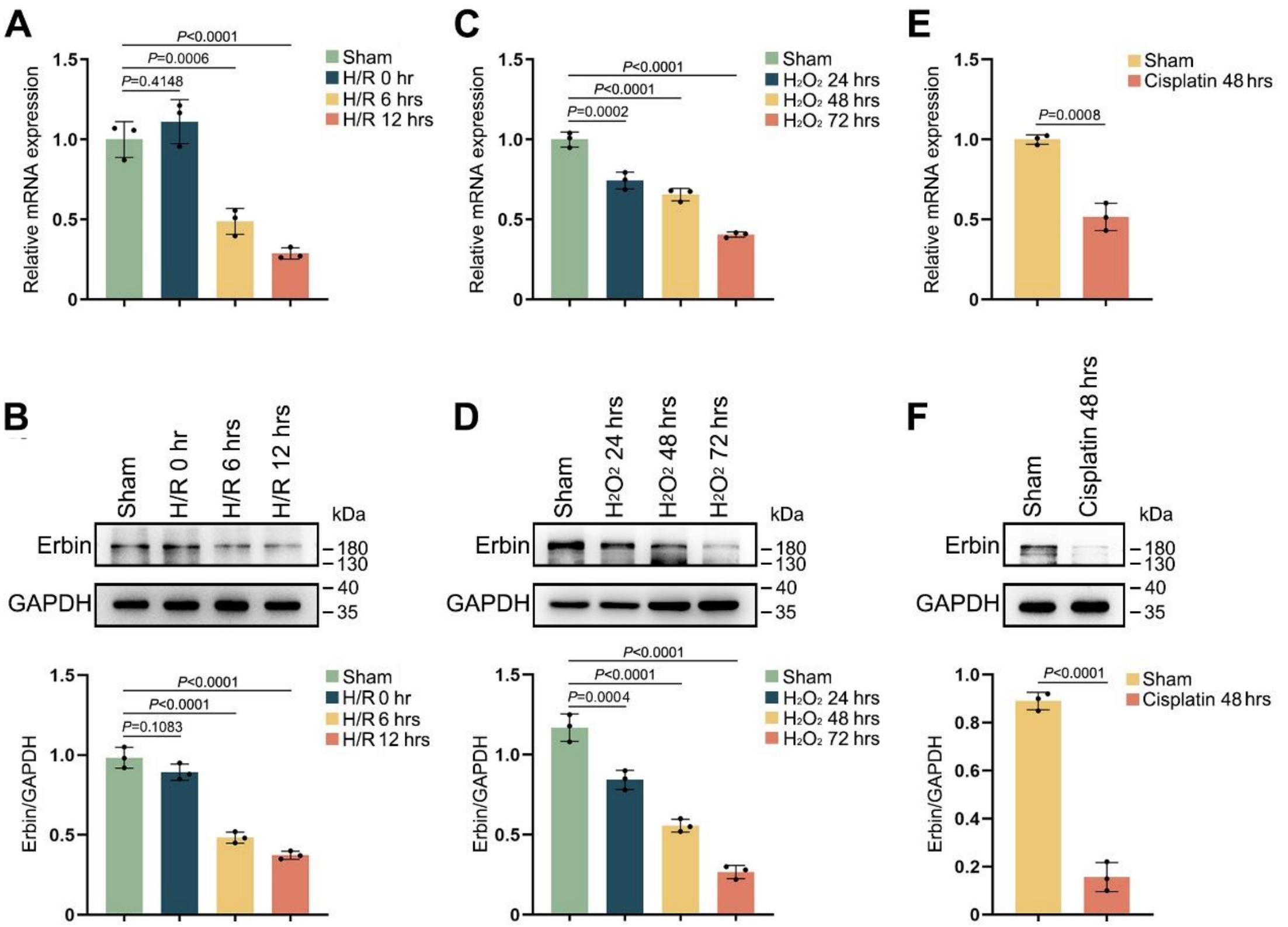

Appendix Figure S2. Erbin is decreased in the injured NRK-52E cells. Erbin expression was determined by qPCR and western blot. (A and B) The confluent NRK-52E cells were incubated with four volumes of mineral oil on the top of low-serum medium (1% serum) for one hour at 37°C, followed by extensive washing and reperfusion in full medium for indicated times. qRT-PCR results are shown as fold change compared with cells without hypoxia/re-oxygenation (H/R) treatment. (C and D) NRK-52E cells were cultured in the presence of 250 mM H_2O_2 for indicated times. qRT-PCR results are shown as fold change compared with cells without H_2O_2 treatment. (E and F) NRK-52E cells were incubated with cisplatin at a final concentration of 20 μM for 6 hours, changed with the medium without cisplatin, and then continued to culture the cells for 48 hours. qRT-PCR results are shown as fold change compared with cells without cisplatin treatment. The above experiments were repeated three times. Data are presented as the mean ± s.e.m. Statistical analyses were

conducted using one-way ANOVA with Dunnett's multiple comparison test in A-D, and two-tailed unpaired Student's t-test in E and F.

Appendix Figure S3

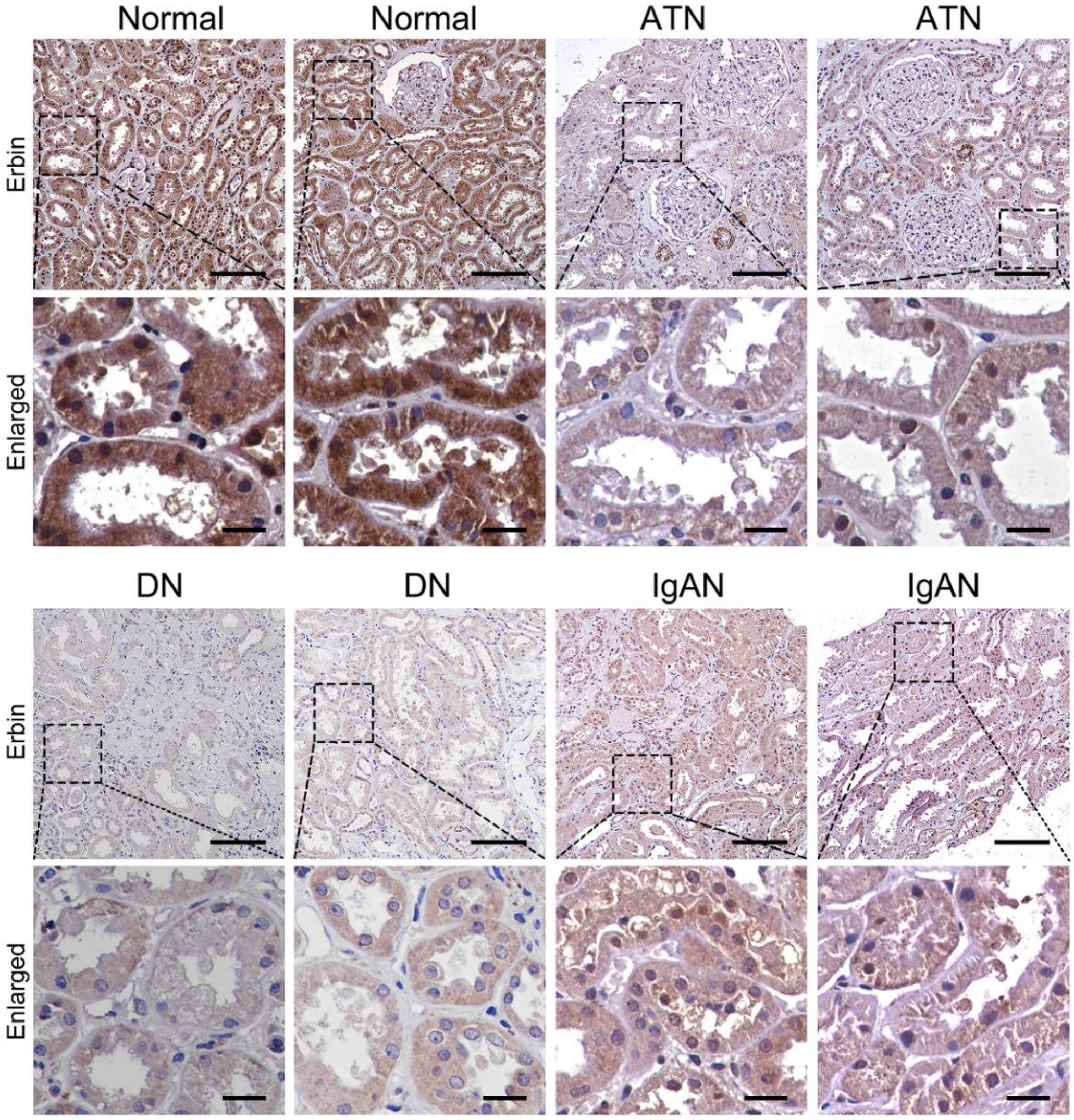

Appendix Figure S3. Erbin is decreased in the tubular cells of CKD patients. Representative images of Erbin immunostaining of kidney biopsies from ATN (n=10), DN (n=9), and IgAN (n=9) patients (scale bar, 100 μm). Nontumor kidney tissue from the patients who had renal cell carcinoma and underwent nephrectomy was used as normal controls (n=18). The bottom images (scale bar, 25 μm) are enlarged from the boxed areas in the middle images.

Appendix Figure S4

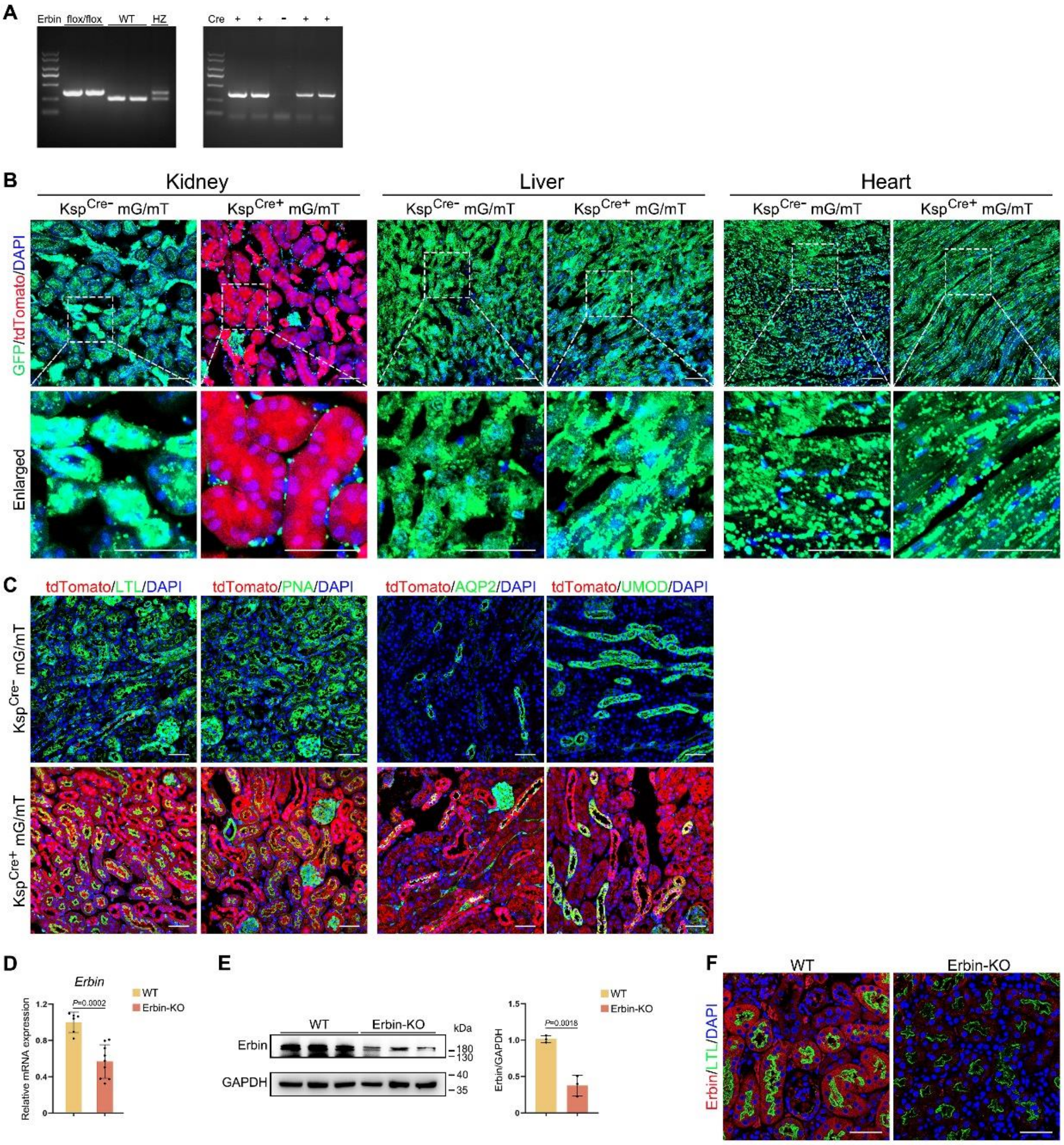

Appendix Figure S4. Generation and confirmation of tubule-specific Erbin knockout mice. (A) The genotype of tubule-specific deletion of Erbin was confirmed by PCR analysis of tail DNA. Bands of WT or floxed Erbin alleles were observed at ~280 bp and 350 bp, respectively. The expression of Cre transgene was visualized by a 360 bp band. (B) Tubular tdTomato reporter (red fluorescence) characteristic of Cre recombinase activity in mG/mT (B6/JGpt-H11$^{em1Cin(CAG-LoxP-ZsGreen-Stop-LoxP-tdTomato)}$/Gpt) mice (green fluorescence) crossed with Ksp-Cre mice. The boxed areas are enlarged. Scale bar, 50 μm. (C) Representative images of kidney sections stained for tdTomato with tubular

specific markers (LTL, PNA, AQP2, and UMOD), respectively. Nuclei were visualized using DAPI staining. Scale bar, 50 μm. (D and E) Erbin expression in the cortex of kidney was examined by qPCR and western blot. (F) Representative images of kidney sections stained for Erbin (red) and LTL (green). Scale bar, 50 μm. Data are presented as the mean ± s.e.m. Statistical analyses were conducted using two-tailed unpaired Student's t-test.

Appendix Figure S5

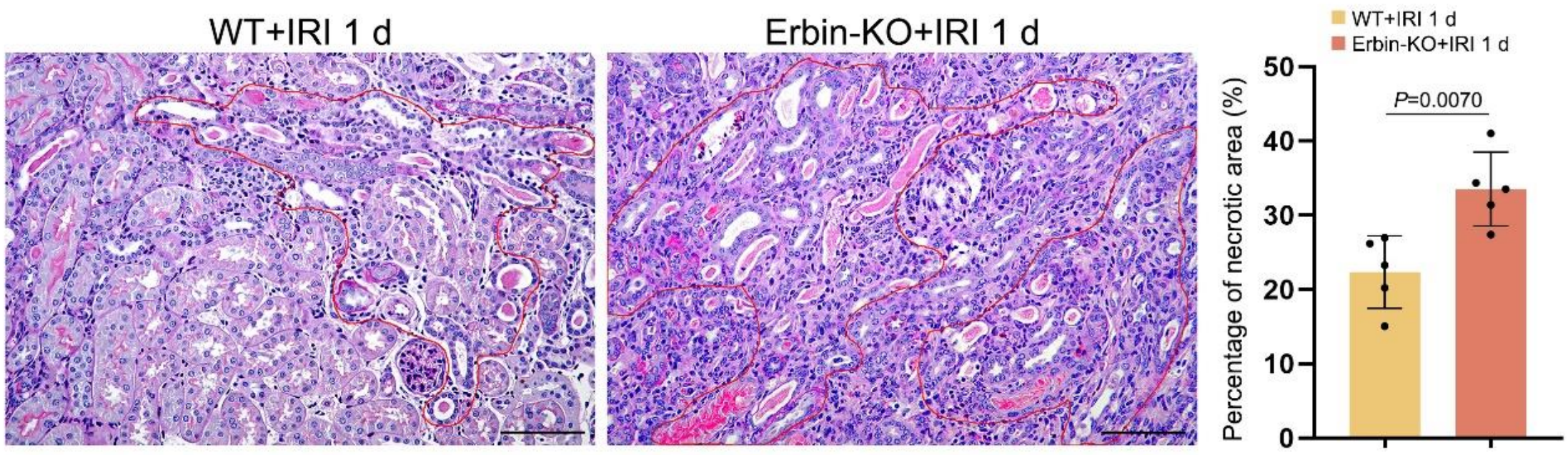

Appendix Figure S5. Tubule-specific deletion of Erbin exacerbates necrotic cell death at 1 day after IRI. The necrotic area, defined by a red line, was determined by PAS staining (*n* = 5 for each group). Scale bar, 100 μm. Dot plot shows the percentage of necrotic area. Statistical analysis was conducted using two-tailed unpaired Student's t-test.

Appendix Figure S6

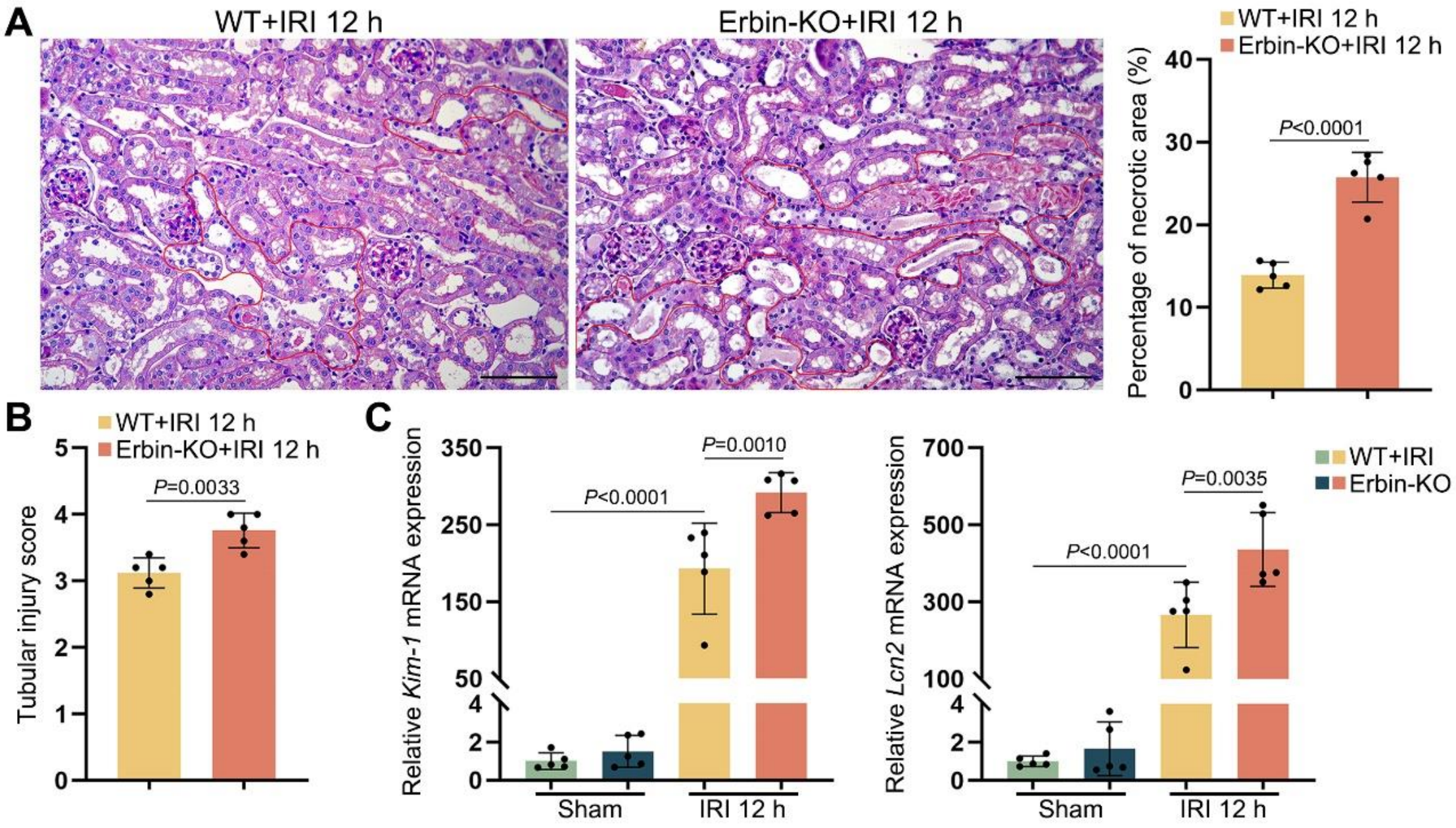

Appendix Figure S6. Tubule-specific deletion of Erbin aggravates tubular damage and the extent of necrotic cell death at 12 hours after IRI. Erbin-KO and WT mice were sacrificed at 12 hours after IRI (n=5 per group). (A) The necrotic area, defined by a red line, was determined by PAS staining. Scale bar, 100 μm. Dot plot shows the percentage of necrotic area. (B) Tubular injury score. (C) KIM-1 and NGAL expression were significantly augmented in Erbin deficient mice at 12 hours after IRI as indicated by qRT-PCR. Statistical analysis was conducted using two-tailed unpaired Student's t-test in A and B, and one-way ANOVA with Tukey's multiple comparison test in C.

Appendix Figure S7

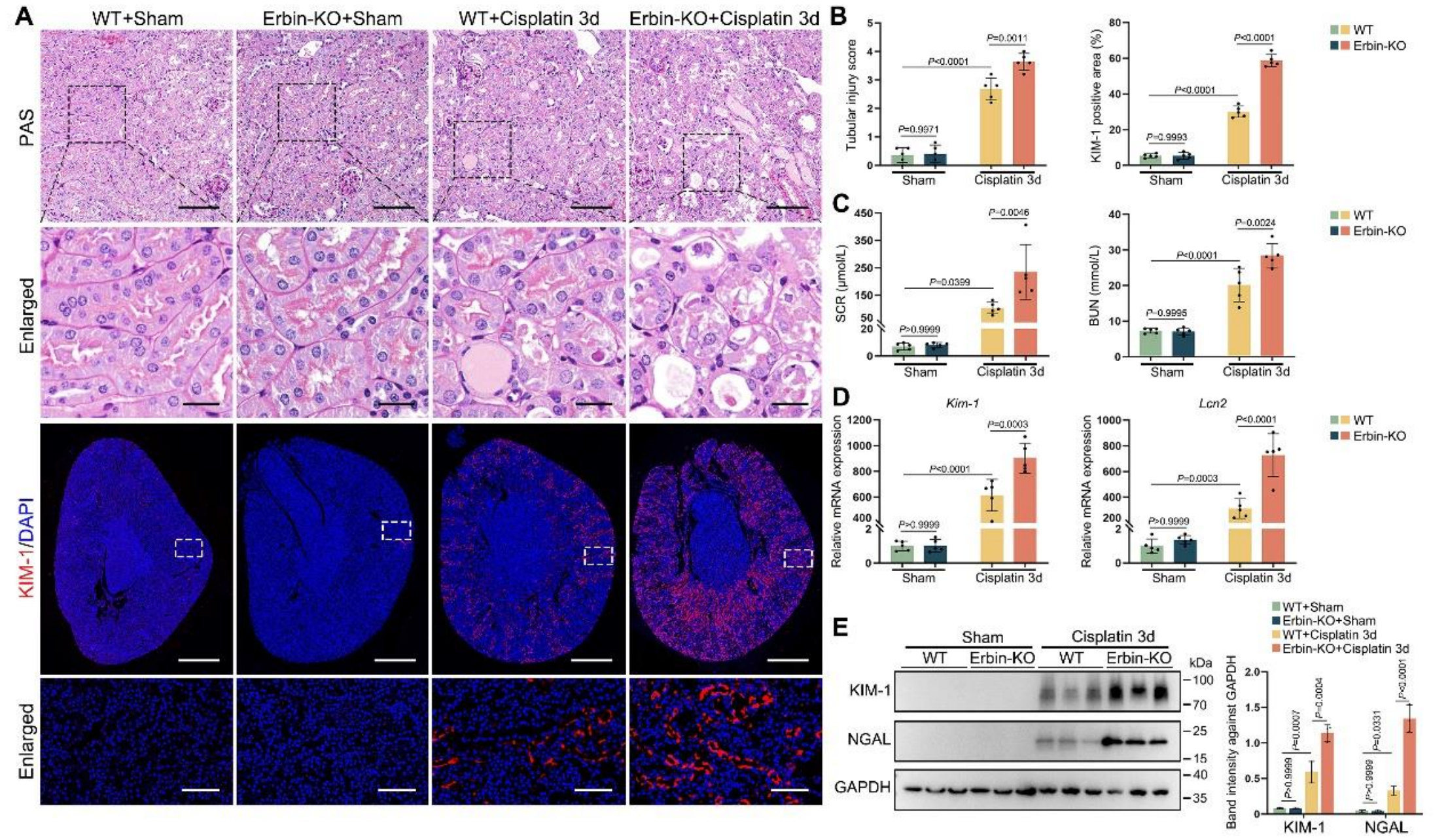

Appendix Figure S7. Tubule-specific Erbin deficiency exacerbates cisplatin-induced AKI in mice. The Erbin-KO and WT mice were sacrificed at 3 days after a single injection of cisplatin. (n=5 per group). (A) PAS staining and immunostaining for KIM-1 showing exacerbated tubular damage in Erbin-KO mice. Scale bar for PAS staining, 100 μm, and the boxed areas are enlarged (Scale bar, 25 μm). KIM-1 immunostaining images of whole kidney cross-sectional area are stitched at ×200 magnification (Scale bar, 1000 μm), and the bottom panels are enlarged from the boxed areas (scale bar, 100 μm). (B) Tubular injury score and quantification of KIM-1 staining in cortex and outer medulla from whole kidney section images. (C) SCR and BUN levels. (D and E) KIM-1 and NGAL expression were examined by qRT-PCR and western blot. Results are presented as the mean ± s.e.m. Statistical analyses were conducted using one-way ANOVA with Tukey's multiple comparison test.

Appendix Figure S8

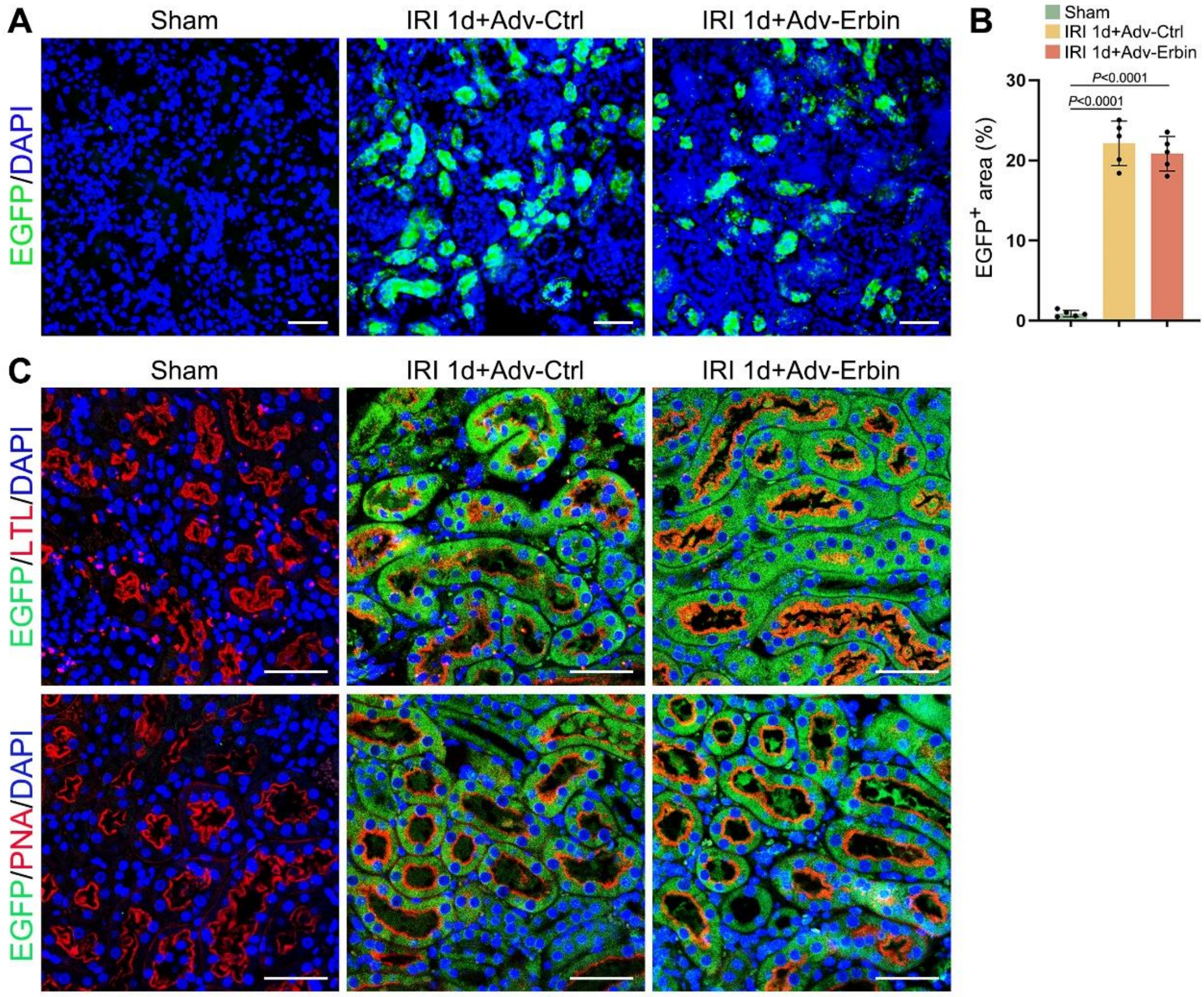

Appendix Figure S8. Immunofluorescence demonstrates the infection efficacy and distribution of adenovirus in the kidney. (A) Kidney sections were examined for EGFP expression by immunofluorescence at 7 days after the *in situ* injection (n=5 per group). Scale bar, 50 μm. (B) EGFP expression was quantitatively assessed by measuring the immunofluorescence area (right). (C) Validation of the segment-specific distribution of adenovirus by co-staining EGFP with proximal tubular marker LTL, and distal tubular marker PNA respectively. Scale bar, 50 μm. Results are presented as the mean ± s.e.m. Statistical analyses were conducted using one-way ANOVA with Dunnett’s multiple comparison test.

Appendix Figure S9

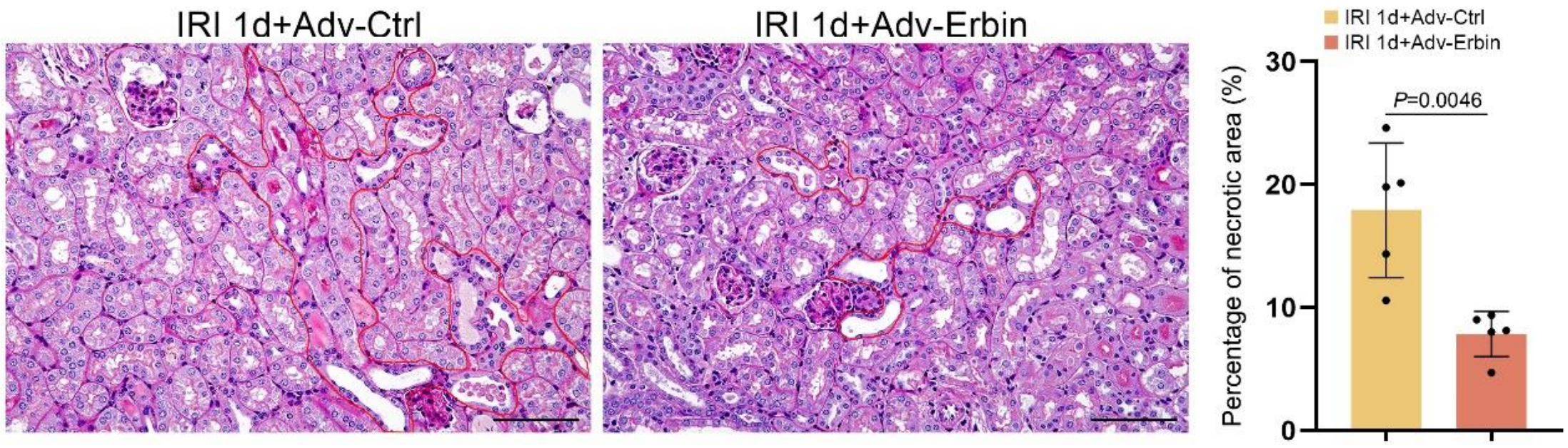

Appendix Figure S9. Ectopic expression of Erbin alleviates the IRI-induced necrotic cell death in mice. The necrotic area, defined by a red line, was determined by PAS staining at one day after IRI, *n* = 5 for each group. Scale bar, 100 μm. Dot plot shows the percentage of necrotic area. Statistical analyses were conducted using two-tailed unpaired Student's t-test.

Appendix Figure S10

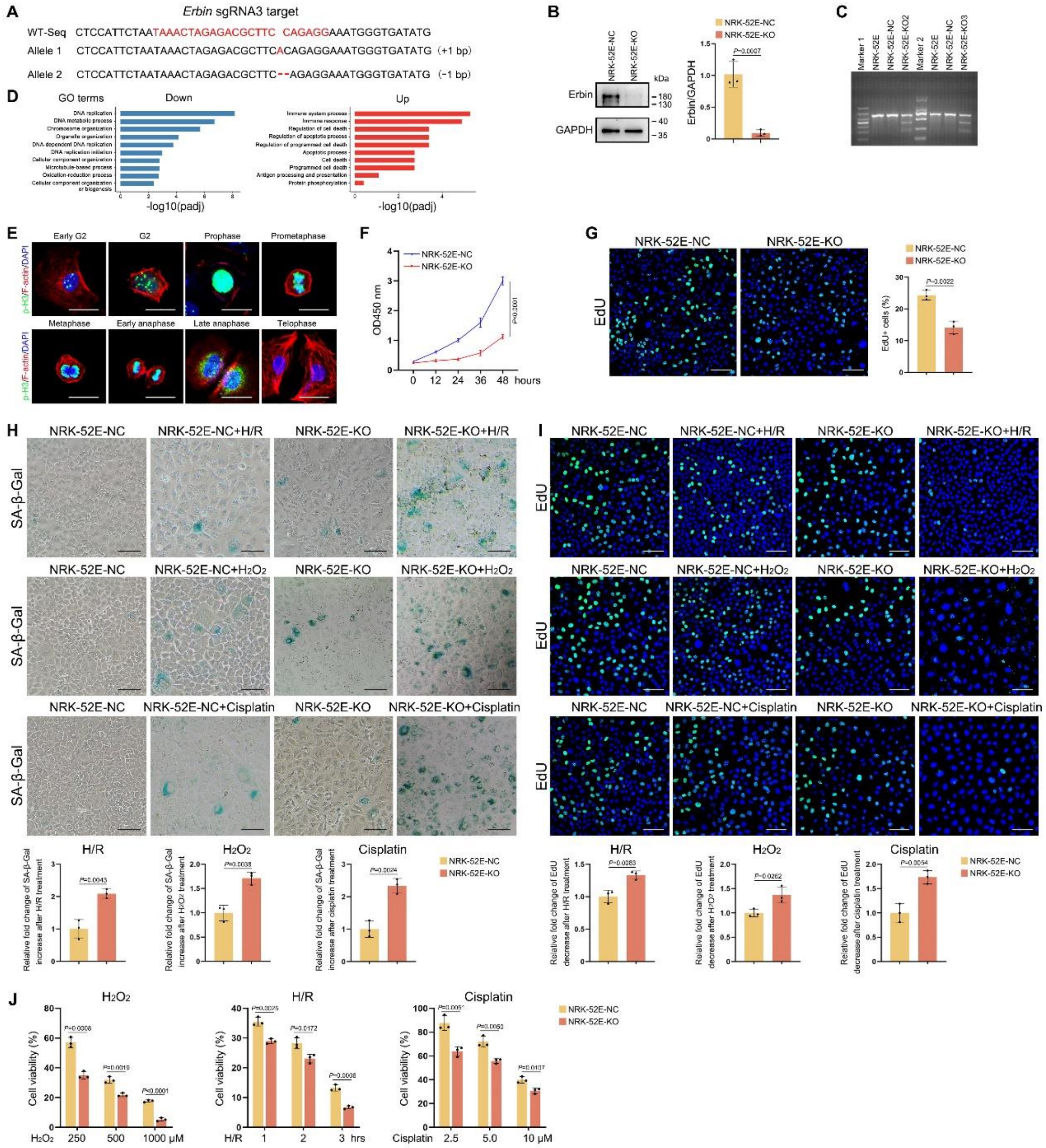

Appendix Figure S10. Identification of the Erbin knockout NRK-52E cells. Three sgRNA constructs targeting the Erbin exon 1, 5, and 6 were designed to generate Erbin knockout cell lines. The subline NRK-52E-KO2, referred to as NRK-52E-KO, was used for further investigation in this study. (A) DNA sequencing validated the indels genotype of two *Erbin* alleles in NRK-52E-KO. (B) Deletion of Erbin was confirmed by western blot. (C) CRISPR-Cas9 genome editing of Erbin was confirmed by T7 Endonuclease I (T7E1) assay. (D) Function enrichment analysis of differentially regulated

genes (DEGs) by Gene Ontology (GO) on biological processes between NRK-52E-KO and NRK-52E-NC cells. Bars represent the top terms in the classification of downregulated DEGs (blue), or upregulated DEGs (red). (E) Validation of p-H3 as G2/M phase marker. Chromatin patterns of NRK-52E cells were shown with immunostaining of antibody against p-H3. (F) Knockout of Erbin suppresses the proliferative potential of NRK-52E cells. The proliferation rate was assayed using CCK-8 kit. (G) Cells were incubated with EdU for 2 hours and stained. Dot plots show the corresponding quantification of EdU staining as EdU^{+}cells/total cells (%).Scale bar, 50 μm. (H and I) Knockout of Erbin sensitizes the NRK-52E cells to H_2O_2, hypoxia/re-oxygenation (H/R), and cisplatin-induced cellular senescence. Representative images of SA-β-gal staining (H), and EdU staining (I). Scale bar for SA-β-gal staining, 100 μm; Scale bar for EdU staining, 50 μm. Dot plots in (H) shows the relative fold change of SA-β-gal staining increase after treatment. Dot plots in (I) shows the fold change of EdU staining decrease after treatment. (J) The cell viability of NRK-52E-KO and NRK-52E-NC cells were measured after H_2O_2, H/R, and cisplatin treatment using CCK-8 assay. For H_2O_2 treatment, the cells were treated with various concentrations of H_2O_2 (250, 500, and 1000 μM) for 1 hour, changed with full medium, and continued to culture for 24 hours. For H/R treatment, the cells were incubated with four volumes of mineral oil on the top for various times (1, 2, and 3 hours), followed by extensive washing and culture in full medium for 24 hours. For cisplatin treatment, cells were cultured in the presence of various concentrations of cisplatin (2.5, 5, and 10 μM) for 24 hours. The above experiments were repeated three times. Results are presented as the mean ± s.e.m. Statistical analyses were conducted using two-tailed unpaired Student's t-test in B, G, H, I and J, and one-way ANOVA with Dunnett's multiple comparison test in F.

Appendix Figure S11

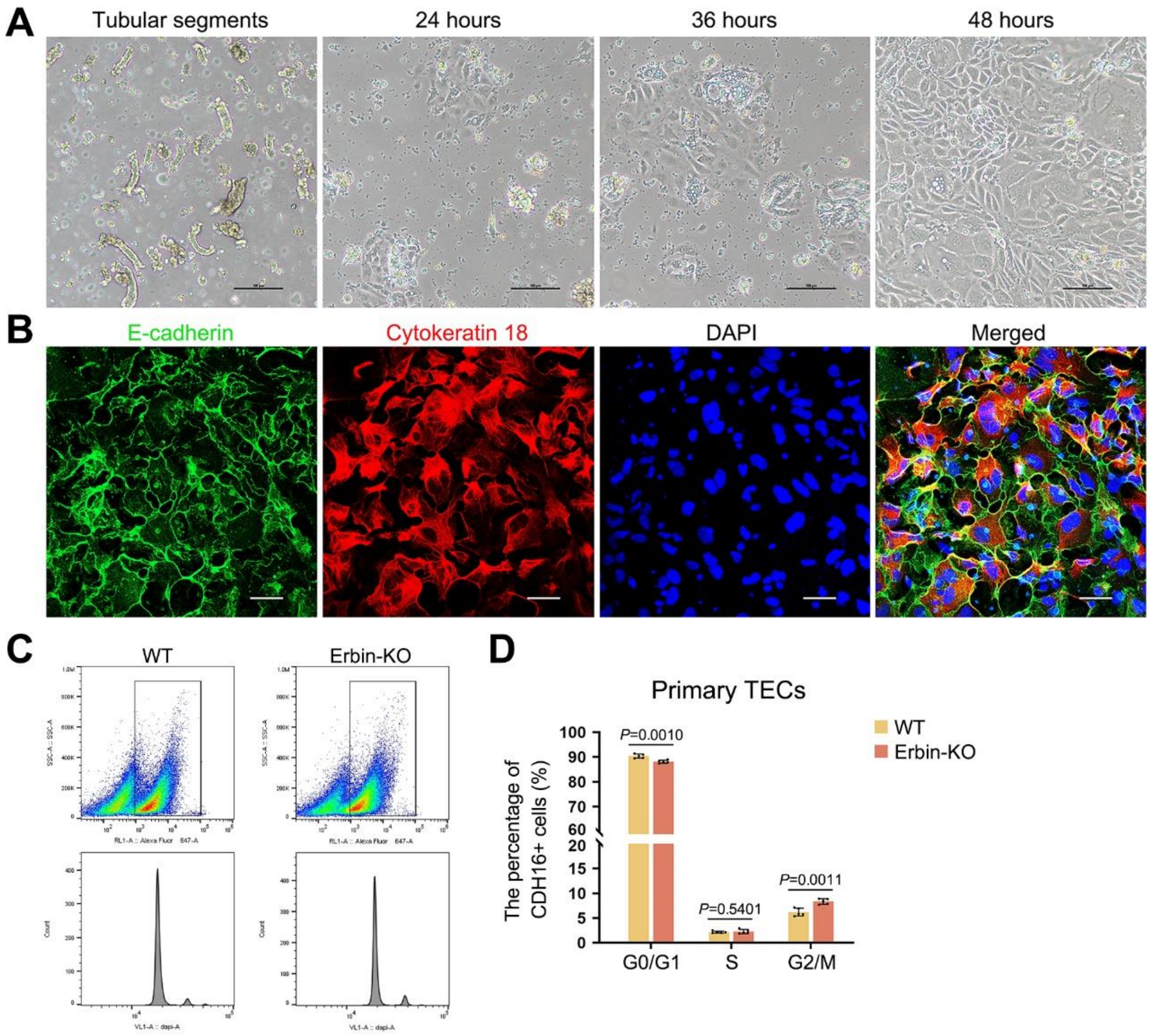

Appendix Figure S11. Identification of the primary renal tubular cells and cell cycle analysis by flow cytometry for the CDH16$^+$ TECs selected from the homogenate of Erbin knockout kidney. (A) Culture of the isolated primary tubular cells. Bar, 100 μm. (B) Representative images of dual immunofluorescence staining for the isolated primary TECs with cytokeratin 18 (red) and E-cadherin (green). Bar, 50 μm. (C) Flow cytometry analysis showing selection of CDH16$^+$ TECs from the homogenate of WT and Erbin knockout mice (top), and cell cycle analysis of the selected population (bottom). (D) The quantification of CDH16$^+$ TECs in different cell cycle phases for (C). The above experiments were repeated three times. Results are presented as the mean ± s.e.m. Statistical analyses were conducted using two-tailed unpaired Student's t-test.

Appendix Figure S12

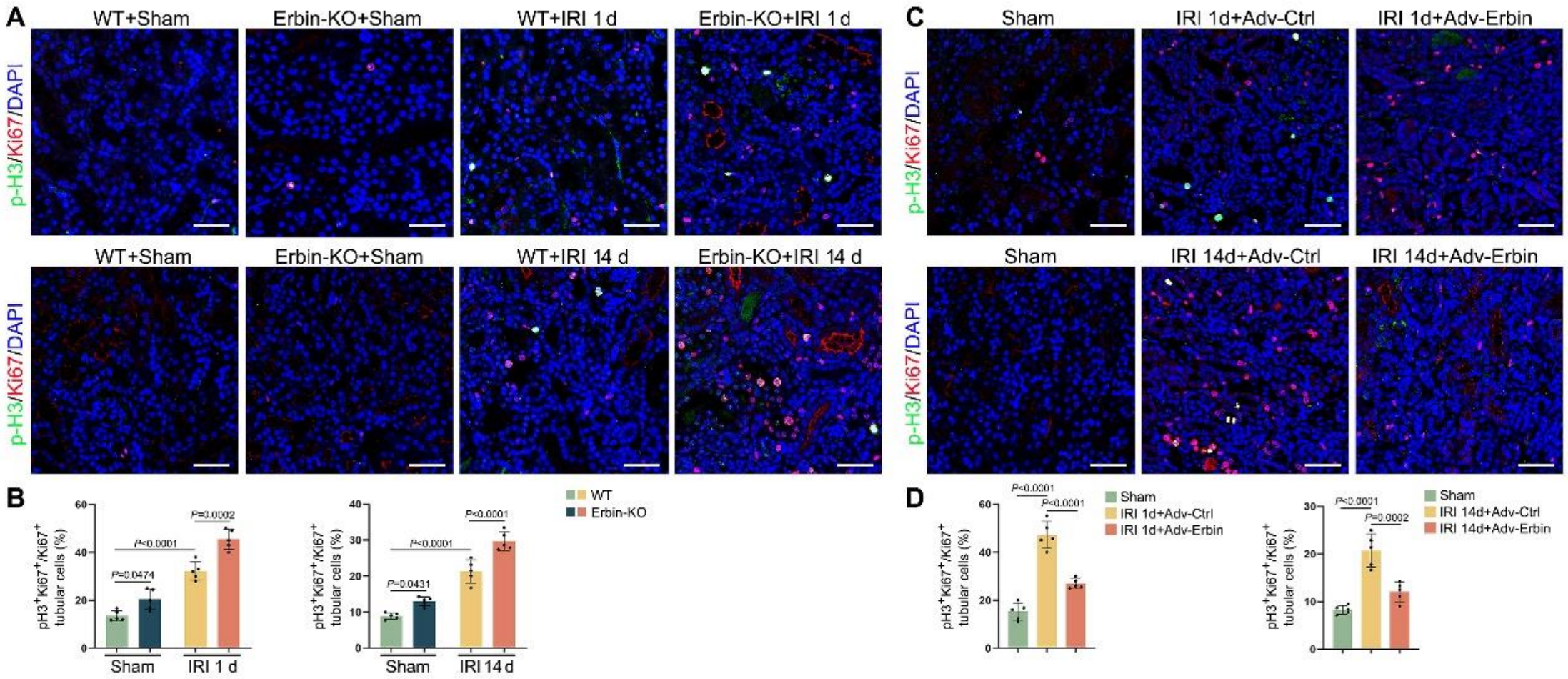

Appendix Figure S12. The dysregulation of Erbin is associated with the change of tubular cells in G2/M phase in mice. (A) Representative images of pH3/Ki67 dual immunostaining from kidneys of WT and Erbin knockout mice on day 1 and day 14 after I/R. Scale bar, 50 µm. n=5 per group. (B) The percentage of $pH3^+Ki67^+$ tubular cells relative to the proliferating $Ki67^+$ tubular cells for (A). (C) Representative images of pH3/Ki67 dual immunostaining from Adv-Erbin-injected kidneys on day 1 and day 14 after I/R. Scale bar, 50 µm. n=5 per group. (D) The percentage of $pH3^+Ki67^+$ tubular cells relative to the proliferating $Ki67^+$ tubular cells (C). Statistical analyses were conducted using one-way ANOVA with Tukey's multiple comparison test in B, and one-way ANOVA with Dunnett's multiple comparisons test in D.

Appendix Figure S13

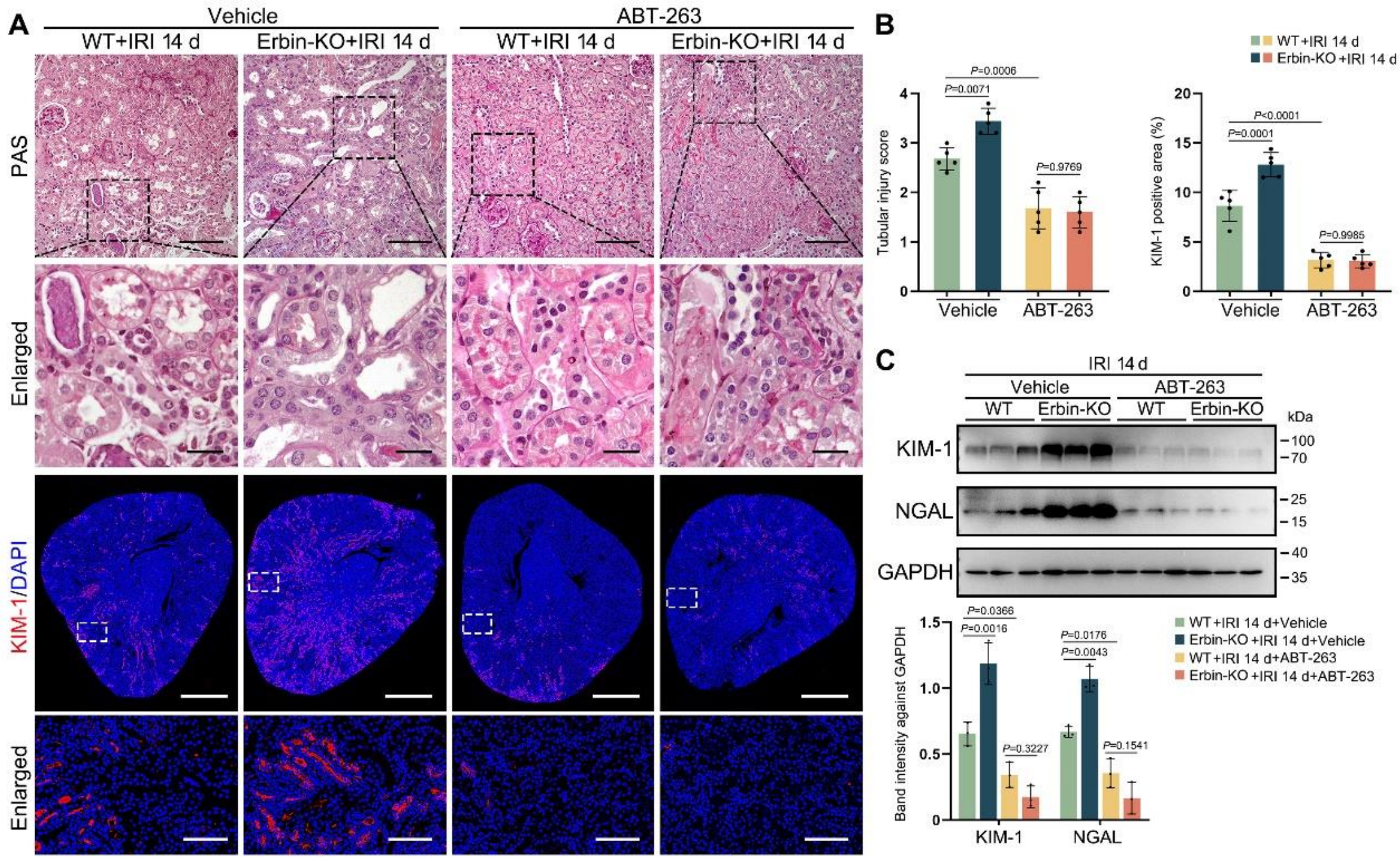

Appendix Figure S13. Senolytic treatment reduces tubular injury and abrogates the detrimental influences of Erbin deficiency on tubular architecture of the IRI kidney. ABT-263 was administered by gavage daily for 2 cycles, each of 4 days, from 1 day after I/R. Kidneys were harvested at 14 days after I/R (n=5 per group). (A) Representative images of PAS staining and KIM-1 immunostaining. Scale bar for PAS staining, 100 μm, and the boxed areas are enlarged (Scale bar, 25 μm). KIM-1 immunostaining images of whole kidney cross-sectional area are stitched at ×200 magnification (Scale bar, 1000 μm), and the bottom panels are enlarged from the boxed areas in the stitched images (scale bar, 100 μm). (B) Tubular injury score and quantification of KIM-1 staining in cortex and outer medulla from whole kidney section images. (C) KIM-1 and NGAL expression were examined by western blot. Statistical analyses were conducted using one-way ANOVA with Tukey's multiple comparison test.

Appendix Figure S14

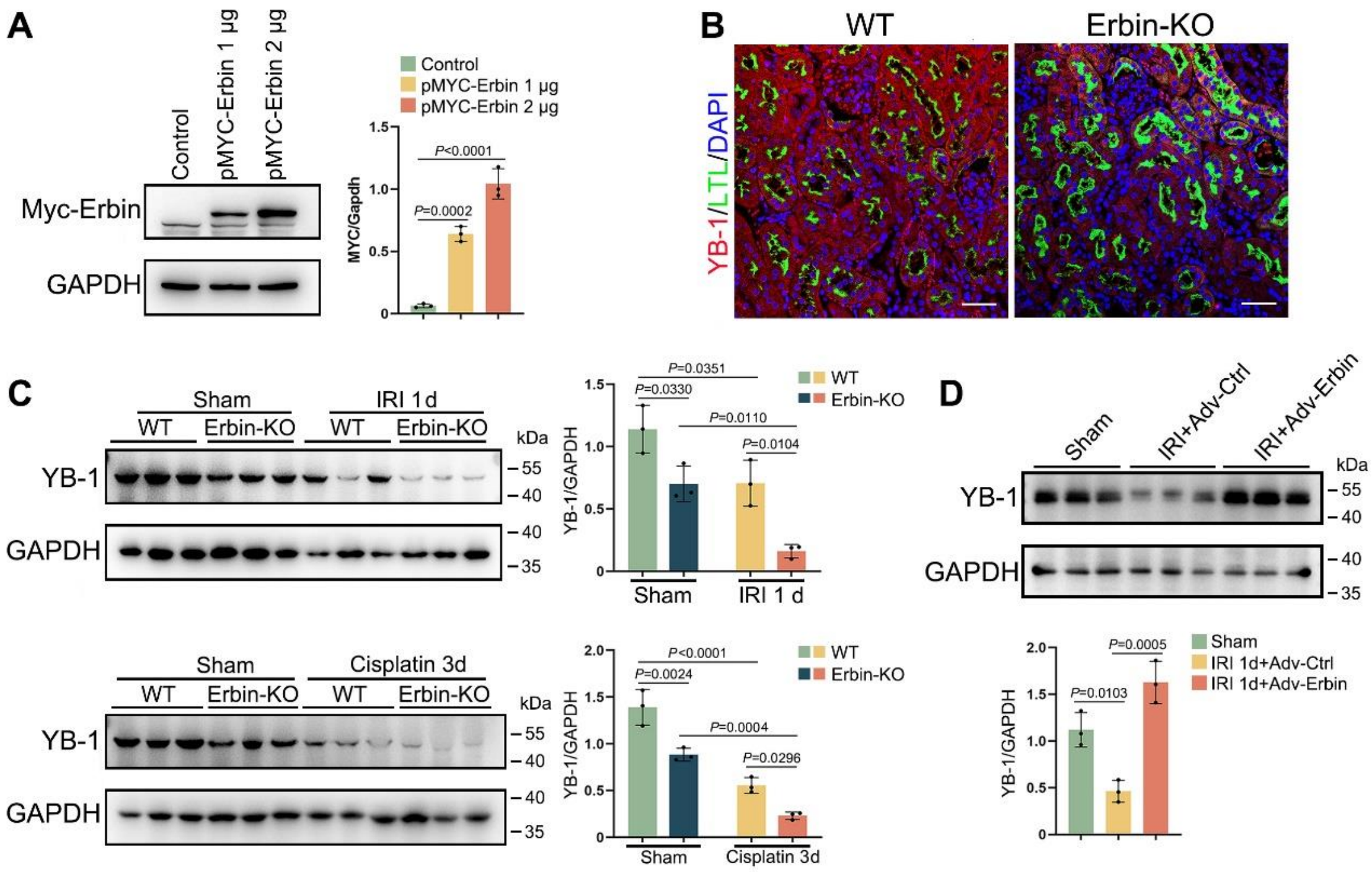

Appendix Figure S14. Erbin maintains the YB-1 protein level in mouse kidneys. (A) Overexpression of Myc-Erbin protein in NRK-52E cells. The cells were transfected with 1μg or 2 μg pMYC-Erbin plasmid and cultured for 48 hours. Myc-Erbin was determined by western blot. (B) Representative images of Erbin knockout kidney sections stained for YB-1 (red) and LTL (green). Scale bar, 50 μm. (C) The decrease of YB-1 was exacerbated in the IRI- or cisplatin-treated kidney. (D) Ectopic expression of Erbin greatly increased the amount of YB-1 protein in the IRI kidney. Results are presented as the mean ± s.e.m. Statistical analyses were conducted using one-way ANOVA with Dunnett's multiple comparison test in A, and one-way ANOVA with Tukey's multiple comparison test in C and D.

Appendix Figure S15

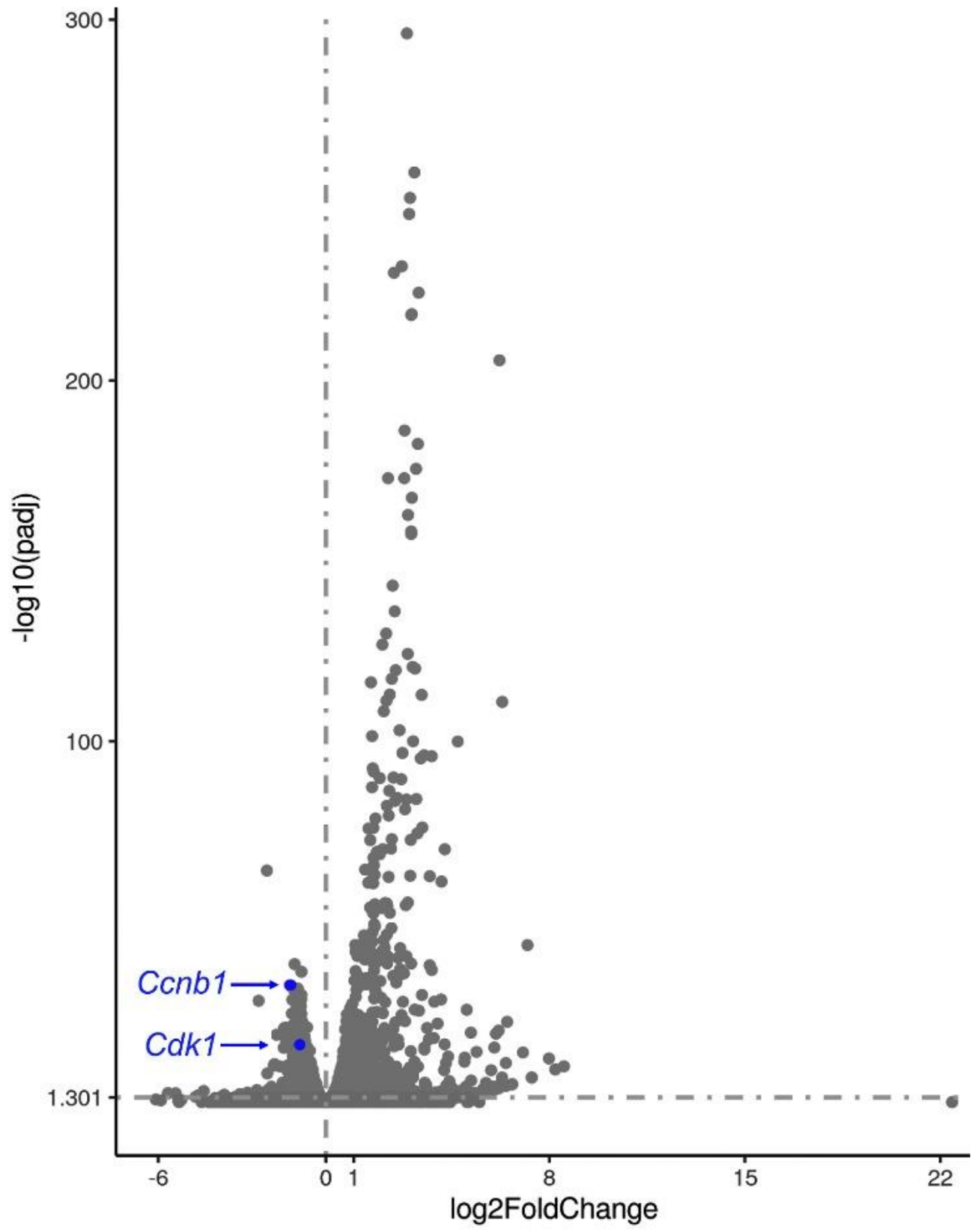

Appendix Figure S15. Volcano plot showing differentially expressed genes in the Erbin knockout NRK-52E cells versus control cell lines. The blue dots represent *Cdk1* and *Ccnb1* that are significantly downregulated in Erbin knockout NRK-52E cells.

Appendix Figure S16

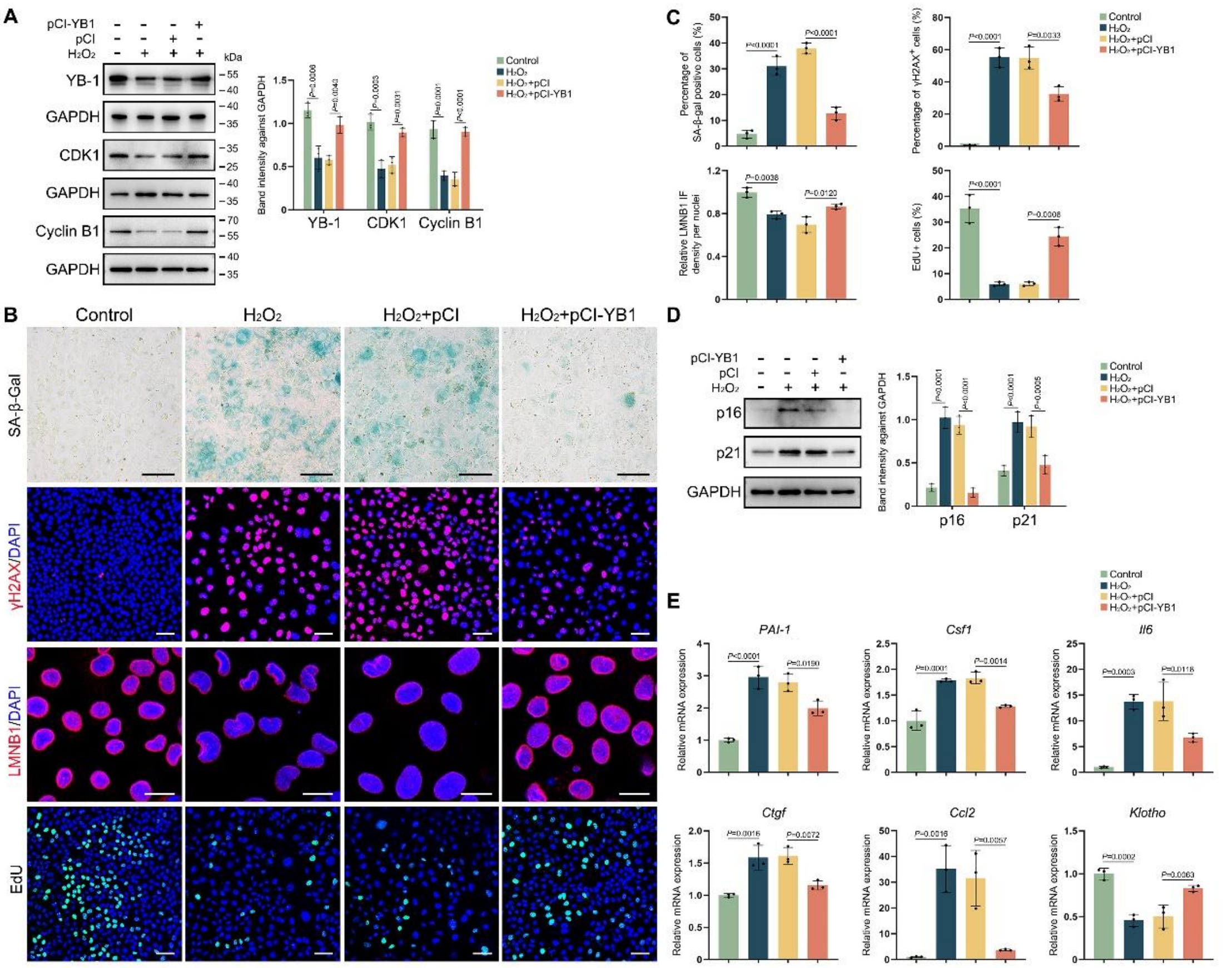

Appendix Figure S16. Ectopic expression of YB-1 prevents H_2O_2-induced cellular senescence in NRK-52E cells. NRK-52E cells were transfected with pCI-YB1 plasmid for 24 hours and then cultured with H_2O_2 at a final concentration of 250 μM for 48 hours. (A) YB-1, Cyclin B1 and CDK1 expression were examined by western blot. (B) Representative images of SA-β-gal staining, γH2AX, and LMNB1 immunostaining, and EdU staining. Scale bar for SA-β-gal staining, 100 μm; Scale bar for γH2AX and EdU staining, 50 μm; Scale bar for LMNB1 staining, 20 μm. (C) Quantification of SA-β-gal, γH2AX, LMNB1, and EdU staining. (D) Western blot analysis of p21 and p16. (E) YB-1 overexpression inhibited the increase of SASP markers in the H_2O_2-treated NRK-52E cells. The above experiments were repeated three times. Results are presented as the mean ± s.e.m. Statistical analyses were conducted using one-way ANOVA with Tukey's multiple comparison test.

Appendix Figure S17

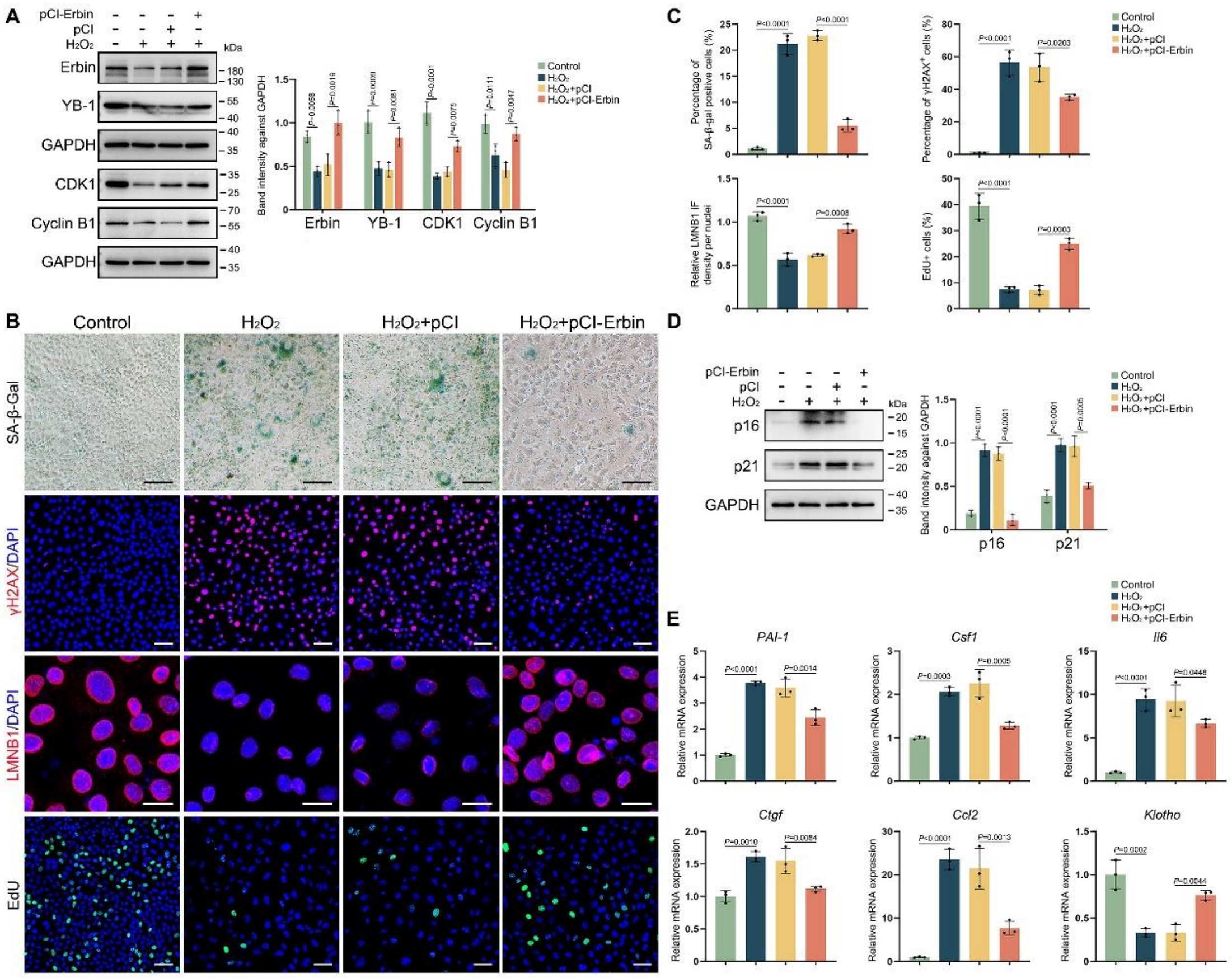

Appendix Figure S17. Ectopic expression of Erbin prevents H_2O_2-induced cellular senescence in NRK-52E cells. NRK-52E cells were transfected with pCI-Erbin plasmid for 24 hours and then cultured with H_2O_2 at a final concentration of 250 μM for 48 hours. (A) Erbin, YB-1, Cyclin B1 and CDK1 expression were examined by western blot. (B) Representative images of SA-β-gal staining, γH2AX, and LMNB1 immunostaining, and EdU staining. Scale bar for SA-β-gal staining, 100 μm; Scale bar for γH2AX and EdU staining, 50 μm; Scale bar for LMNB1 staining, 20 μm. (C) Quantification of SA-β-gal, γH2AX, LMNB1, and EdU staining. (D) Western blot analysis of p21 and p16. (E) Erbin overexpression inhibited the increase of SASP markers in the H_2O_2-treated NRK-52E cells. The above experiments were repeated three times. Results are presented as the mean ± s.e.m. Statistical analyses were conducted using one-way ANOVA with Tukey's multiple comparison test.

Appendix Figure S18

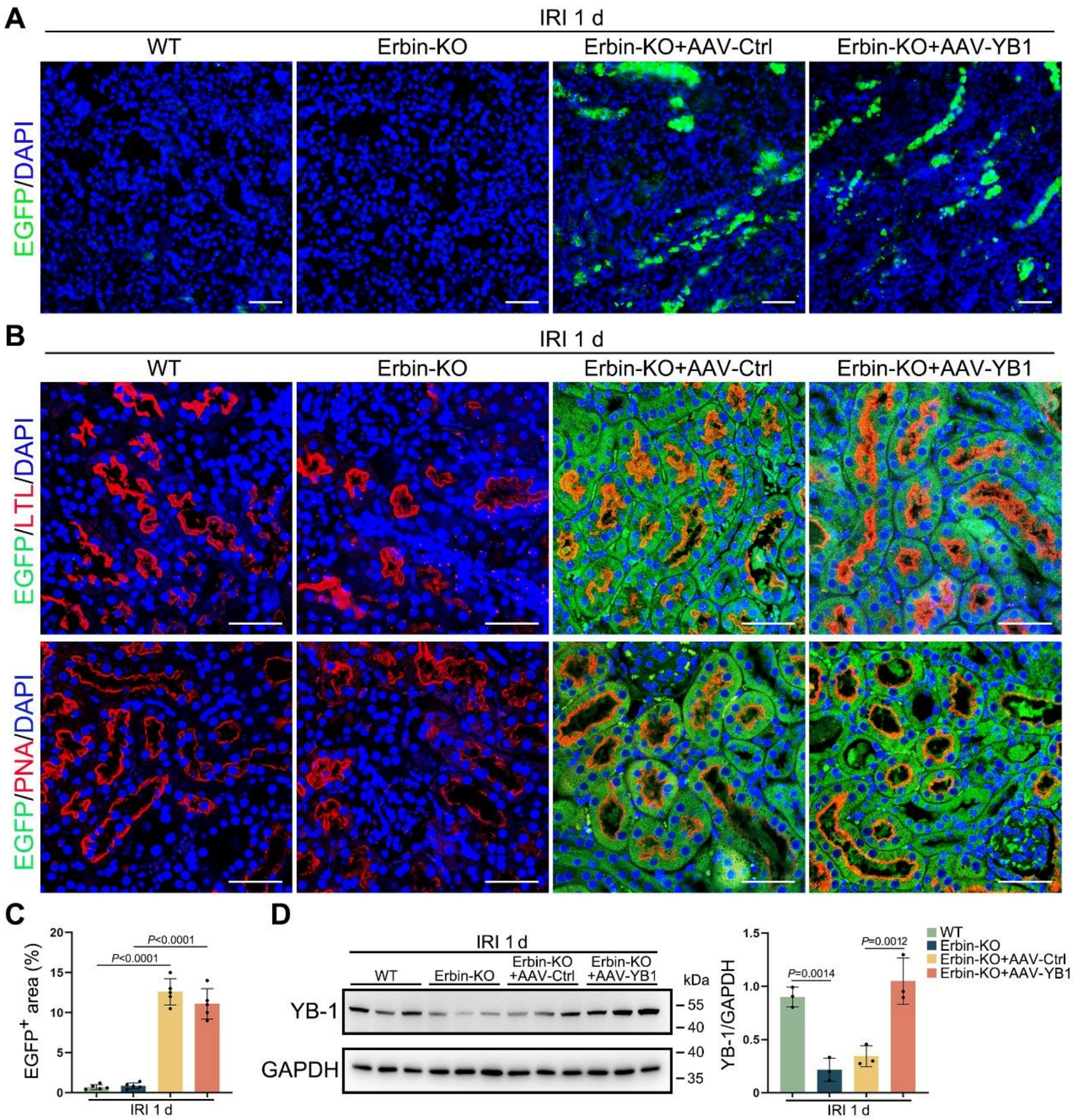

Appendix Figure S18. The distribution of AAV in the tubular cells of mice. (A) The efficiency of AAV infection in tubular cells was assessed using the immunofluorescence of co-expressing EGFP at 3 weeks after the *in situ* injection (n=5 per group). Scale bar, 50 μm. (B) EGFP expression was quantitatively assessed by measuring the immunofluorescence area. (C) Validation of the segment-specific distribution of AAV by co-staining EGFP with proximal tubular marker LTL, and distal tubular marker PNA respectively. Scale bar, 50 μm. (D) YB-1 expression in the cortex was examined by western blot. Results are presented as the mean ± s.e.m. Statistical analyses were

conducted using one-way ANOVA with Tukey's multiple comparison test.

Appendix Figure S19

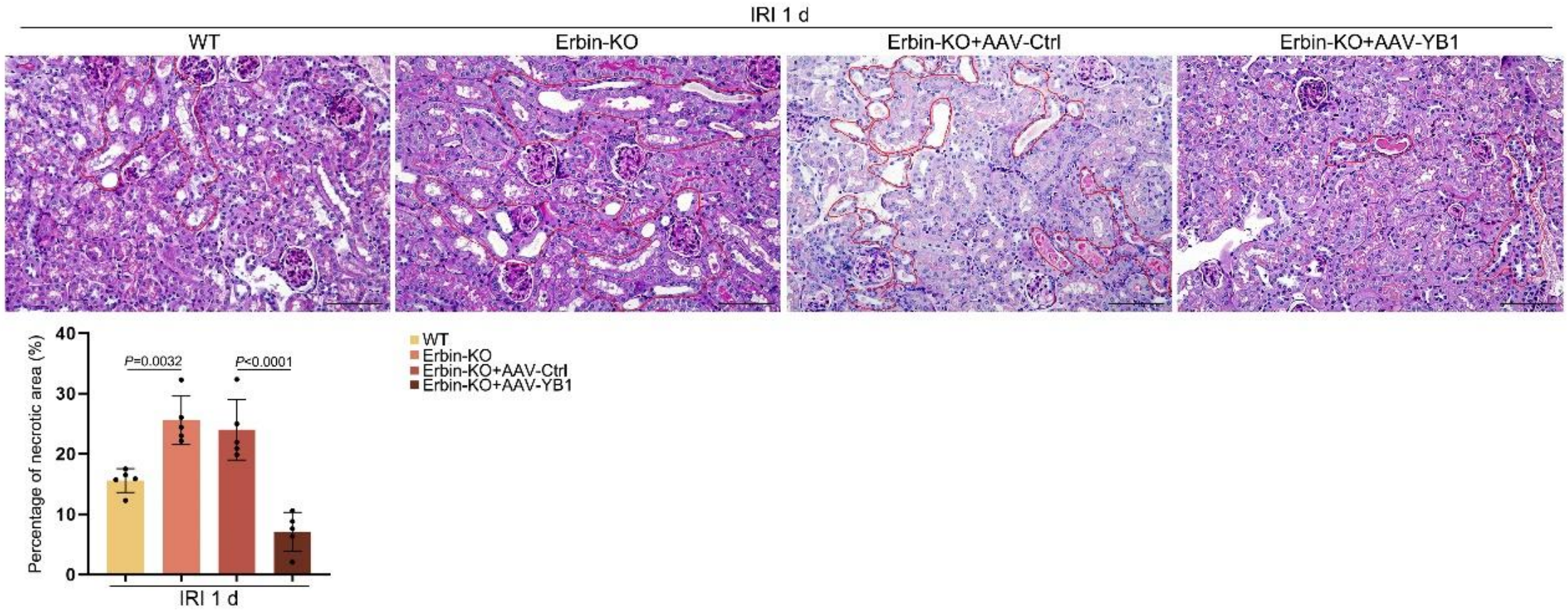

Appendix Figure 19. YB-1 Overexpression abolished the detrimental effect of Erbin deficiency on IRI-induced necrotic cell death in mice. The necrotic area, defined by a red line, was determined by PAS staining at one day after IRI, $n = 5$ for each group. Scale bar, 100 μm. Dot plot shows the percentage of necrotic area. Statistical analyses were conducted using one-way ANOVA with Tukey's multiple comparison test.

Appendix Figure S20

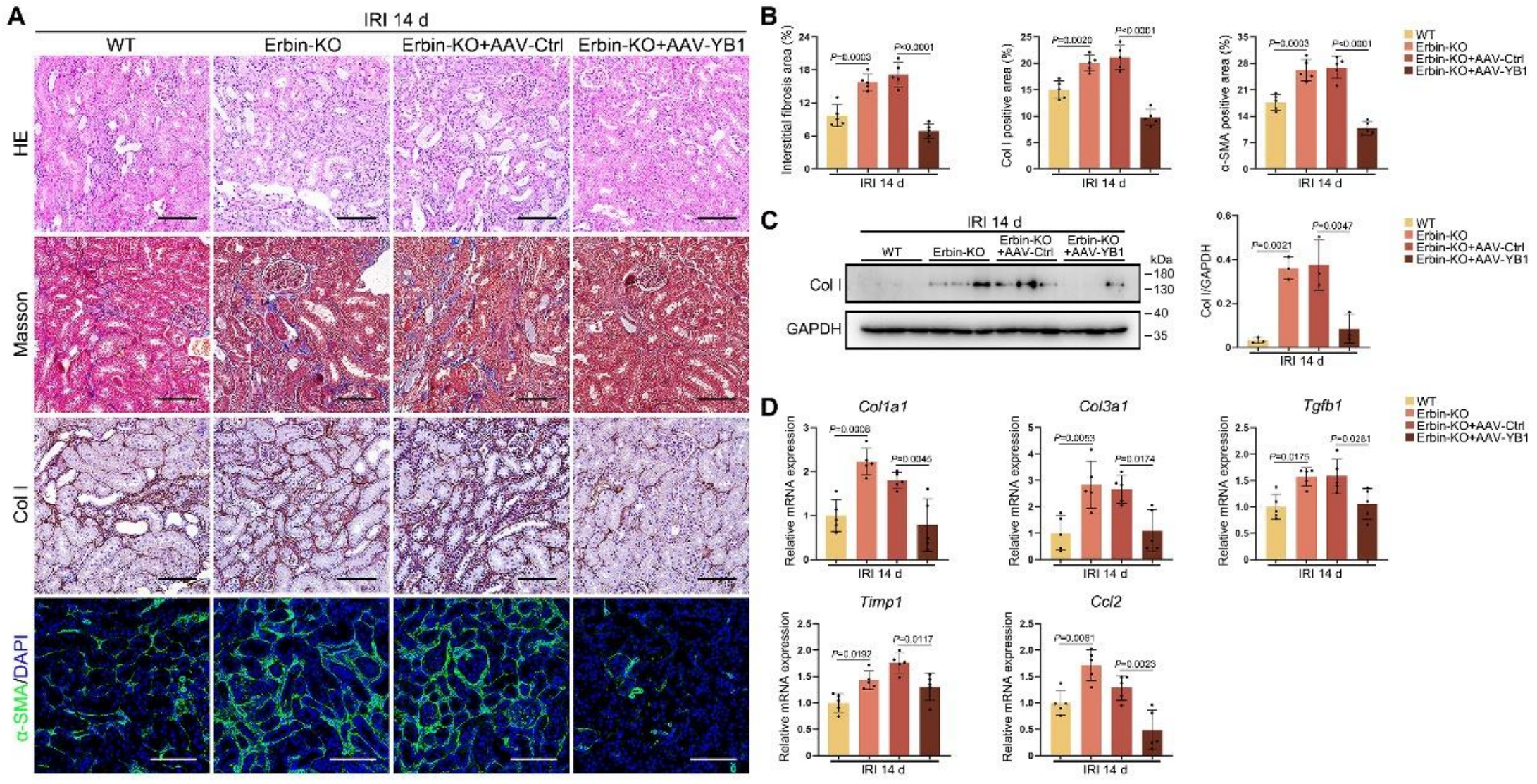

Appendix Figure 20. Overexpression of YB-1 abolished the detrimental effect of Erbin deficiency on IRI-induced interstitial fibrosis. The adeno-associated virus (AAV) was administered *in situ* in the cortex of the kidney 21 days before I/R. Kidneys were harvested at day 14 after I/R (n=5 per group). (A) Representative images of HE and Masson staining, and immunohistochemistry for collagen I and immunostaining for α-SMA in mouse kidneys. Scale bars, 100 μm. (B) Quantitative analysis of tubular interstitial fibrosis from Masson staining, and quantification of Col I and α-SMA staining area. (C) Kidney collagen I was detected by western blot. (D) The mRNA expression of *Col1a1*, *Col3a1*, *Tgfb1*, *Ccl2*, and *Timp1* were examined by qRT-PCR. The results are shown as fold change compared to WT mice subjected to IRI. Results are presented as the mean ± s.e.m. Statistical analyses were conducted using one-way ANOVA with Tukey's multiple comparison test.

Appendix Figure S21

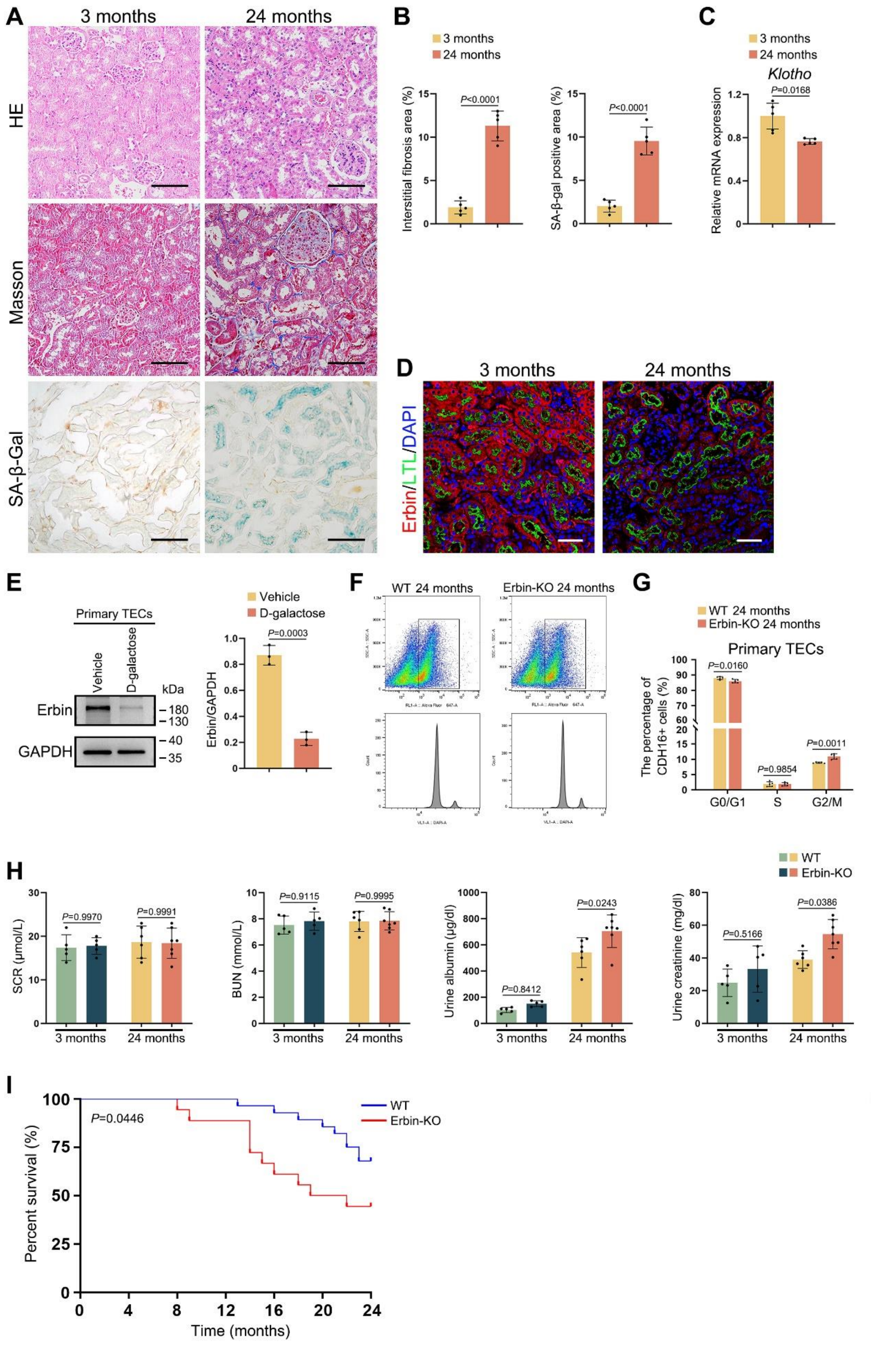

Appendix Figure S21. The loss of Erbin increases the proportion of G2/M phase in primary TECs

and affects the lifespan. (A) Representative images of HE, Masson, and SA-β-gal staining for mouse kidneys at 3 or 24 months old (n=5 per group). Scale bars, 100 μm. (B) Quantitative analysis of tubular interstitial fibrosis from Masson staining and the quantification of SA-β-gal staining. (C) Kidney Klotho expression was examined by qRT-PCR. The results are shown as fold change relative to the mouse at 3 months old. (D) Representative images of Erbin and LTL costaining for kidneys from mice aged 3 or 24 months. Scale bars, 50 μm. (E) Erbin is decreased in the D-galactose-treated primary TECs. The primary TECs were isolated from mice at the age of 3 months, and incubated with D-galactose at a final concentration of 200 mM for 4 days. (F) Flow cytometry analysis showing selection of $CDH16^+$ TECs from the homogenate of WT and Erbin knockout mice at the age of 24 months (top), and cell cycle analysis of the selected population (bottom). (G) The quantification of $CDH16^+$ TECs in different cell cycle phases for (f). (H) Analysis of SCR, BUN, urinary albumin and urinary creatinine in Erbin deficient and WT mice at the age of 3 and 24 months (n=5 for 3-month-old WT and Erbin-KO mice, n=6 for 24-month-old WT mice, and n=7 for 24-month-old Erbin-KO mice). (I) Survival curves in Erbin deficient (n=18) and WT (n=28) mice. Results are presented as the mean ± s.e.m. Statistical analyses were conducted using two-tailed unpaired Student's t-test with Welch's correction in B, C, E and G, Brown-Forsythe and Welch ANOVA with Tamhane's multiple comparisons test in H, and the log-rank test in I.
