# Supplementary material for: Loss of Erbin in tubular epithelial cells sensitizes mice to acute kidney injury and aggravates renal fibrosis
Source: EMBO J. 2026 Aug 24;45(19):6782–812. doi: 10.1038/s44318-026-00885-y (PMC13631325; doi:10.1038/s44318-026-00885-y)
Supplement: Supplementary file 3 [file 44318_2026_885_MOESM3_ESM.pdf]

THE EMBO JOURNAL

Loss of Erbin in tubular epithelial cells sensitizes mice to acute kidney injury and aggravates renal fibrosis

Fang Shao, Zhengrong Ren, Lei Shi, Yanyan Sun, Wen Sun, Jing Tang, Yingsailamu Yingsailamu, Zhi Ding, Junfeng Zhang, and Yuhui Zang

Corresponding authors: Yuhui Zang (zangyh@nju.edu.cn) , Junfeng Zhang (jfzhang@nju.edu.cn)

Review Timeline:

Submission Date:	20th Apr 26
Editorial Decision:	19th Jun 26
Editor's Correspondence:	25th Jun 26
Revision Received:	14th Jul 26
Accepted:	23rd Jul 26

Editor: Daniel Klimmeck

Transaction Report:

This manuscript was previously reviewed at another journal. As EMBO Press has a transfer agreement (including the identities of the referees) with that journal, revision was invited based on the reports from that previous external submission.

(Note: With the exception of the correction of typographical or spelling errors that could be a source of ambiguity, letters and reports are not edited. Depending on transfer agreements, referee reports obtained elsewhere may or may not be included in this compilation. Referee reports are anonymous unless the Referee chooses to sign their reports.)

Reviewers' Comments:

Reviewer #1 (Remarks to the Author):

The authors have addressed the majority of the reviewers' concerns appropriately in the revised manuscript, and overall, the responses and additional data are satisfactory. I believe the manuscript has improved substantially. I have only a few remaining concerns that I would like the authors to consider.

Response: We thank the reviewer for the positive evaluation of our efforts in revision.

Major Point 5

In humans, a decline in eGFR is closely associated with increased mortality, primarily through elevated risks of cardiovascular disease and infection. However, mice do not typically develop cardiovascular disease or infection to the same extent as humans. Therefore, it is unlikely that a modest reduction in eGFR alone would lead to significantly shortened lifespan in mice. In addition, lifespan analysis generally requires a large sample size to ensure adequate statistical power. Presenting survival curves based on approximately 20 animals as a main figure may have statistical limitations. For these reasons, it may be more appropriate to either remove the lifespan data from the main figures or move them to supplementary information. Conversely, if tubule-specific deletion of Erbin truly influences lifespan, this would be an important finding and warrants further detailed investigation in future studies.

The current description of eGFR may be misunderstood as applying a human eGFR equation. Because the creatinine-urea-based estimated GFR used in this study is not a widely standardized method in mice, it would be advisable to use more cautious terminology, such as "creatinine-urea-based estimated GFR," to avoid misinterpretation.

Response: We thank the reviewer for the valuable suggestion. As suggested by the reviewer, we have moved the lifespan data from the main figures to Supplementary Figure 21i, and removed the eGFR data from the manuscript.

Minor Point 1

In Figure 7p, the authors present increases in urinary albumin, urinary creatinine, and UACR as markers of renal dysfunction. However, both urinary albumin and urinary creatinine can vary

substantially depending on urine concentration. For this reason, UACR alone would be sufficient, and presenting urinary albumin or creatinine individually may not be necessary.

Response: We thank the reviewer for the valuable suggestion. As suggested by the reviewer, we have moved the urinary albumin and urinary creatinine data from the main figures to supplementary Figure 21h, and only present UACR in Figure 7p in the revised manuscript

Reviewer #2 (Remarks to the Author):

Overall assessment

Multiple initial concerns were addressed through additional experiments, including necrosis quantification, inclusion of additional senescence markers, assessment of cell-cycle states at day 14, and post-injury Erbin overexpression…. Overall, the authors present convincing evidence that Erbin has a protective role in the kidney. However, some results and interpretations remain unclear

Response: We thank the reviewer for the positive evaluation of our revised manuscript, and deeply value your insightful comments, which has inspired us to refine our manuscript for greater clarity and accuracy.

Responses to comments 3 and 4

While the authors now acknowledge that the acute phenotype observed at day 1 after ischemia–reperfusion injury (IRI) is unlikely to be senescence-driven and have revised the manuscript accordingly, their response still attributes the increased injury at early time points primarily to impaired or altered tubular cell cycle progression. Specifically, they suggest that Erbin deficiency compromises the accelerated proliferative response of surviving tubular epithelial cells.

I remain unconvinced that this explanation fully accounts for the early phenotype, particularly during the acute phase (12–24 hours after injury). At these early time points, the dominant biological processes following IRI are well known to be cellular damage and tubular cell death, rather than failure of compensatory proliferation, which typically becomes relevant at later stages. Although accelerated cell-cycle entry of surviving tubular cells is an important component of adaptive repair, it is unlikely to be the major determinant of injury severity at day 1.

Importantly, the authors now provide evidence of increased necrotic cell death in Erbin-deficient kidneys at early time points. This finding supports the interpretation that enhanced tubular cell death,

rather than impaired proliferation per se, may represent a primary driver of the worsened acute injury. Increased cell death at this stage could secondarily influence later outcomes, including altered proliferation dynamics, increased senescence burden, and ultimately more severe fibrosis.

Response: We thank the reviewer for the insightful comment, and for highlighting the need for rational interpretation regarding the possible mechanisms underlying the aggravated early tubular injury in Erbin-deficient mice after ischemia-reperfusion injury (IRI). We agree with the reviewer that during the initial 12-24 hours after IRI, the primary pathological processes are cellular damage and tubular cell death, rather than a failure of compensatory proliferation. As indicated by the response to the next comment, we have now performed new *in vitro* experiments as suggested in this revision, and showed that Erbin knockout tubular cells exhibit an increased susceptibility to H_2O_2-, cisplatin- and hypoxia-induced cell death *in vitro* (Supplementary Figure S10j in the revised manuscript). In this study, we obtained the tubule-specific Erbin-deficient mice by crossing the Ksp-Cre and Erbin$^{flox/flox}$ mice, and subjected them to IRI. In line with the *in vitro* observations, the inherent deficiency of Erbin might render the tubular cells an increased susceptibility to the alternative forms of cell death in the acute phase of kidney injury, therefore, suggesting a causal association between Erbin deficiency and the aggravated tubular damage in response to AKI.

Given these data, as well as the insightful comment by the reviewer, we acknowledge that our previous interpretation concerning the increased tubular injury in Erbin-deficient mice at early stage of AKI is not appropriate, and hope that these amendments will help to clarify the inaccuracy and ambiguity.

Responses to comment 5

The authors' explanation that enrichment of the GO term "regulation of cell death" among upregulated DEGs in Erbin-deficient cells is most likely a secondary consequence of cell cycle arrest and cellular senescence is not convincing. Enrichment of cell-death–related pathways in transcriptomic analyses does not necessarily imply that cells are actively dying at baseline. Rather, it often reflects altered stress responses and increased susceptibility to death upon subsequent insults, such as oxidative stress or DNA damage.

Importantly, changes in the expression of cell-death–related genes do not require overt cell death to be occurring; instead, they may lower the threshold for activation of apoptotic, necrotic, or

pyroptotic pathways following injury. In this context, the fact that Erbin knockout cells are viable under basal conditions does not exclude a biologically meaningful role for Erbin in regulating injury-induced cell death.

While the authors argue that a detailed analysis of cell death mechanisms is beyond the scope of the present study, it is important to emphasize that their own in vivo data already provide clear evidence that Erbin loss affects tubular cell death following injury. Specifically, the increased necrotic cell death observed in Erbin-deficient kidneys at early time points after IRI directly supports a functional role of Erbin in protecting tubular epithelial cells from injury-induced death. Therefore, the involvement of cell death pathways is not speculative but represents an intrinsic component of the phenotype documented in this manuscript. In this light, dismissing the enrichment of "regulation of cell death" genes as merely a downstream consequence of cell cycle arrest or senescence appears insufficiently justified.

Also, the conclusion that "deletion of Erbin sensitized the tubular cells to insults-induced cellular senescence" based on the β-galactosidase staining shown in Supplementary Fig. S10h and I is not very convincing. Upon closer inspection, the increase in β-gal–positive cells following treatment with H_2O_2, cisplatin, or hypoxia/reoxygenation appears modest and not substantially different from the baseline β-gal positivity already observed in untreated Erbin-deficient cells in Figure 4e.

Moreover, the comparisons presented in Supplementary Fig. S10 primarily contrast Erbin-deficient cells with control cells within the same treatment condition. They do not clearly demonstrate that the magnitude of senescence induction by the insult itself is greater in Erbin-deficient cells than in control cells. To convincingly demonstrate sensitization, the appropriate comparisons should include:

-WT versus WT treated cells, to quantify the senescence induced by each insult in control cells;

-Erbin-KO versus Erbin-KO treated cells, to quantify the senescence induced in the absence of Erbin;

followed by a direct comparison of the treatment-induced increase (or normalized change relative to untreated baseline) between WT and Erbin-KO cells.

Without showing that the same insult induces a larger increase in senescence in Erbin-deficient cells compared with WT cells, it is difficult to conclude that Erbin loss specifically sensitizes tubular cells to insult-induced senescence, rather than simply increasing baseline senescence levels.

The increase in β-gal–positive cells after treatment with H_2O_2, cisplatin, or hypoxia/reoxygenation

is weak. This raises the question of whether the doses used were sufficient to robustly challenge the cells. It is possible that higher doses were avoided because they caused significant cell death; however, this cannot be evaluated, as no cell viability or cell death measurements are provided for these conditions.

As previously requested, inclusion of cell viability or death assays would have been straightforward and would greatly strengthen the interpretation. Ideally, viability analyses should be performed across multiple doses of each insult, in parallel with senescence measurements, to determine whether Erbin deletion shifts the balance between survival, senescence, and cell death.

Response: We thank the reviewer for bringing this concern to our attention. In our study, the enrichment of genes related to "DNA replication" and "chromosome organization" were identified as the top GO terms in the downregulated DEGs after Erbin knockout in tubular cells. Meanwhile, KEGG analysis for downregulated DEGs characterized "cell cycle", "DNA replication" and multiple metabolism pathways, including "fatty acid metabolism" and "oxidative phosphorylation", as the most significantly enriched pathways in Erbin deficient cells. Gene set enrichment analysis (GSEA) further showed that the pathways associated with cellular senescence remained elevated, whereas the DNA replication pathway was inhibited after Erbin deletion. More importantly, multiple experimental evidence indicate that loss of Erbin prolongs the G2/M phase, tips the balance from proliferation to cell cycle arrest, and induces the biomarkers of senescence. Given these observations, we concluded that Erbin deficiency would lead to senescence in tubular cells.

Regarding the term "regulation of cell death", we appreciate the concern raised by the reviewer, and agree that attributing enrichment of the GO term "regulation of cell death" to a secondary consequence of cellular senescence is not appropriate. Nevertheless, it's well established that senescent cells are a heterogeneous collection of cells with multifaceted features. Marked cell-to-cell gene expression variability exists in the senescent cell cultures *in vitro*, and, therefore, complicates the comprehensive identification of senescent cells [R1, R2]. Meanwhile, accumulating evidence indicate that the senescence and cell death signaling pathways are tightly interconnected [R3, R4]. For instance, the age-associated increases in the upstream and downstream signals of regulated cell death, including apoptosis, necroptosis, pyroptosis, and ferroptosis, are closely related to the induction of cellular senescence and its phenotype [R5]. Cells may undergo either death or senescence in response to stress, contingent on the magnitude of damage and the specific cellular

context [R6, R7]. Taken these into account, the enrichments of both cellular senescence-related and cell death-related terms in the DEGs of Erbin-deficient tubular cells are not out of expectation. Finally, we would also like to point out that we did notice the enrichment of GO term “regulation of cell death” in the upregulated DEGs, and described this observation when preparing the manuscript. We have now supplemented the comments that highlight the possible involvement of Erbin loss in regulating tubular cell death during AKI in the discussion section of the revised manuscript. We hope that these amendments could help to clarify the raised concern.

With respect to the SA-β-galactosidase staining results in Supplementary Fig. S10 h and i, we have now performed new experiments as suggested by the reviewer. Briefly, we subjected the Erbin deficient and control cells to H_2O_2, cisplatin, and hypoxia/reoxygenation, and directly compared the insult-induced increases of SA-β-galactosidase staining in Erbin deficient cells with those of the control cells respectively. The quantitative results have been presented as the fold change of insults-induced increase of SA-β-gal staining between NRK-52E-KO and NRK-52E-NC cells as required (Supplementary Figure S10h). Similarly, direct comparisons of the insult-induced decrease of EdU staining have also been summarized in Supplementary Figure S10i in the revised manuscript. Collectively, these data suggest that loss of Erbin sensitizes the tubular cells to the induction of senescence *in vitro*.

Regarding the modest increase of SA-β-gal staining when comparing the insult-treated Erbin-deficient cells in Supplementary Figure S10h to the untreated cells in Figure 4e, we would like to emphasize that the data of Figure 4e were obtained in 2023, whereas the experiments of Supplementary Figure S10h were carried out during last revision in 2025. The variation of cell culture conditions, as well as alternative experimental elements, might systematically affect the outcome of staining. We have now presented the SA-β-gal staining for Erbin-deficient and WT control cells with or without insult treatment simultaneously within the same condition, and showed a significant increase of insult-induced SA-β-gal staining in Erbin knockout cells.

Finally, we have now performed cell viability assay across multiple doses of insults as required. In comparison with the control cells, the cell viability of Erbin deficient tubular cells was further inhibited when they were challenged to H_2O_2, cisplatin, and hypoxia/reoxygenation. These results have been summarized in Supplementary Figure S10j.

ABT-263 experiments

Finally, according to the authors' responses to previous comments, their proposed model suggests that loss of Erbin leads to persistent G2/M cell cycle arrest (or prolongation), which limits tubular cell repopulation and drives maladaptive repair and fibrosis. While this model is plausible, it becomes less consistent when considering the senolytic ABT-263 data.

If the primary mechanism underlying the phenotype were insufficient tubular repopulation due to cell cycle arrest, elimination of senescent cells by ABT-263 would not necessarily be expected to restore repair, and could even be detrimental if arrested-but-viable cells required for regeneration were removed. Instead, the observation that ABT-263 treatment alleviates the pathological phenotype is more compatible with a model in which senescent cells contribute mainly through SASP-mediated inflammatory and profibrotic signaling, and their clearance improves the tissue microenvironment despite persistent defects in cell cycle progression.

Alternatively, it is also possible that ABT-263 targets additional senescent or stress-responsive cell populations beyond tubular epithelial cells, such as interstitial fibroblasts, endothelial cells, or inflammatory cells, which could independently influence fibrosis and tissue remodeling.

Response: We thank the reviewer for bringing this concern to our attention. We agree with the reviewer that senescent tubular cells actively contribute to interstitial fibrosis through SASP-mediated inflammatory and profibrotic signaling. In this study, we found that the number of senescent tubular cells was greatly augmented in the IRI-induced fibrotic kidney from Ebin deficient mice in comparison with the counterpart of WT. Moreover, ectopic expression of Erbin after IRI operation markedly ameliorated tubular senescence, and reduced the severity of renal fibrosis. Together, these data suggest a detrimental role of tubular cell senescence in the development of kidney fibrosis. Given these observations, the ABT-263 experiment was designed to investigate whether deleting the senescent tubular cells will abolish the detrimental effect of Erbin deficiency on aggravating interstitial fibrosis. To this end, we chose to administrate ABT-263 from day 1 after IRI surgery, showing that the senolytic treatment abrogated the detrimental effect of Erbin deficiency on aggravating IRI-induced interstitial fibrosis.

We acknowledge that there are ambiguous and inconsistent meaning in the first sentence under the subtitle "The detrimental role of Erbin loss in renal fibrosis is associated with tubular senescence"

in page 9 of the manuscript. This vague statement might lead to misunderstanding the purpose and the hypothesized mechanism concerning ABT-263 experiments. We have now revised the sentence from "The accumulation of senescent TECs impedes adaptive repair after kidney injury and promotes the following interstitial fibrosis" to "The accumulation of senescent TECs promotes progressive fibrosis and impedes adaptive repair after kidney injury" in the revised manuscript. In addition, we refined the description of ABT-263 treatment as following:

"Administration of ABT-263 from day 1 after I/R abolished the detrimental effect of Erbin deficiency on tubular senescence, and abrogated the exacerbation of IRI-induced interstitial fibrosis in the Erbin deficient kidney (Extended Data Fig. 3). Meanwhile, ABT-263 treatment inhibited the increase of KIM-1 and NGAL, and improved the deteriorated tubular architecture in the IRI kidney (Supplementary Fig. S13)."

Finally, previous studies have shown that depletion of the senescent tubular cells benefit tubular repair in the murine models of kidney fibrosis. Nevertheless, how tubular cell regeneration is restored following the elimination of senescent cells remains largely elusive, though there are some speculated mechanisms [R8-R10].

With respect to whether elimination of senescent cells by ABT-263 would be beneficial or detrimental to tubular repair and regeneration, we think that it depends on the timing of senolytic treatment. As indicated by the reviewer in the first version of comments, senescent tubular cells may have some beneficial effects in the early stages of kidney injury, while their detrimental impact, particularly in promoting renal fibrosis, becomes more prominent at later stages. Thus, the timing of senolytic treatment may potentially affect the outcome. Previous studies showed that the post injury clearance of senescent tubular cells with ABT-263 inhibited progressive renal fibrosis and improved tubular repair in the cisplatin- and IRI-induced murine models of kidney fibrosis [R8, R9]. In consistent with these observations, we found that administration of ABT-263 from day 1 after IRI surgery abolished the detrimental effect of Erbin deficiency on tubular senescence, and improved the deteriorated tubular architecture in the IRI kidneys.

Regarding the possibility that ABT-263 targets other types of senescent cell populations beyond tubular epithelial cells, and thus contributes to the inhibitory effects, we think it is less likely. It is

established that the senescence was induced primarily in renal tubular epithelium in murine models of aging and the progression of kidney injury as well [R10, R11]. Meanwhile, the renal epithelial cell senescence correlates most strongly with tubular atrophy, progressive fibrosis, and functional loss in various animal models and human biopsy samples [R12-R14]. As a consequence, the accumulation of senescent tubular cells are considered a major source of profibrotic cytokine production, and, therefore, the key determinants of renal fibrosis in comparison with other types of senescent cells. Accordingly, previous reports have shown that depletion of senescent tubular cells protects against renal fibrosis. Herein, we observed the exacerbation of tubular cell senescence in the tubule-specific Erbin null mice subjected to IRI. Multiple *in vivo* data further showed that the increase of tubular cell senescence is closely associated the worsened renal fibrosis in the Erbin deficient mice. Together, it should be convincing to attribute the effect of ABT-263 to the depletion of senescent tubular cells, though we should not ignore the potential importance of senescence in other cell types.

References

[R1] Wiley, C. D. *et al*. Analysis of individual cells identifies cell-to-cell variability following induction of cellular senescence. *Aging Cell* **16**, 1043–1050 (2017).

[R2] Tang, H. *et al*. Single senescent cell sequencing reveals heterogeneity in senescent cells induced by telomere erosion. *Protein Cell* **10**, 370–375 (2019).

[R3] D'Arcy, M. S. Cell death: a review of the major forms of apoptosis, necrosis and autophagy, *Cell Biol. Int* **43**, 582–592 (2019).

[R4] Tower, J. Programmed cell death in aging, *Ageing Res Rev.* **23 (Pt A),** 90–100 (2015).

[R5] Shimizu, K. Inuzuka, H. & Tokunaga, F. The interplay between cell death and senescence in cancer. *Seminars in Cancer Biology* **108** 1–16 (2025).

[R6] Campisi, J, & d'Adda di Fagagna, F. Cellular senescence: when bad things happen to good cells, *Nat. Rev. Mol. Cell Biol.* **8**, 729–740 (2007).

[R7] Nehme, J. *et al*. Converting cell death into senescence by PARP1 inhibition improves recovery from acute oxidative injury. *Nat Aging* **4**, 771-782 (2024).

[R8] Li, S. *et al*. Tubular cell senescence promotes maladaptive kidney repair and chronic kidney disease after cisplatin nephrotoxicity. *JCI Insight* **8**, e166643 (2023).

[R9] O'Sullivan, E. D. *et al*. Single-cell analysis of senescent epithelia reveals targetable mechanisms promoting fibrosis. *JCI Insight*. **7**, e154124 (2022).

[R10] Mylonas, K. J. *et al*. Cellular senescence inhibits renal regeneration after injury in mice, with senolytic treatment promoting repair. *Sci Transl Med* **13,** eabb0203 (2021).

[R11] Jin, H. *et al*. Epithelial innate immunity mediates tubular cell senescence after kidney injury. *JCI Insight*. **4**, e125490 (2019).

[R12] Sis, B. *et al*. Accelerated expression of senescence associated cell cycle inhibitor p16INK4A in kidneys with glomerular disease. *Kidney Int*.**71**, 218–226 (2007).

[R13] Verzola, D. *et al*. Accelerated senescence in the kidneys of patients with type 2 diabetic nephropathy. *Am. J. Physiol. Renal Physiol*. **295**, F1563–F1573 (2008).
[R14] Liu, J. *et al*. Accelerated senescence of renal tubular epithelial cells is associated with disease progression of patients with immunoglobulin A (IgA) nephropathy. *Transl. Res.* **159**, 454–463 (2012).

Reviewer #3 (Remarks to the Author):

The authors have extensively responded to the critiques raised in the initial review. These have alleviated most of the concerns. I have one comment and one critique that needs to be addressed.

Response: We thank the reviewer for positive evaluation of our work and effort in revision.

1. In response to critique #1, the specificity of the Ksp-Cre expression. We appreciate that the authors went at length to demonstrate that Ksp-Cre is in fact expressed triggering recombination in all the nephron segments. Of note, their argument that it is strongly expressed in proximal tubules in the adult mice is incorrect. The cited figure S1C in PMID: 32127410 does not show expression in proximal tubules and only low expression in the loops. But since the authors do not use an inducible version of the Ksp-Cre, this is not critical for the manuscript.

Response: We thank the reviewer for bringing this concern to our attention, and agree that Ksp expression is relatively low in proximal tubules as compared to the higher levels in the collecting ducts, loops of Henle, and distal tubules. We apologize for the inaccurate description for Ksp expression in proximal tubules concerning PMID 32127410 in last response. This citation primarily aims to explain that Ksp expression is detected in the developing kidney, and increases as the kidney matures.

2. In response to critique #4, the authors state that "As suggested by the reviewer, we have also refrained from the inappropriate use of G2/M cell cycle arrest in the revised manuscript." Yet there are still 10 instances in the manuscript (including the abstract), where it is still referred to as G2/M cell cycle arrest. These must be changed. Of note, phospho-Histone H3 is not a marker for G2/M cell cycle arrest but simply indicates that cells are in late G2 or M phase of the cell cycle. Finally, in their response the authors mentioned the presence of p16 and p21. the mentioned cell cycle inhibitors (p16, p21) arrest the cell cycle in G1/S not G2/M. These further questions the

interpretation of a G2/M arrest.

Response: We thank the reviewer for bringing this critical point to our attention. As suggested by the reviewer, we have now refined “G2/M cell cycle arrest” to appropriate description thoroughly in the revised manuscript.

Dear Dr Zang,

Thank you again for the transfer of your revised manuscript (EMBOJ-2026-124426-T) to The EMBO Journal, as well as for your patience with our feedback from the arbitration process. We have carefully assessed your manuscript, and the point-by-point response provided to the referee concerns that were raised during peer-review at a different journal. In addition, and as mentioned before, we decided to send the revised version of your work back to seek original reviewer input with respect to technical robustness, conceptual advance and overall suitability of your work for publication in The EMBO Journal. We have now received a re-report by former referee #1 which I enclose below. As you will see from the expert's remarks, s/he states that the study is still consolidated by the amendments and is supportive of publication at our journal.

We are thus pleased to inform you that your study has been provisionally accepted for publication at The EMBO Journal.

We now need you to take care of a number of minor issues related to formatting and data annotation, as to the policies of our journal, which I will share shortly in a separate message, together with additional changes and requests by our production team for Source Data provision.

Please submit a revised version of the manuscript using the link enclosed below.

As you might have seen on our web page, every paper at the EMBO Journal now includes a 'Synopsis', displayed on the html and freely accessible to all readers. The synopsis includes a 'model' figure as well as 2-5 one-short-sentence bullet points that summarize the article. I would appreciate if you could provide this figure and the bullet points.

Please let me know any time should you have additional questions regarding above points.

Thank you again for giving us the chance to consider your manuscript for The EMBO Journal, I look forward to hearing from you and receiving your final revised version of the manuscript.

Kind regards,

Daniel Klimmeck

Daniel Klimmeck PhD
Senior Editor
The EMBO Journal

Please remember: Digital image enhancement is acceptable practice, as long as it accurately represents the original data and conforms to community standards. If a figure has been subjected to significant electronic manipulation, this must be noted in the figure legend or in the 'Methods' section. The editors reserve the right to

request original versions of figures and the original images that were used to assemble the figure.

Referee #1, arbitrating re-comments:

This study investigates the role of Erbin in tubular epithelial cells and provides evidence that loss of Erbin increases susceptibility to acute kidney injury and promotes renal fibrosis. The study addresses a biologically and clinically relevant question and presents a substantial body of experimental data. My previous reviews at the previous venue focused primarily on the interpretation and presentation of several datasets rather than on the overall significance of the work.

The two issues that I had raised during the final review stage at other journal have been addressed appropriately:

1. Lifespan analysis and eGFR interpretation:

The authors have moved the lifespan data to Supplementary Figure 21i and removed the eGFR data from the main text. These revisions appropriately reduce the risk of overinterpretation and address my previous concerns regarding statistical power and interpretation.

2. Presentation of urinary albumin, urinary creatinine, and UACR:

The authors have moved urinary albumin and urinary creatinine data to Supplementary Figure 21h and retained UACR in the main figure. This presentation is appropriate and avoids potential confusion arising from urine concentration-dependent parameters.

After examining the revised version, I believe that the authors have appropriately addressed the major concerns that remained at the final review stage. The revised presentation is more balanced, and the conclusions are better aligned with the available data.

Dear Dr Zang,

Further to below, I attach the mentioned list of formatting changes required for your final resubmission.

Please let us know any time should you have additional questions related.

Best regards,

Daniel Klimmeck

Daniel Klimmeck PhD
Senior Editor
The EMBO Journal

******

Formatting changes required for the revised version of the manuscript:

>> Authors: All corresponding authors need institutional email addresses entered into our online system and accounts need the ORCID number linked (Y.Z., J.Z.). Please see below for additional information.

>> Author Contributions: Remove the author contributions information from the manuscript text. Note that CRediT has replaced the traditional author contributions section as of now because it offers a systematic machine-readable author contributions format that allows for more effective research assessment. and use the free text boxes beneath each contributing author's name to add specific details on the author's contribution.

More information is available in our guide to authors.
https://www.embopress.org/page/journal/14602075/authorguide

>> Provide a completed Author Checklist.

>> As you have seen on our webpage, every paper at the EMBO Journal includes a 'Synopsis', displayed on the html and freely accessible to all readers. The synopsis includes a 'model' figure as well as 2-5 one-short-sentence bullet points that summarize the article. I would appreciate if you could provide this figure and the bullet points.

>> Adjust the title of the 'Competing Interests' section to 'Disclosure and Competing Interests Statement'.

>> Please correct the order and headings of the manuscript sections to Abstract / Introduction / Results / Discussion / Methods / Data Availability / Acknowledgements / Disclosure and Competing Interests Statement / References / Figure Legends / Expanded View Figure Legends

>> Correct the nomenclature to Figure EV1 etc. in the expanded view figure legends

>> Figure callouts: Please ensure that the figures including EV figures are called out in sequential order.

>> Funding information: remove the additional funding section at the beginning of the manuscript text.

>> Appendix File with ToC: Please add a table of contents with page numbers and correct the nomenclature from Supplementary Table S1 to Appendix Table S1, etc. and from Suppl. Fig S1 to Appendix Figure S1 etc. Upload the final version of the file in PDF format and label it Appendix.

>> Dataset EV Legends: The authors uploaded data sets with statistical information grouped by figure - is this source data? Suppl. Table 2 and 3 should be renamed Dataset EV1 and Dataset EV2, and the corresponding legends should be removed with the suppl information file and added to the dataset files, in a separate worksheet.

>> References: please adjust reference format to EMBO Journal format, 10 authors et al.; In the text of the manuscript, a reference should be cited by author and year of publication; no more than two authors may be cited per reference; 'et al' should be used if there are more than two authors (i.e. Smith & Jones, 2003; Smith et al, 2000). In the reference list, citations should be listed in alphabetical order and then chronologically, with the authors' surnames and initials inverted; where there are more than 10 authors on a paper, 10 will be listed, followed by 'et al.'.

>> Please add a Reagents and Tools table to the Methods section, as a separate file using the existing template in the Guide For Authors, listing key reagents, experimental models, software and relevant equipment.

Please compare also our Guide-to-Authors instructions:
https://www.embopress.org/page/journal/14602075/authorguide#structuredmethods

>> Biorender: remove the info about BioRender from the figure legends and add a dedicated graphics section to the end of the Methods, following this format:

Graphics:

(some of the... OR Figure #... OR synopsis) Graphics were created with BioRender.com.

>> Please provide source data for the study as to the separate request e-mail. Source data should be uploaded as one (zipped) file per figure.

>> Data availability section: Correct the hyperlink provided for the GEO dataset and make sure dataset is made publicly accessible.

>> Consider additional changes and comments from our production team as indicated below:

- Figure legends:
1. Please note that the exact p values are not provided in the legends of figures 1A,B,C,E,F,G,I,K,L; 2B-E,G,H,I; 3B,D,E,F,H,I,J; 4C-H; 5D,E,G,H,I,J; 6C,D,E,F,G,I,K,L,M; 7A,B,D,E,F,G,I,J,L,M,N,P; 8A,B; EXTENDED DATA FIGS. 1B,C,D,F,G,H; 2B,E,F,H,I,J; 3B-D,F,G,H; 4D,E,F,G,H; 5A-J.
2. Please indicate the statistical test used for data analysis in the legends of figures 7E; EXTENDED DATA FIG. 5D.
3. Please note that information related to n is missing in the legends of figures 1G,I.
4. Please note that the error bars are not defined in the legends of figures 6C,D,E,F,G,I,K,L,M.
5. Please note that the red box is not defined in the legend of 5A. This needs to be rectified.

Further information is available in our Guide For Authors: https://link.springer.com/journal/44318/submission-guidelines

****

Please note that as of January 2016, our new EMBO Press policy asks for corresponding authors to link to their ORCID iDs. You can read about the change under "Authorship Guidelines" in the Guide to Authors here: http://emboj.embopress.org/authorguide

In order to link your ORCID iD to your account in our manuscript tracking system, please do the following:

1. Click the 'Modify Profile' link at the bottom of your homepage in our system.
2. On the next page you will see a box half-way down the page titled ORCID*. Below this box is red text reading 'To Register/Link to ORCID, click here'. Please follow that link: you will be taken to ORCID where you can log in to your account (or create an account if you don't have one)
3. You will then be asked to authorise Wiley to access your ORCID information. Once you have approved the linking, you will be brought back to our manuscript system.

We regret that we cannot do this linking on your behalf for security reasons. We also cannot add your ORCID iD number manually to our system because there is no way for us to authenticate this iD number with ORCID.

Thank you very much in advance.

The authors addressed the remaining editorial issues.

1st Revision - Editorial Decision 23rd Jul 2026

Dear Dr Zang,

Thank you for submitting the revised version of your manuscript. I have now evaluated your amended manuscript and concluded that the remaining minor concerns have been sufficiently addressed.

I am thus pleased to inform you that your manuscript has been accepted for publication in the EMBO Journal.

Your manuscript will be processed for publication by EMBO Press. It will be copy edited and you will receive page proofs prior to publication. Please note that you will be contacted by Springer Nature Author Services to complete licensing and payment information.

Please note that it is The EMBO Journal policy for the transcript of the editorial process (containing referee reports and your response letter) to be published as an online supplement to each paper. If you do NOT want this, you will need to inform the Editorial Office via email immediately. More information is available here: https://www.embopress.org/transparent-process#Review_Process

You may qualify for financial assistance for your publication charges - either via a Springer Nature fully open access agreement or an EMBO initiative. Check your eligibility: https://www.embopress.org/page/journal/14602075/authorguide#chargesguide

Should you be planning a Press Release on your article, please notify the production team via this form in order to coordinate publication and release dates: https://forms.cloud.microsoft/e/eH4A8JVP6g

If you have any questions, please do not hesitate to contact the Editorial Office. Thank you for your contribution to The EMBO Journal.

Thank you again for this contribution to The EMBO Journal and congratulations on a successful publication! Please consider us again in the future for your most exciting work.

Best regards,

Daniel Klimmeck

Daniel Klimmeck, PhD
Senior Editor
The EMBO Journal
EMBO
Postfach 1022-40
Meyerhofstrasse 1
D-69117 Heidelberg
contact@embojournal.org

*****
Please note that it is The EMBO Journal policy for the transcript of the editorial process (containing referee reports and your response letters) to be published as an online supplement to each paper. If you should prefer removal of any referee-only figures included in the point-by-point response(s), e.g. because they may still be used for future publication or because they have been reproduced from published work by others, please do let us know immediately via response email.
More information is available here: https://link.springer.com/partners/embo-press/editorial-policies#Peer%20review

EMBO Press Author Checklist

Corresponding Author Name: Yuhui Zang; Junfeng Zhang	
Journal Submitted to: EMBO Journal	
Manuscript Number: EMBOJ-2026-124426	

USEFUL LINKS FOR COMPLETING THIS FORM
[The EMBO Journal - Author Guidelines]
[EMBO Reports - Author Guidelines]
[Molecular Systems Biology - Author Guidelines]
[EMBO Molecular Medicine - Author Guidelines]

Reporting Checklist for Life Science Articles (updated January
This checklist is adapted from Materials Design Analysis Reporting (MDAR) Checklist for Authors. MDAR establishes a minimum set of requirements in transparent reporting in the life sciences (see Statement of Task: 10.31222/osf.io/9sm4x). Please follow the journal's guidelines in preparing your
Please note that a copy of this checklist will be published alongside your article.

Abridged guidelines for figures
1. Data
The data shown in figures should satisfy the following conditions:
- the data were obtained and processed according to the field's best practice and are presented to reflect the results of the experiments in an accurate and unbiased manner.
- ideally, figure panels should include only measurements that are directly comparable to each other and obtained with the same assay.
- plots include clearly labeled error bars for independent experiments and sample sizes. Unless justified, error bars should not be shown for technical
- if n<5, the individual data points from each experiment should be plotted. Any statistical test employed should be justified.
- Source Data should be included to report the data underlying figures according to the guidelines set out in the authorship guidelines on Data

2. Captions
Each figure caption should contain the following information, for each panel where they are relevant:
- a specification of the experimental system investigated (eg cell line, species name).
- the assay(s) and method(s) used to carry out the reported observations and measurements.
- an explicit mention of the biological and chemical entity(ies) that are being measured.
- an explicit mention of the biological and chemical entity(ies) that are altered/varied/perturbed in a controlled manner.
- the exact sample size (n) for each experimental group/condition, given as a number, not a range;
- a description of the sample collection allowing the reader to understand whether the samples represent technical or biological replicates (including how many animals, litters, cultures, etc.).
- a statement of how many times the experiment shown was independently replicated in the laboratory.
- definitions of statistical methods and measures:
  - common tests, such as t-test (please specify whether paired vs. unpaired), simple χ2 tests, Wilcoxon and Mann-Whitney tests, can be unambiguously identified by name only, but more complex techniques should be described in the methods section;
  - are tests one-sided or two-sided?
  - are there adjustments for multiple comparisons?
  - exact statistical test results, e.g., P values = x but not P values < x;
  - definition of 'center values' as median or average;
  - definition of error bars as s.d. or s.e.m.

Please complete ALL of the questions below.
Select "Not Applicable" only when the requested information is not relevant for your study.

Materials

Newly Created Materials	Information included in the manuscript?	In which section is the information available? (Reagents and Tools Table, Materials and Methods, Figures, Data Availability Section)
New materials and reagents need to be available; do any restrictions apply?	Not Applicable	

Antibodies	Information included in the manuscript?	In which section is the information available? (Reagents and Tools Table, Materials and Methods, Figures, Data Availability Section)
For **antibodies** provide the following information:<br>- Commercial antibodies: RRID (if possible) or supplier name, catalogue number and or/clone number<br>- Non-commercial: RRID or citation	Yes	Reagents and Tools Table

DNA and RNA sequences	Information included in the manuscript?	In which section is the information available? (Reagents and Tools Table, Materials and Methods, Figures, Data Availability Section)
**Short novel DNA or RNA including primers, probes**: provide the sequences.	Yes	Materials and Methods, Table EV1

Cell materials	Information included in the manuscript?	In which section is the information available? (Reagents and Tools Table, Materials and Methods, Figures, Data Availability Section)
**Cell lines:** Provide species information, strain. Provide accession number in repository **OR** supplier name, catalog number, clone number, and/**OR** RRID.	Yes	Materials and Methods
**Primary cultures:** Provide species, strain, sex of origin, genetic modification status.	Yes	Materials and Methods
Report if the cell lines were recently **authenticated** (e.g., by STR profiling) and tested for mycoplasma contamination.	Not Applicable	

Experimental animals	Information included in the manuscript?	In which section is the information available? (Reagents and Tools Table, Materials and Methods, Figures, Data Availability Section)
**Laboratory animals or Model organisms:** Provide species, strain, sex, age, genetic modification status. Provide accession number in repository **OR** supplier name, catalog number, clone number, **OR** RRID.	Yes	Materials and Methods
**Animal observed in or captured from the field:** Provide species, sex, and age where possible.	Not Applicable	
Please detail **housing and husbandry conditions**.	Yes	Materials and Methods

Plants and microbes	Information included in the manuscript?	In which section is the information available? (Reagents and Tools Table, Materials and Methods, Figures, Data Availability Section)
**Plants:** provide species and strain, ecotype and cultivar where relevant, unique accession number if available, and source (including location for collected wild specimens).	Not Applicable	
**Microbes:** provide species and strain, unique accession number if available, and source.	Not Applicable	

Human research participants	Information included in the manuscript?	In which section is the information available? (Reagents and Tools Table, Materials and Methods, Figures, Data Availability Section)
If collected and within the bounds of privacy constraints report on age, sex and gender or ethnicity for all study participants.	Not Applicable	

Core facilities	Information included in the manuscript?	In which section is the information available? (Reagents and Tools Table, Materials and Methods, Figures, Data Availability Section)
If your work benefited from core facilities, was their service mentioned in the acknowledgments section?	Not Applicable	

Design

Study protocol	Information included in the manuscript?	In which section is the information available? (Reagents and Tools Table, Materials and Methods, Figures, Data Availability Section)
If study protocol has been **pre-registered, provide DOI in the manuscript**. For clinical trials, provide the trial registration number **OR** cite DOI.	Not Applicable	
Report the **clinical trial registration number** (at ClinicalTrials.gov or equivalent), where applicable.	Not Applicable	

Laboratory protocol	Information included in the manuscript?	In which section is the information available? (Reagents and Tools Table, Materials and Methods, Figures, Data Availability Section)
Provide DOI OR other citation details if **external detailed step-by-step protocols** are available.	Not Applicable	

Experimental study design and statistics	Information included in the manuscript?	In which section is the information available? (Reagents and Tools Table, Materials and Methods, Figures, Data Availability Section)
Include a statement about **sample size** estimate even if no statistical methods were used.	Yes	Figures Legends
Were any steps taken to minimize the effects of subjective bias when allocating animals/samples to treatment (e.g. **randomization procedure**)? If yes, have they been described?	Not Applicable	
Include a statement about **blinding** even if no blinding was done.	Yes	Materials and Methods
Describe **inclusion/exclusion criteria** if samples or animals were excluded from the analysis. Were the criteria pre-established?<br><br>If sample or data points were omitted from analysis, report if this was due to attrition or intentional exclusion and provide justification.	Not Applicable	
For every figure, are **statistical tests** justified as appropriate? Do the data meet the assumptions of the tests (e.g., normal distribution)? Describe any methods used to assess it. Is there an estimate of variation within each group of data? Is the variance similar between the groups that are being statistically compared?	Yes	Materials and Methods, Figures Legends

Sample definition and in-laboratory replication	Information included in the manuscript?	In which section is the information available? (Reagents and Tools Table, Materials and Methods, Figures, Data Availability Section)
In the figure legends: state number of times the experiment was **replicated** in laboratory.	Yes	Figure Legends
In the figure legends: define whether data describe **technical or biological replicates**.	Yes	Figure Legends

Ethics

Ethics	Information included in the manuscript?	In which section is the information available? (Reagents and Tools Table, Materials and Methods, Figures, Data Availability Section)
Studies involving **human participants**: State details of **authority granting ethics approval** (IRB or equivalent committee(s), provide reference number for approval.	Not Applicable	
Studies involving **human participants**: Include a statement confirming that **informed consent** was obtained from all subjects and that the experiments conformed to the principles set out in the WMA Declaration of Helsinki and the Department of Health and Human Services Belmont Report.	Not Applicable	
Studies involving **human participants:** For publication of **patient photos**, include a statement confirming that consent to publish was obtained.	Not Applicable	
Studies involving experimental **animals**: State details of **authority granting ethics approval** (IRB or equivalent committee(s), provide reference number for approval. Include a statement of compliance with ethical regulations.	Yes	Materials and Methods
Studies involving **specimen and field samples:** State if relevant **permits** obtained, provide details of authority approving study; if none were required, explain why.	Not Applicable	

Dual Use Research of Concern (DURC)	Information included in the manuscript?	In which section is the information available? (Reagents and Tools Table, Materials and Methods, Figures, Data Availability Section)
Could your study fall under dual use research restrictions? Please check biosecurity documents and list of **select agents and toxins** (CDC): https://www.selectagents.gov/sat/list.htm	Not Applicable	
If you used a select agent, is the security level of the lab appropriate and reported in the manuscript?	Not Applicable	
If a study is subject to dual use research of concern regulations, is the name of the **authority granting approval and reference number** for the regulatory approval provided in the manuscript?	Not Applicable	

Reporting

The MDAR framework recommends adoption of discipline-specific guidelines, established and endorsed through community initiatives. Journals have their own policy about requiring specific guidelines and recommendations to complement MDAR.

Adherence to community standards	Information included in the manuscript?	In which section is the information available? (Reagents and Tools Table, Materials and Methods, Figures, Data Availability Section)
State if relevant guidelines or checklists (e.g., **ICMJE, MIBBI, ARRIVE, PRISMA**) have been followed or provided.	Not Applicable	
For **tumor marker prognostic studies,** we recommend that you follow the **REMARK** reporting guidelines (see link list at top right). See author guidelines, under 'Reporting Guidelines'. Please confirm you have followed these guidelines.	Not Applicable	
For **phase II and III randomized controlled trials**, please refer to the **CONSORT** flow diagram (see link list at top right) and submit the CONSORT checklist (see link list at top right) with your submission. See author guidelines, under 'Reporting Guidelines'. Please confirm you have submitted this list.	Not Applicable	

Data Availability

Data availability	Information included in the manuscript?	In which section is the information available? (Reagents and Tools Table, Materials and Methods, Figures, Data Availability Section)
Have **primary datasets** been deposited according to the journal's guidelines (see 'Data Deposition' section) and the respective accession numbers provided in the Data Availability Section?	Yes	Data Availability Section
Were **human clinical and genomic datasets** deposited in a public access-controlled repository in accordance to ethical obligations to the patients and to the applicable consent agreement?	Not Applicable	
Are **computational models** that are central and integral to a study available without restrictions in a machine-readable form? Were the relevant accession numbers or links provided?	Not Applicable	
If publicly available data were reused, provide the respective **data citations in the reference list**.	Not Applicable	
